# Human Dental Pulp Stem Cell Secretome Restores Ischemic Stroke–Impaired Motor and Cognitive Functions by Reprogramming Redox and Inflammatory Signaling

**DOI:** 10.1002/advs.76717

**Published:** 2026-07-23

**Authors:** Kyung‐Joo Seong, Sehoon Park, Sun‐Woong Bae, Daseul Kim, Jin Ho Lee, Won‐Seok Choi, Zee‐Yong Park, Ji‐Yeon Jung, Won‐Jae Kim

**Affiliations:** ^1^ Dental Science Research Institute Stem Cell Secretome Research Center Hard‐tissue Biointerface Research Center Department of Oral Physiology School of Dentistry Chonnam National University Gwangju Republic of Korea; ^2^ GIST InnoCORE AI‐Nano Convergence Institute for Early Detection of Neurodegenerative Diseases Gwangju Institute of Science and Technology Gwangju Republic of Korea; ^3^ Department of Life Sciences Gwangju Institute of Science and Technology Gwangju Republic of Korea; ^4^ School of Biological Sciences and Technology Chonnam National University Gwangju Republic of Korea

**Keywords:** dental pulp stem cell secretome, ischemic stroke, microglial polarization, neuroinflammation, redox signaling, synaptic remodeling

## Abstract

Ischemic stroke induces oxidative stress, neuroinflammation, neuronal death, and synaptic dysfunction, leading to persistent motor and cognitive deficits. The human dental pulp stem cell (hDPSC) secretome is a promising cell‐free therapeutic candidate containing neurotrophic, antioxidant, and immunomodulatory factors. Here, we investigated its therapeutic effects in a photothrombotic mouse model of ischemic stroke and CoCl_2_‐induced hypoxic BV2 microglial cells. Proteomic profiling identified antioxidant‐associated proteins, including SOD2, GSR, and GSTP1, and microglial phenotype‐related candidates, including GRN, CSF1, and LRP1. hDPSC secretome treatment reduced stroke‐induced infarct volume and attenuated stroke‐increased neuronal apoptosis, neuronal ROS accumulation, and NF‐κB‐associated inflammatory signaling in the cortex and hippocampus. It also shifted microglial marker expression toward an M2‐associated profile and improved stroke‐impaired hippocampal neurogenesis, vascular remodeling, and synaptic organization. Proteomic analyses further identified coordinated changes in pathways related to oxidative phosphorylation, inflammatory responses, calcium signaling, SNARE‐associated vesicular transport, and ROBO–Rho‐associated cytoskeletal remodeling. These molecular and cellular changes were associated with improved motor coordination, spatial learning and memory, contextual memory, and anxiety‐like behavior. These findings support the hDPSC secretome as a cell‐free therapeutic candidate for post‐stroke functional recovery linked to redox, inflammatory, neurovascular, and synaptic remodeling.

## Graphical abstract

Human dental pulp stem cell secretome emerges as a cell‐free therapeutic strategy for ischemic stroke by reprogramming redox and inflammatory signaling. hDPSC secretome‐derived antioxidant and immunomodulatory factors suppress the TLR4–NOX–ROS–NF‐κB axis, reduce microglial inflammatory responses and apoptosis, and promote neurogenesis, angiogenesis, synaptic recovery, and motor and cognitive functional restoration.

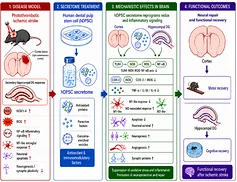

## 1 Introduction

Ischemic stroke causes persistent neurological deficits beyond the acute vascular event, including long‐lasting motor dysfunction, cognitive deficits, and reduced quality of life [1, 2]. Although current interventions offer partial protection in the acute phase, their impact on delayed neuronal injury after reperfusion remains limited. Secondary injury mechanisms, including oxidative stress, microglial activation, and sustained neuroinflammation, which impair functional recovery, are major barriers to effective therapeutic outcomes [3, 4].

The rapid elevation of reactive oxygen species (ROS) levels after cerebral ischemia is driven by multiple enzymatic and mitochondrial sources, including NADPH oxidase (NOX) isoforms, particularly NOX4, and is further amplified by Toll‐like receptor 4 (TLR4)‐associated inflammatory signaling [5, 6]. Consequently, ROS accumulation exceeds endogenous antioxidant defenses, including glutathione metabolism, superoxide dismutase (SOD), and the nuclear factor erythroid 2‐related factor 2 (Nrf2)–Heme oxygenase (HO)‐1 axis, leading to persistent redox imbalance, disrupted mitochondrial function, and accelerated neuronal death [7]. Although these antioxidant pathways are activated after ischemia, the endogenous response is often insufficient to fully restore redox homeostasis [8]. The resulting oxidative stress drives microglial polarization to the proinflammatory M1 state, which exacerbates tissue damage while suppressing the reparative M2 phenotype, thereby limiting anti‐inflammatory signaling and impairing neurogenesis and synaptic recovery [9, 10]. Taken together, the dysregulation of the TLR4–NOX–ROS axis, along with the dominance of M1 microglial activation, is a major driver of secondary injury, limiting functional recovery after stroke.

Although cortical injury is closely linked to motor impairment and disrupted synaptic plasticity [11, 12], accumulating evidence indicates that hippocampal neurogenic and synaptic pathways are also vulnerable to ischemia‐associated oxidative and inflammatory stress, contributing to post‐stroke cognitive decline [13, 14]. Therefore, recovery of hippocampal neurogenesis and synaptic connectivity is important for restoring learning and memory functions after ischemic injury [13, 14]. Guidance cue pathways, such as slit guidance ligand 2 (SLIT2) and roundabout guidance receptor 1 (ROBO1), which orchestrate cytoskeletal organization and the structure of pre‐ and post‐synaptic compartments, shape post‐ischemic synaptic remodeling [15]. Therapeutic approaches that reduce oxidative stress and promote an anti‐inflammatory microglial phenotype, along with synaptic restoration, can facilitate the structural repair and recovery of neural network function in the cortex and hippocampus after stroke.

Human dental pulp stem cells (hDPSCs) are dental pulp‐derived mesenchymal stem/progenitor cells with self‐renewal capacity and multilineage differentiation potential. hDPSCs have been reported to express MSC‐associated markers, including CD73, CD90, and CD105, and to differentiate toward osteogenic, adipogenic, and chondrogenic lineages [16, 17]. Consistent with these biological properties, human dental pulp‐derived cells have been shown to undergo odontogenic/odontoblastic differentiation and mineralized matrix formation under defined induction conditions [18]. In addition, hDPSCs have been shown to acquire neural‐like phenotypes under appropriate induction conditions, supporting their relevance to neural repair research [19]. Together, these characteristics support the use of hDPSCs as a biologically relevant cellular source for secretome‐based therapeutic strategies.

Although stem cell‐based therapies are promising in stroke repair, their clinical application is limited by immune incompatibility, tumorigenicity, and inefficient engraftment after transplantation [20]. These limitations have prompted the exploration of cell‐free therapeutic strategies using stem cell‐derived secretomes. Because stem cell‐derived secretomes can regulate inflammation, oxidative stress, neuronal survival, and tissue repair, they have been investigated as potential therapeutic tools for neurological disorders [21], including ischemic stroke [22]. The secretome is a complex mixture of bioactive molecules, including extracellular vesicles such as exosomes, growth factors, antioxidant enzymes, and immunomodulatory proteins, and exerts therapeutic effects mainly through paracrine signaling [23, 24]. Secretome‐based approaches have been investigated in diverse brain disease models because secreted factors and extracellular vesicle‐associated components can modulate oxidative stress, inflammatory signaling, neuronal survival, and tissue repair without the direct risks associated with cell transplantation [25, 26]. Nevertheless, secretome‐based therapies still require further standardization, particularly regarding donor‐ and culture condition‐dependent variability, identification of active therapeutic components, and quality‐control procedures for clinical translation [27, 28]. Despite these challenges, hDPSCs remain an attractive source for secretome‐based neural repair because they originate from dental pulp, which is derived from the neural crest [29]. In addition, the hDPSC secretome has been shown to attenuate hypoxia‐induced hippocampal neural injury, supporting its potential relevance to neuroprotective strategies [30]. The mechanisms by which hDPSC secretome treatment contributes to post‐stroke recovery remain incompletely understood.

This study evaluated the therapeutic potential of the hDPSC secretome in a photothrombotic mouse model of ischemic stroke. We hypothesized that the hDPSC secretome contributes to the recovery of motor and cognitive function by mitigating oxidative stress, shifting microglial activation toward a reparative phenotype, and enhancing neurogenesis and synaptic recovery. To test this hypothesis, we combined proteomic profiling with in vivo analyses of cortical and hippocampal repair and comprehensive behavioral assessments. To examine hDPSC secretome‐mediated regulation of inflammatory and metabolic pathways, we conducted complementary mechanistic studies in BV2 microglia cells exposed to CoCl_2_‐induced hypoxia.

## 2 Results

### 2.1 The hDPSC Secretome Mitigates Ischemic Stroke–Induced Infarction and Neuronal Cell Death

Proteomic profiling revealed that 299 proteins were uniquely present in the hDPSC secretome after subtracting those detected in serum‐free DMEM (Figure 1A,B). Gene ontology (GO) enrichment analysis revealed that terms associated with extracellular space, extracellular vesicles, and matrix binding, as well as biological process categories related to immunomodulation, neuroprotection, angiogenesis, and apoptosis regulation, were significantly enriched (*p* < 0.05, Figure 1C; Figure S1A; Table S1). Furthermore, the marked enrichment of GO terms linked to oxidative stress resistance, including “cellular oxidant detoxification” and “antioxidant activity”, indicated that the hDPSC secretome contains multiple extracellular and redox‐regulating components.

**FIGURE 1:**
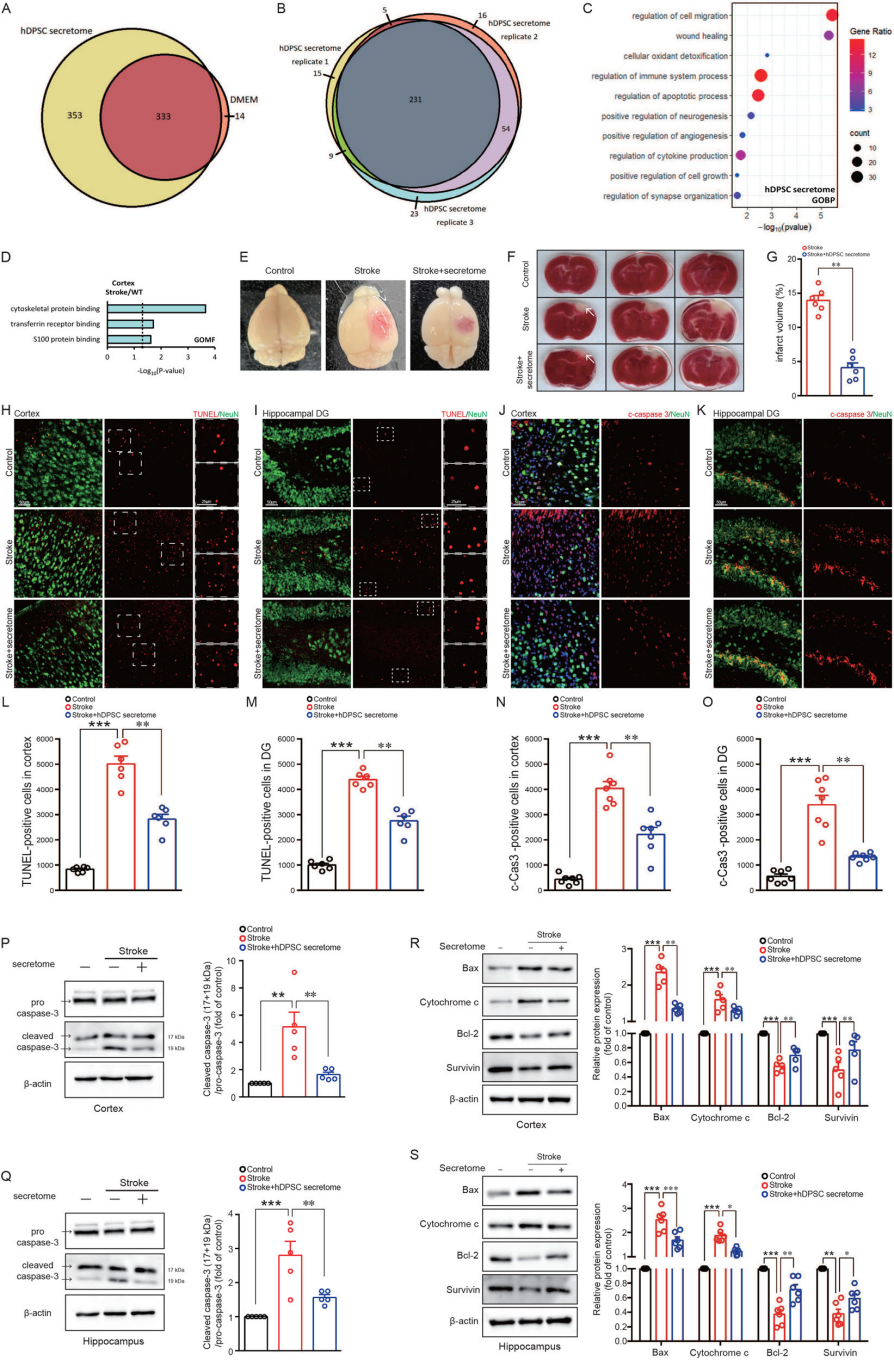
The hDPSC secretome attenuates stroke‐induced infarct volume and neuronal apoptosis in the cortex and hippocampus. (A) A Venn diagram comparing proteins detected in the hDPSC secretome and serum‐free DMEM. (B) A Venn diagram showing the proteins identified across three independent biological replicates of the hDPSC secretome. Keratin proteins and proteins detected in any DMEM replicate were excluded as contaminants. (C) GOBP enrichment analysis of 299 high‐confidence proteins detected in at least two replicates (*p* < 0.05). (D) GOMF enrichment analysis of the DEPs in the cortexes for the stroke versus WT groups (dotted line: *p* = 0.05). (E–G) Representative TTC‐stained cortical sections and infarct volume quantification three days after stroke. The white arrows indicate the infarcted areas within the cerebral cortex. (H,I) Representative images of TUNEL staining in the peri‐infarct cortex and hippocampal DG. (J,K) Representative images of cleaved caspase‐3 immunostaining in the cortex and DG. (L–O) Quantification of TUNEL‐ and cleaved caspase‐3‐positive cells in the cortex and every 10th DG section. (P–S) Representative Western blot images and quantification of the cleaved caspase‐3/pro‐caspase‐3 ratio in the cortex (P) and hippocampus (Q). Representative Western blot images and quantification of apoptosis‐related proteins, including Bax, cytochrome c, Bcl‐2, and Survivin, in the cortex (R) and hippocampus (S). Bax, cytochrome c, Bcl‐2, and Survivin levels were normalized to β‐actin, and the cleaved caspase‐3/pro‐caspase‐3 ratio was calculated. Each open circle denotes an individual biological replicate. Data are presented as mean ± SEM. Statistical significance was determined using one‐way ANOVA followed by Tukey post hoc test (*p < 0.05, **p < 0.01, ***p < 0.001). Scale bars: 25 or 50 µm (A–K).

PKH26 labeling was used to track exosome‐enriched vesicular fractions derived from the hDPSC secretome rather than all soluble and vesicular components of the whole secretome. PKH26‐positive signals were detected in the ischemic brain after intraperitoneal administration of PKH26‐labeled exosome‐enriched fractions derived from the hDPSC secretome. Western blot analysis confirmed the expression of representative extracellular vesicle markers, including CD81, CD9, and CD63, in this fraction (Figure S1B). PKH26‐positive signals were observed in SOX2‐positive neural stem cells and DCX‐positive immature neurons in the hippocampal dentate gyrus, neurofilament‐positive structures in hippocampal CA1, NeuN‐positive cortical neurons, and IBA‐1‐positive cortical microglia (Figure S1C–G). In contrast, dye‐only controls showed no detectable or only minimal red fluorescence in the hippocampal dentate gyrus (Figure S1H,I), indicating that the observed PKH26‐positive signals were not mainly caused by nonspecific dye aggregation or residual free dye. These findings suggest detectable brain delivery of PKH26‐labeled exosome‐enriched fractions derived from the hDPSC secretome to neuronal and glial cell populations in the hippocampal and cortical regions after intraperitoneal administration. However, this PKH26‐based analysis does not quantify the blood–brain barrier crossing efficiency of all soluble and vesicular components of the whole hDPSC secretome.

Cortical proteomic analysis revealed that injury‐associated proteins were elevated in mice with stroke when compared with control mice. Gene Ontology biological process (GOMF) analysis revealed that the “S100 protein binding” category, including Anxa2 and S100a6 proteins, which increase in ischemic conditions and are associated with neuronal damage [31], was significantly enriched (Figure 1D). Consistent with these molecular changes, 2,3,5‐triphenyltetrazolium chloride (TTC) staining revealed a markedly larger infarct volume in mice with stroke when compared with sham controls, and administering the hDPSC secretome substantially reduced infarct size when compared with the stroke group (Figure 1E–G).

Ischemic stroke significantly increased the number of TUNEL‐positive and cleaved caspase‐3–positive cells in both the cortex and hippocampal DG compared with the control group, whereas the hDPSC secretome treatment significantly reduced the number of apoptotic cells compared with the stroke group (Figure 1H–O). Western blot analysis further showed that the cleaved caspase‐3/pro‐caspase‐3 ratio was significantly increased in the cortex and hippocampus after ischemic stroke, whereas hDPSC secretome treatment significantly reduced this increase in both brain regions (Figure 1P,Q). Consistent with these findings, expression of Bax and cytosolic Cytochrome c was significantly upregulated, but expression of Bcl‐2 and Survivin was significantly downregulated in the stroke group compared with the control group. In contrast, compared with the stroke group, the hDPSC secretome treatment significantly restored the expression of these apoptosis‐related proteins to levels comparable to those of controls (Figure 1R,S). Together, these results indicate that the hDPSC secretome suppresses mitochondria‐associated apoptotic signaling following photothrombotic ischemic stroke.

### 2.2 The hDPSC Secretome Suppresses Ischemic Stroke‐Induced ROS Production and Restores Stroke‐Impaired Antioxidant Capacity

To assess oxidative stress following photothrombotic ischemic stroke, neuronal ROS levels were evaluated by dihydroethidium (DHE) staining colocalized with NeuN in the cortex and hippocampal DG. The number of DHE/NeuN double‐positive cells was significantly higher in the stroke group than in the control group in both regions, whereas the hDPSC secretome treatment significantly reduced these DHE/NeuN double‐positive cell counts compared with the stroke group (Figure 2A–D).

**FIGURE 2:**
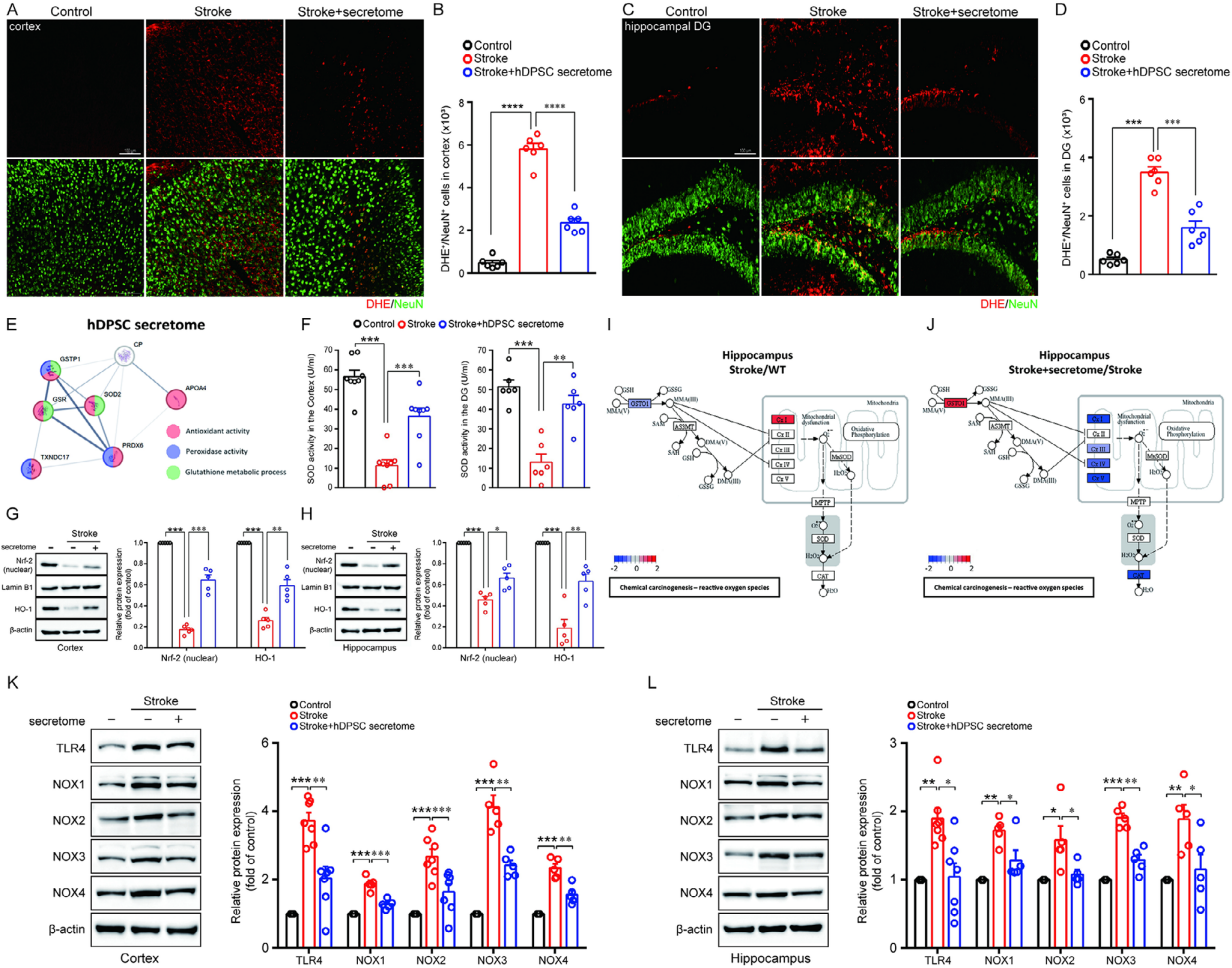
The hDPSC secretome attenuates stroke‐induced oxidative stress and restores antioxidant defense. (A,C) Neuronal ROS staining using dihydroethidium (DHE [superoxide indicator], red) co‐labeled with the neuronal marker, NeuN (green), in the peri‐infarct cortex (A) and hippocampal DG (C). (B,D) Quantification of DHE/NeuN double‐positive neurons in the cortex (B) and DG (D). (E) A PPI network of hDPSC secretome‐derived proteins associated with the GOBP term, “cellular oxidant detoxification”. Node colors indicate antioxidant activity (red), peroxidase activity (purple), and glutathione metabolism (green). (F) Superoxide dismutase (SOD) activity in the cortex and hippocampal DG. (G,H) Representative immunoblots and quantification of nuclear Nrf2 (normalized to Lamin B1) and HO‐1 (normalized to β‐actin) in the cortex (G) and hippocampal DG (H) three days after stroke. (I,J) KEGG pathway maps for “chemical carcinogenesis–reactive oxygen species” show hippocampal DEPs in stroke versus control samples (I) and the hDPSC secretome–treated stroke versus stroke (J). Node colors represent log2‐transformed fold changes. (K,L) Representative immunoblots and quantification of TLR4 and NOX1–NOX4 (normalized to β‐actin) in the cortex (K) and hippocampus (L). Open circles represent individual biological replicates. Data are presented as mean ± SEM. Statistical analyses were performed using one‐way ANOVA with Tukey's post hoc test (*p *< 0.05, *p* < 0.01, *p* < 0.001). Scale bars: 100 µm (A,C).

A protein–protein interaction (PPI) network was constructed using the antioxidant‐related proteins detected in the hDPSC secretome to identify the antioxidant components that may contribute to reduced ROS (Figure 2E). This network included SOD2, PRDX6, and glutathione‐associated enzymes, for example, GSR and GSTP1, indicating the presence of multiple enzymes involved in peroxidase activity and glutathione metabolism. Consistent with these observations, SOD activity was significantly lower in the stroke group than in the control group in both the cortex and hippocampal DG, whereas the hDPSC secretome treatment exhibited a significant restoration of SOD activity compared with the stroke group (Figure 2F). Another endogenous antioxidant signaling pathway was further assessed by examining nuclear Nrf2 and its downstream target HO‐1 in the cortex and hippocampus. The nuclear Nrf2 and HO‐1 expression were significantly reduced in the stroke group compared with the control group in both regions, whereas the hDPSC secretome treatment significantly increased nuclear Nrf2 accumulation and HO‐1 expression relative to the stroke group (Figure 2G,H).

Proteomic analysis revealed that proteins associated with the KEGG “chemical carcinogenesis–reactive oxygen species” pathway exhibited reciprocal regulation in response to ischemic stroke and hPDSC secretome treatment (Figure 2I,J). Pathway mapping showed that glutathione S‐transferase (GST), a key glutathione‐dependent antioxidant enzyme, was markedly reduced in the stroke group when compared with the control group, which was reversed by treatment with the hDPSC secretome when compared with the stroke group. In contrast, mitochondrial electron transport chain (ETC) components, which are major contributors to superoxide generation, were significantly elevated in the stroke group when compared with the control group, and treatment with the hDPSC secretome significantly suppressed these components relative to the stroke group. Moreover, expression of TLR4 and NOX1–NOX4, central enzymatic sources of ROS production, was significantly elevated in both the cortex and hippocampus following stroke, whereas the hDPSC secretome treatment substantially reduced these levels (Figure 2K,L). Together, these findings indicate that the hDPSC secretome attenuates ischemic stroke–induced oxidative stress by suppressing ROS‐generating pathways and enhancing antioxidant signaling.

### 2.3 The hDPSC Secretome Attenuates Ischemic Stroke‐Induced Neuroinflammation, With NF‐κB Signaling Suppression and Promotion of M2 Microglial Polarization

To assess microglial responses after stroke, the number of Iba‐1–positive cells was quantified in the cortex and hippocampus. The number of Iba‐1–positive cells was significantly increased in the stroke group in both regions compared with the control group. In contrast, the hDPSC secretome treatment significantly decreased the number of Iba‐1–positive cells compared with the stroke group (Figure 3A,B,F,G). Expression levels of tumor necrosis factor‐α (TNF‐α), interleukin‐1β (IL‐1β), and interleukin‐6 (IL‐6) were significantly elevated in the stroke group compared with the control group in both the cortex and hippocampus, whereas the hDPSC secretome treatment significantly reduced the levels of these cytokines relative to the stroke group (Figure 3C,D,H,I). In the cortex, expression of cyclooxygenase‐2 (COX‐2) and inducible nitric oxide synthase (iNOS) was significantly increased in the stroke group compared with the control group, whereas their expression was significantly decreased following hDPSC secretome treatment relative to the stroke group (Figure 3C,D). To further assess upstream regulatory mechanisms, nuclear factor‐κB (NF‐κB, p65) DNA‐binding activity was significantly higher in the stroke group than in the control group in both regions, whereas the hDPSC secretome treatment markedly suppressed NF‐κB DNA‐binding activity compared with the stroke group (Figure 3E,J). Together, these findings indicate that the hDPSC secretome attenuates stroke‐upregulated microglial activation and NF‐κB‐mediated inflammatory signaling in the cortical and hippocampal regions.

**FIGURE 3:**
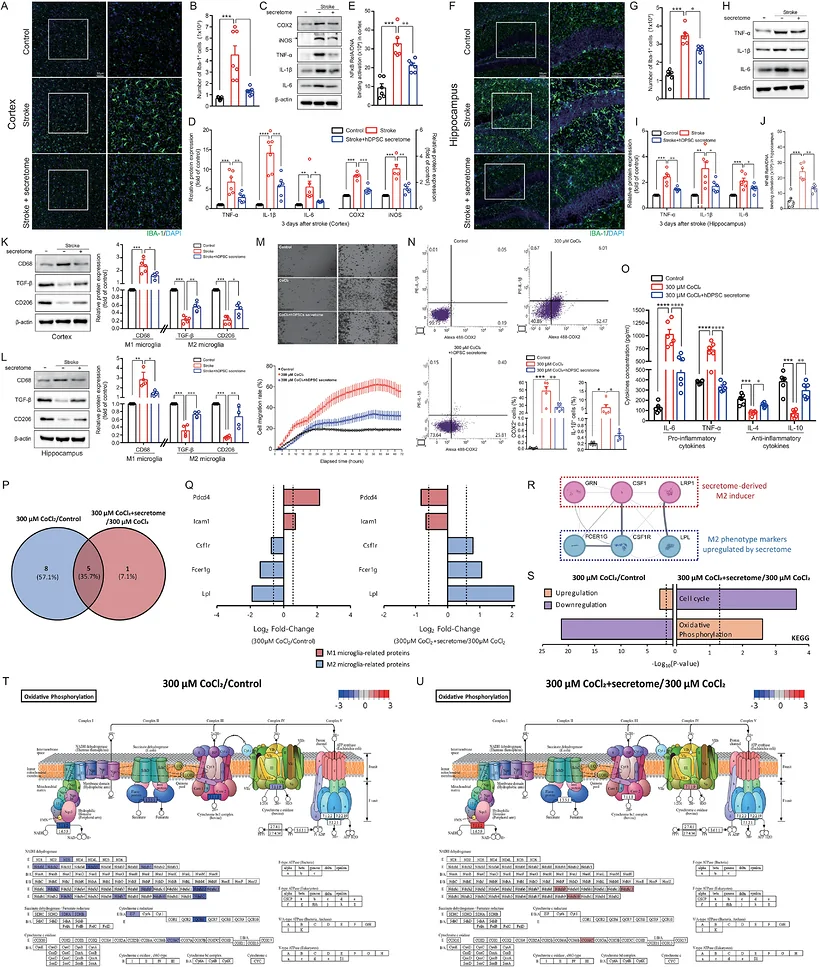
The hDPSC secretome attenuates stroke‐ and hypoxia‐induced microglial activation and inflammatory signaling while promoting M2 polarization. (A) Iba‐1 (microglial marker) immunofluorescence (green) in the peri‐infarct cortex three days after stroke. Nuclei are counterstained with DAPI (blue). (B) Quantification of Iba‐1‐positive cells in the cortex. (C,D) Western blot analysis of TNF‐α, IL‐1β, IL‐6, COX‐2, and iNOS in cortical tissue. Protein expression levels were normalized to β‐actin; quantitative analyses are shown. (E) NF‐κB p65 DNA‐binding activity in cortical nuclear extracts. (F) Iba‐1 immunofluorescence staining in the hippocampal DG. (G) Quantification of Iba‐1‐positive cells in the hippocampus. (H,I) Western blot analysis of TNF‐α, IL‐1β, and IL‐6 in hippocampal tissue. Protein expression levels were normalized to β‐actin; quantitative analyses are shown. (J) NF‐κB p65 DNA‐binding activity in hippocampal nuclear extracts. (K,L) Western blot analysis of microglial polarization markers in the cortex and hippocampus. CD68 is an M1‐associated marker, while TGF‐β and CD206 are M2‐associated markers. Protein levels were normalized to β‐actin; quantitative analyses are shown. (M) Live BV2 cell migration assays in hypoxia‐mimetic conditions (300 µm CoCl_2_ for 72 h) in the presence or absence of the hDPSC secretome (50 µg/mL). Representative migration trajectories and cumulative migration distances are shown. (N) Flow cytometric analysis of COX‐2‐ and IL‐1β‐positive BV2 cells after 24 h of hypoxia, with or without treatment with the hDPSC secretome. (O) ELISA quantification of cytokines secreted by BV2 cells. IL‐6 and TNF‐α are M1‐associated cytokines, while IL‐4 and IL‐10 are M2‐associated cytokines. (P) A Venn diagram identifying the DEPs associated with the Gene Ontology Biological Process term, “inflammatory response”, which were commonly altered by hypoxia in BV2 cells and reversed by treatment with the hDPSC secretome. (Q) Log2 fold‐change distribution of the shared DEPs from (P), categorized as M1‐ or M2‐associated proteins. Red bars indicate M1‐associated proteins. Blue bars indicate M2‐associated proteins. (R) A PPI network illustrating interactions between the hDPSC secretome‐derived M2‐inducing proteins and M2‐associated DEPs that were upregulated in BV2 cells after treatment with the hDPSC secretome in hypoxic conditions. (S) KEGG pathway enrichment analysis of BV2 cell DEPs reflecting hypoxia‐induced alterations and their modulation by treatment with the hDPSC secretome. Orange bars indicate the pathways enriched among upregulated DEPs. Purple bars indicate the pathways enriched among downregulated DEPs. Vertical dotted lines denote *p* < 0.05. (T,U) KEGG “oxidative phosphorylation” pathway diagrams showing DEPs mapped under hypoxic conditions (T) and after treatment with the hDPSC secretome under hypoxia (U). Node colors represent log2‐transformed fold protein expression changes. Each open circle represents an individual biological replicate. Data are presented as mean ± SEM. Statistical analyses were performed using one‐way ANOVA followed by Tukey's post hoc test (*p* < 0.05, *p* < 0.01, *p* < 0.001, *p* < 0.0001). Scale bars: 50 µm (A,F).

To further characterize microglial activation states, we examined M1‐ and M2‐associated polarization markers and observed that the cortical and hippocampal expression levels of cluster of differentiation (CD) 68, an M1‐associated marker, were significantly increased in the stroke group when compared with the control group, while the M2‐associated markers, transforming growth factor‐β (TGF‐β) and CD206, were significantly reduced. In contrast, the hDPSC secretome treatment significantly downregulated CD68 expression and upregulated TGF‐β and CD206 expression compared with the stroke group, which is consistent with a shift toward an M2‐associated microglial phenotype in both regions (Figure 3K,L). To visualize microglial phenotype‐associated changes at the cellular level, double immunostaining was performed for IBA‐1 with iNOS as an M1‐associated marker and IBA‐1 with Arg‐1 as an M2‐associated marker. The percentage of IBA‐1/iNOS‐double‐positive cells among IBA‐1‐positive cells was significantly increased in the cortex and hippocampus after ischemic stroke, whereas hDPSC secretome treatment significantly reduced this increase compared with the stroke group (Figure S3A,C). Conversely, the percentage of IBA‐1/Arg‐1‐double‐positive cells among IBA‐1‐positive cells was significantly decreased after ischemic stroke, whereas hDPSC secretome treatment significantly increased this percentage in both regions (Figure S3B,D).

To determine whether the in vivo effects reflect direct microglial responses, BV2 microglia were treated with increasing concentrations of CoCl_2_ to mimic hypoxic stress. CoCl_2_ exposure reduced cell viability in a dose‐dependent manner, with a pronounced reduction observed at 300 µm (Figure S2A). Treatment with the hDPSC secretome alone did not alter BV2 cell viability across the tested concentrations (Figure S2B). Accordingly, BV2 microglia were exposed to 300 µm CoCl_2_ and co‐treated with the hDPSC secretome, which partially restored cell viability, leading to the selection of 50 µg/mL for subsequent experiments (Figure S2C). Consistent with this protective effect, exposure to 300 µm CoCl_2_ markedly increased mitochondrial ROS levels, as indicated by elevated MitoSOX fluorescence, whereas the hDPSC secretome treatment significantly attenuated this increment compared to the CoCl_2_‐treated group in BV2 microglia (Figure S2D). Additionally, the levels of the mitochondrial fusion protein, Mfn2, and the antioxidant enzyme, SOD1, which were reduced by CoCl_2_, were significantly restored by treating BV2 cells with the hDPSC secretome (Figure S2E,F). Moreover, HIF‐1α expression was significantly increased under CoCl_2_‐induced hypoxic conditions compared with the control group, whereas the hDPSC secretome treatment significantly reduced HIF‐1α expression in CoCl_2_‐treated BV2 microglia (Figure S4A–C). Next, BV2 cells were treated with 300 µm CoCl_2_, after which migratory responses were monitored for 72 h, and inflammatory and metabolic responses were assessed at 24 h after CoCl_2_ exposure. Live‐cell tracking analysis showed that 300 µm CoCl_2_ exposure significantly increased BV2 cell migration over the 72 h observation period compared with the control group, whereas the hDPSC secretome treatment significantly attenuated this CoCl_2_‐induced migratory response (Figure 3M). Flow cytometry revealed that treatment with 300 µm CoCl_2_ for 24 h significantly increased the proportions of COX‐2‐ and IL‐1β‐positive BV2 cells compared with the control group, whereas hDPSC secretome treatment significantly reduced both populations (Figure 3N). To further evaluate microglial polarization, we performed additional flow cytometric analysis using CD86 as an M1‐associated marker and Arg1 as an M2‐associated marker. 300 µm CoCl_2_ treatment significantly increased the proportion of CD86‐positive BV2 cells and significantly reduced the proportion of Arg1‐positive BV2 cells, whereas hDPSC secretome treatment significantly reduced CD86‐positive cells and increased Arg1‐positive cells compared with the CoCl2‐treated group (Figure S2G,H).

Proteomic analysis of the differentially expressed proteins (DEPs) revealed a significant enrichment of the TNF signaling pathway in BV2 cells, with 300 µm CoCl_2_ significantly increasing the expression of pathway‐associated proteins, and treatment with the hDPSC secretome significantly reducing their expression (Figure S5). These proteomic changes were further supported by cytokine profiling results. Secretion of the M1‐associated cytokines IL‐6 and TNF‐α was significantly elevated following 300 µm CoCl_2_ exposure compared to the control group, whereas the hDPSC secretome treatment significantly reduced their secretion in BV2 microglia. In contrast, secretion of the M2‐associated cytokines IL‐4 and IL‐10 was significantly decreased under hypoxic conditions, but this decrease was significantly reversed by the hDPSC secretome treatment toward control levels (Figure 3O).

To investigate the proteomic features accompanying the M1‐ and M2‐like phenotype shifts, we analyzed DEPs associated with the gene ontology biological process (GOBP) term “inflammatory response” in BV2 cells. This analysis identified 14 DEPs that were altered by 300 µm CoCl_2_‐induced hypoxia and hDPSC secretome treatment under hypoxic conditions (Figure 3P). Among these proteins, Pdcd4, Icam1, Csf1r, Fcer1g, and Lpl were shared between the two comparative datasets, and their fold change patterns were further examined because these proteins have been reported to be associated with M1‐ or M2‐like microglial phenotypes [32, 33]. Compared with the control group, M1‐associated DEPs were increased under hypoxic conditions and reduced following hDPSC secretome treatment. In contrast, M2‐associated DEPs were decreased under hypoxic conditions and increased after hDPSC secretome treatment (Figure 3Q). Furthermore, a PPI network was constructed to explore potential links between hDPSC secretome‐derived proteins previously implicated in microglial phenotype regulation, including GRN, CSF1, and LRP1, and the M2‐associated DEPs identified in BV2 cells. This analysis revealed database‐derived interaction edges connecting these secretome‐associated proteins with M2‐related proteins, including Fcer1g, Csf1r, and Lpl (Figure 3R). These PPI‐based findings should be interpreted as candidate pathway‐level associations rather than direct evidence of functional interactions. Together, these results suggest that hDPSC secretome treatment is accompanied by inflammatory response‐related proteomic remodeling and M2‐like microglial phenotype‐associated changes in hypoxic BV2 cells.

KEGG enrichment analysis identified oxidative phosphorylation as another major pathway altered by CoCl_2_‐induced hypoxia and hDPSC secretome treatment in BV2 cells. Multiple oxidative phosphorylation‐related proteins were markedly downregulated under hypoxic conditions, whereas their expression was significantly increased following hDPSC secretome treatment (Figure 3T,U). Together, the in vivo data indicate that hDPSC secretome treatment attenuates stroke‐associated microglial activation and inflammatory responses in the cortex and hippocampus, with reduced NF‐κB‐mediated inflammatory signaling and M2‐associated microglial phenotype changes. In hypoxia‐mimetic BV2 cells, proteomic analyses further revealed inflammatory response‐related remodeling and partial recovery of oxidative phosphorylation‐associated protein expression after hDPSC secretome treatment.

### 2.4 The hDPSC Secretome Restores Ischemic Stroke‐Impaired Hippocampal Neurogenesis and Angiogenesis in the Cortex and Hippocampus

To assess the proliferation of NSCs in the hippocampal DG after ischemic stroke, we quantified 5‐bromo‐2’‐deoxyuridine (BrdU)‐positive cells. The number of BrdU‐positive cells was significantly reduced in the stroke group compared with the control group at both time points examined, whereas the hDPSC secretome treatment significantly increased the number of BrdU‐positive cells relative to the stroke group (Figure 4A–C). In addition, the BrdU‐positive cells were also SOX2‐positive, indicating that they were type 2a NSCs (Figure S6) [34].

**FIGURE 4:**
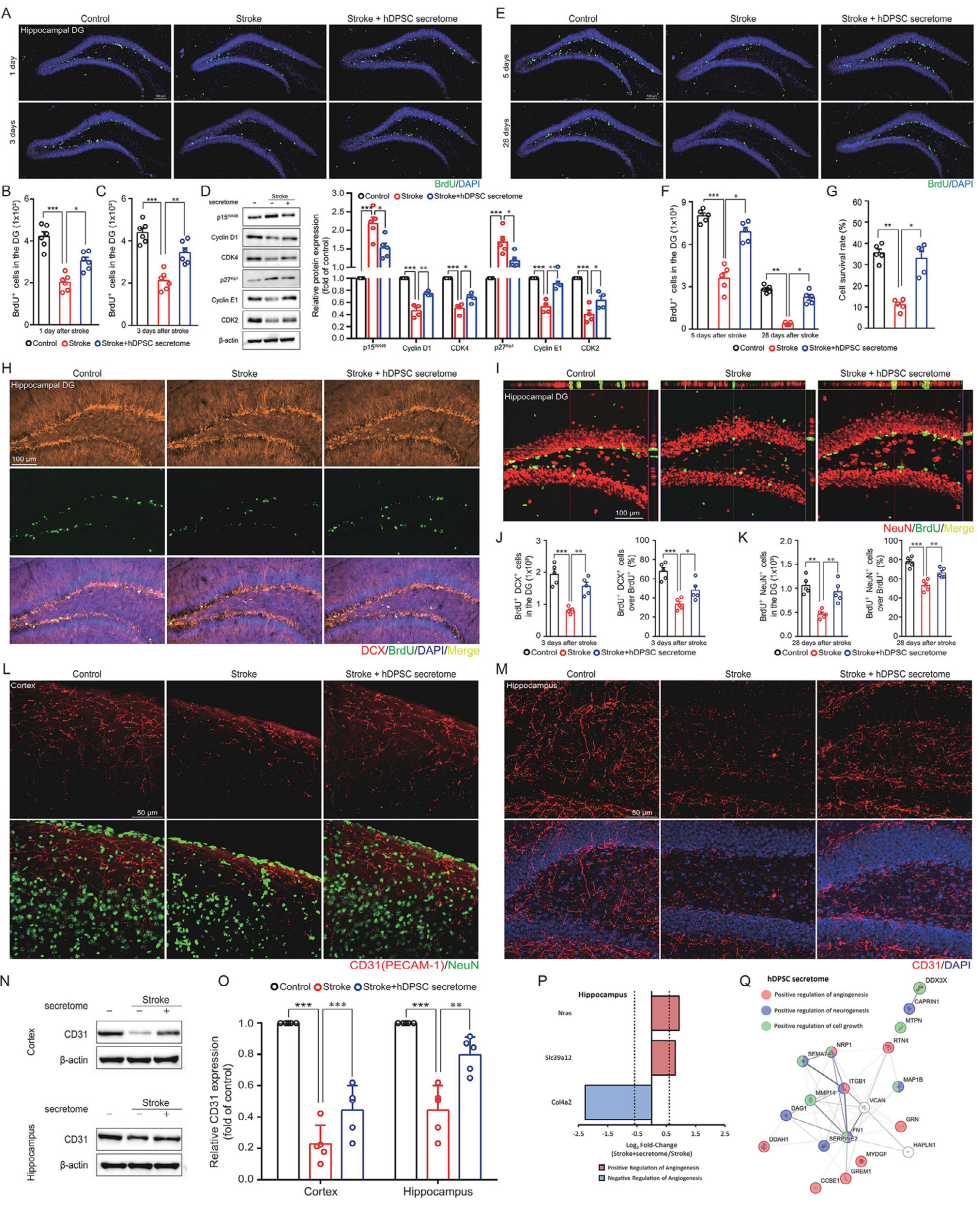
The hDPSC secretome attenuates ischemic stroke‐induced impairments in hippocampal neurogenesis and angiogenesis in the cortex and hippocampus. (A–C) BrdU (S‐phase marker) immunostaining (green) in the hippocampal DG one and three days after ischemic stroke, with DAPI counterstaining (blue). The quantification of BrdU^+^ cells is shown in (B,C). (D) Western blot analysis of cell‐cycle regulators (Cyclin D1, CDK4, Cyclin E1, and CDK2) in hippocampal lysates. Protein expression levels were normalized to β‐actin; quantitative analyses are shown. (E–G) BrdU labeling in the DG five and 28 days after stroke. The quantification of BrdU^+^ cells and the NSC survival ratio (BrdU^+^ cells at day 28 divided by BrdU^+^ cells at day 5) is shown. (H,J) Double immunofluorescence staining for BrdU (green) and the immature neuronal marker, DCX (red), three days after stroke. Quantification of BrdU^+^/DCX^+^ immature neurons and the corresponding differentiation rate is shown in (J). (I,K) Double immunofluorescence staining for BrdU (green) and the mature neuronal marker, NeuN (red), 28 days after stroke. Quantification of BrdU^+^/NeuN^+^ mature neurons and the corresponding differentiation rate is shown in (K). (L,M) CD31 (PECAM‐1) immunostaining (red) in the cortex and DG three days after stroke shows reduced vascular density after stroke and restoration following treatment with the hDPSC secretome. The nuclei are counterstained with DAPI (blue). (N,O) Representative immunoblots and quantification of CD31 in the cortex and hippocampus. Protein expression levels were normalized to β‐actin. (P) Log2‐transformed fold changes of the hippocampal DEPs associated with positive or negative angiogenesis regulation in the stroke group treated with the hDPSC secretome versus the stroke group. (Q) A PPI network of hDPSC secretome‐derived proteins associated with “positive regulation of angiogenesis” (red), “positive regulation of neurogenesis” (purple), and “positive regulation of cell growth” (green). Open circles represent individual biological replicates. Data are presented as mean ± SEM. Statistical analysis was performed using one‐way ANOVA with Tukey's post hoc test (*p* < 0.05, *p* < 0.01, *p* < 0.001). Scale bars: 100 µm (A,E,H,I) and 50 µm (L,M).

To explore mechanisms associated with enhanced NSC proliferation, key cell cycle regulators were analyzed in the hippocampal DG. The expression of cell cycle enhancers, including Cyclin D1, CDK4, Cyclin E1, and CDK2, was significantly reduced in the stroke group compared with the control group, whereas the expression of the cell cycle inhibitors p15^INK4B^ and p27^Kip1^ was significantly increased. In contrast, hDPSC secretome treatment significantly increased the expression of cell cycle enhancers and reduced the expression of cell cycle inhibitors compared with the stroke group. These changes are consistent with the recovery of G1/S phase‐associated cell cycle progression in the hippocampal DG after ischemic stroke.

To assess long‐term survival of newborn cells, BrdU‐positive cells were quantified at 5 and 28 days after ischemic stroke. The number of BrdU‐positive cells was significantly decreased in the stroke group at both time points compared with the control group, whereas the hDPSC secretome treatment significantly increased BrdU‐positive cell numbers relative to the stroke group (Figure 4E,F). The NSC survival rate, calculated as the ratio of BrdU‐positive cells at 28 days to those at 5 days, was significantly reduced in the stroke group but was significantly increased following the hDPSC secretome treatment compared with the stroke group (Figure 4G). These findings indicate that the hDPSC secretome attenuates ischemic stroke‐induced reduction in NSC proliferation and long‐term survival in the hippocampal DG.

To assess neuronal differentiation, BrdU‐positive cells were co‐labeled with DCX (immature neuron) or NeuN (mature neuron) in the hippocampal DG. At 3 days after ischemic stroke, the number of BrdU/DCX‐positive cells was significantly reduced in the stroke group compared with the control group, whereas the hDPSC secretome treatment significantly increased the number of these cells relative to the stroke group. In parallel, the proportion of BrdU‐positive cells differentiating into DCX‐positive immature neurons was significantly decreased after stroke compare to the control group but significantly increased following hDPSC secretome treatment compare to the stroke group (Figure 4H,J). At 28 days after stroke, both the number and proportion of BrdU/NeuN‐positive mature neurons were significantly reduced in the stroke group compared with the control group, whereas the hDPSC secretome treatment significantly increased both measures relative to the stroke group, approaching control levels (Figure 4I,K). Together, these results indicate that the hDPSC secretome attenuates ischemic stroke‐induced impairment of neuronal differentiation from NSCs in the hippocampal DG.

To assess vascular remodeling after ischemic stroke, we evaluated the cortical and hippocampal DG levels of CD31, an endothelial cell marker involved in vascular integrity and angiogenesis. This analysis revealed that in both regions, CD31‐positive vascular staining was reduced in the stroke group when compared with the control group, and increased by treatment with the hDPSC secretome when compared with the stroke group (Figure 4L,M). CD31 expression in the cortex and hippocampal DG was significantly decreased in the stroke group when compared with the control group, and significantly increased by treatment with the hDPSC secretome when compared with the stroke group (Figure 4N,O). Proteomic analysis of hippocampal tissue further identified changes in angiogenesis‐associated proteins. Specifically, Nras and Slc39a12, which are associated with positive regulation of angiogenesis, were upregulated, whereas Col4a2, which is associated with negative regulation of angiogenesis, was downregulated in the hDPSC secretome‐treated group compared with the stroke group (Figure 4P). In addition, PPI network analysis of the hDPSC secretome identified database‐derived interaction networks among proteins associated with vascular remodeling, neuronal support, and cellular growth (Figure 4Q). These proteomic and network‐based findings should be interpreted as candidate pathway‐level associations rather than direct evidence of functional interactions. Together, these findings indicate that hDPSC secretome treatment promotes recovery of stroke‐impaired vascular remodeling in the cortex and hippocampus, while proteomic analyses identify angiogenesis‐associated pathways as candidate molecular features linked to this vascular recovery.

### 2.5 The hDPSC Secretome Restores Stroke‐Impaired Synaptic Organization in the Cortex and Hippocampus

To examine whether hDPSC secretome treatment was linked to recovery of stroke‐impaired synaptic pathways, we analyzed synapse‐related DEPs in the cortex and hippocampus. DEPs associated with the Gene Ontology term “synaptic signaling” were visualized using heat maps and chord diagrams (Figure 5A–D). In the cortex, all ten synaptic signaling‐related DEPs were significantly upregulated in the hDPSC secretome‐treated group compared with the stroke group. In the hippocampus, thirteen of sixteen synaptic signaling‐related DEPs were significantly increased following hDPSC secretome treatment compared with the stroke group. Among these hippocampal DEPs, four were associated with positive regulation of synaptic transmission, whereas two were linked to negative regulation. Because calcium signaling is critical for neurotransmitter release and synaptic transmission, we further mapped DEPs onto the KEGG calcium signaling pathway. Several calcium signaling‐related proteins, including Ca^2^^+^/calmodulin‐dependent protein kinase, phosphodiesterase 1, and receptor‐operated calcium channels, were significantly increased in the hDPSC secretome‐treated group compared with the stroke group (Figure 5E). Notably, Ca^2^^+^/calmodulin‐dependent protein kinase, which is involved in synapsin 1 phosphorylation and synaptic vesicle mobilization [35], was increased in both the cortex and hippocampus after hDPSC secretome treatment. These proteomic findings suggest recovery of synaptic signaling‐ and calcium pathway‐associated protein expression in the post‐stroke brain.

**FIGURE 5:**
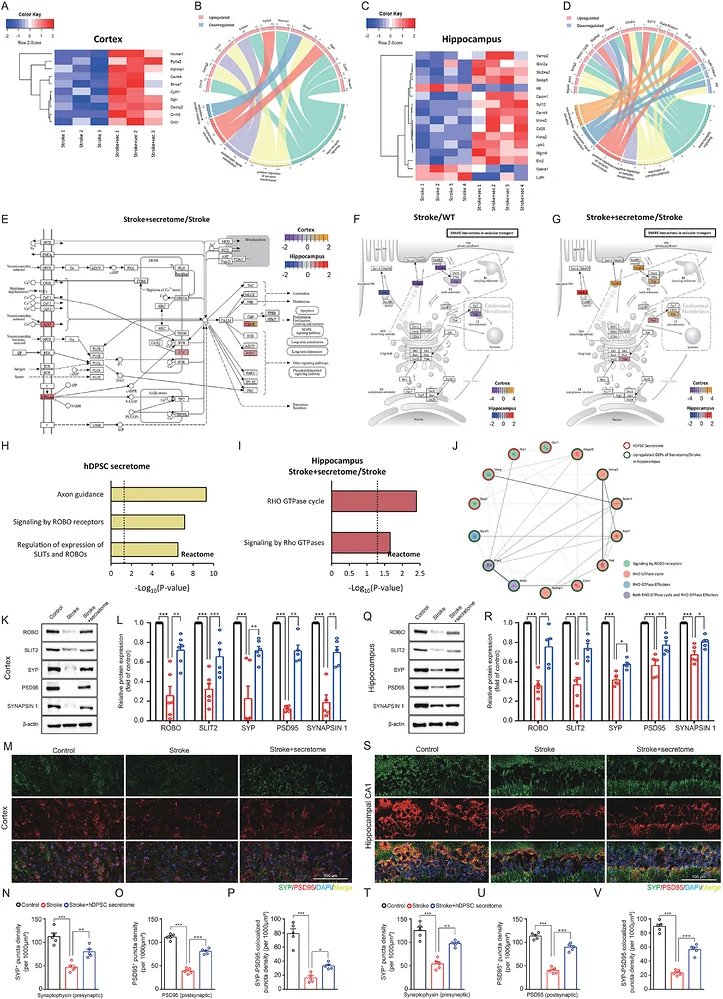
The hDPSC secretome restores ischemic stroke‐impaired synaptic protein expression and synaptic formation capacity in the cortex and hippocampus. (A,C) Heatmaps showing the relative abundance of the DEPs associated with the GOBP term, “synaptic signaling”, in the cortex (A) and hippocampus (C) of the hDPSC secretome‐treated stroke group versus the stroke group. (B,D) Chord diagrams providing detailed annotations of GOBP terms within the “synaptic signaling” category. DEPs upregulated by treatment with hDPSC secretome are shown in red, while downregulated DEPs are shown in blue. (E) KEGG pathway diagram for the “calcium signaling pathway” showing mapped DEPs from the cortex and hippocampus after treatment with the hDPSC secretome versus the stroke group. (F,G) KEGG pathway diagrams for “SNARE interactions in vesicular transport” showing mapped DEPs from the cortex and hippocampus in the stroke versus WT groups (F), and in the hDPSC secretome‐treated stroke group versus the stroke group (G). Node colors represent log2‐transformed fold protein expression changes. (H,I) Reactome pathway enrichment analysis of the proteins identified in the hDPSC secretome (H), and of hippocampal DEPs upregulated by treatment with the hDPSC secretome after stroke (I). Vertical dotted lines indicate the statistical significance threshold (*p *< 0.05). (J) A PPI network illustrating functional interactions between the hDPSC secretome‐derived ROBO signaling proteins and the hippocampal DEPs associated with Rho GTPase‐related pathways. (K–R) Western blot analysis of ROBO, SLIT2, Synaptophysin (SYP), PSD95, and Synapsin 1 in the cortex (K) and hippocampus (Q). (L,M) Protein expression levels were normalized to β‐actin; quantitative analyses are shown. (M,S) Representative immunofluorescence images showing SYP (green) and PSD95 (red) puncta in the peri‐infarct cortex (M) and hippocampal CA1 region (S). Nuclei were counterstained with DAPI (blue). Quantifications of SYP and PSD95 puncta densities are shown in panels (N,O) and panels (T,U), respectively. (P,V) Quantification of SYP/PSD95 double‐positive puncta, representing presynaptic–postsynaptic appositions, in the cortex (P) and hippocampus (V). Open circles represent individual biological replicates. Data are presented as mean ± SEM. Statistical analysis was performed using one‐way ANOVA with Tukey's post hoc test (*p* < 0.05, *p* < 0.01, *p* < 0.001). Scale bars: 100 µm (M,S).

To further investigate presynaptic vesicle trafficking‐related pathways, we analyzed KEGG SNARE interactions in vesicular transport, which are involved in synaptic vesicle docking and fusion [36]. Multiple SNARE‐associated proteins were markedly reduced in the stroke group compared with the control group (Figure 5F), whereas hDPSC secretome treatment significantly increased their expression relative to the stroke group (Figure 5G). Vesicle‐associated membrane proteins, which participate in presynaptic vesicle fusion, and Vti1, which interacts with t‐SNAREs to regulate vesicle docking specificity [36], were decreased after stroke and increased following hDPSC secretome treatment. These results suggest that hDPSC secretome treatment partially restores the expression of SNARE‐associated vesicular transport proteins disrupted after ischemic stroke.

Next, Reactome pathway enrichment analysis was performed using hDPSC secretome‐derived proteins to identify candidate pathways potentially related to synaptic recovery. This analysis revealed significant enrichment of axon guidance and ROBO receptor signaling pathways (Figure 5H). We then analyzed hippocampal DEPs that were significantly upregulated in the hDPSC secretome‐treated stroke group and found enrichment of Rho GTPase‐related pathways, which are associated with actin cytoskeleton remodeling and structural plasticity (Figure 5I). To explore potential links between these pathway groups, a PPI network was constructed by integrating ROBO‐associated proteins identified in the hDPSC secretome with Rho‐related proteins upregulated in the hippocampus. This network suggested database‐derived connectivity between secretome‐associated ROBO components and hippocampal Rho signaling‐related proteins, with non‐specific interactors excluded to improve confidence in the inferred associations (Figure 5J). These results identify ROBO receptor signaling and Rho GTPase‐associated cytoskeletal remodeling as candidate proteomic pathways linked to hDPSC secretome‐related synaptic recovery in the post‐stroke hippocampus.

To validate the synaptic alterations identified by proteomic analysis, we examined ROBO, SLIT2, synaptophysin (SYP), synapsin 1, and postsynaptic density 95 (PSD95), which are key regulators of synaptic structure and transmission, in the cortex and hippocampus. The expression of all five proteins was significantly reduced in the stroke group compared with the control group, whereas hDPSC secretome treatment significantly increased their expression relative to the stroke group (Figure 5K,L,Q,R). Consistent with these biochemical findings, immunofluorescence analysis showed marked reductions in SYP‐positive presynaptic puncta and PSD95‐positive postsynaptic puncta density in both regions after stroke. In contrast, hDPSC secretome treatment significantly restored presynaptic and postsynaptic puncta density compared with the stroke group (Figure 5M–O,S–U). Furthermore, the density of SYP/PSD95 double‐positive puncta, reflecting presynaptic‐postsynaptic apposition, was significantly decreased in the stroke group compared with the control group and significantly increased following hDPSC secretome treatment (Figure 5P,V). Together, these findings indicate that hDPSC secretome treatment restores stroke‐impaired synaptic marker expression and synaptic organization in cortical and hippocampal regions.

### 2.6 The hDPSC Secretome Restores Ischemic Stroke‐Impaired Motor and Cognitive Functions

To determine whether hDPSC secretome treatment improves functional outcomes after ischemic stroke, we first examined proteomic changes related to behavioral regulation. DEPs associated with the GO term “behavior” were significantly enriched in the cortex and hippocampus of the hDPSC secretome‐treated group compared with the stroke group (Figure 6A–D). Following hDPSC secretome treatment, six behavior‐related DEPs in the cortex and eight behavior‐related DEPs in the hippocampus were upregulated relative to the stroke group. Most of these proteins are functionally associated with learning, memory, and locomotor activity, suggesting a behavioral regulation‐related proteomic profile consistent with functional recovery after stroke.

**FIGURE 6:**
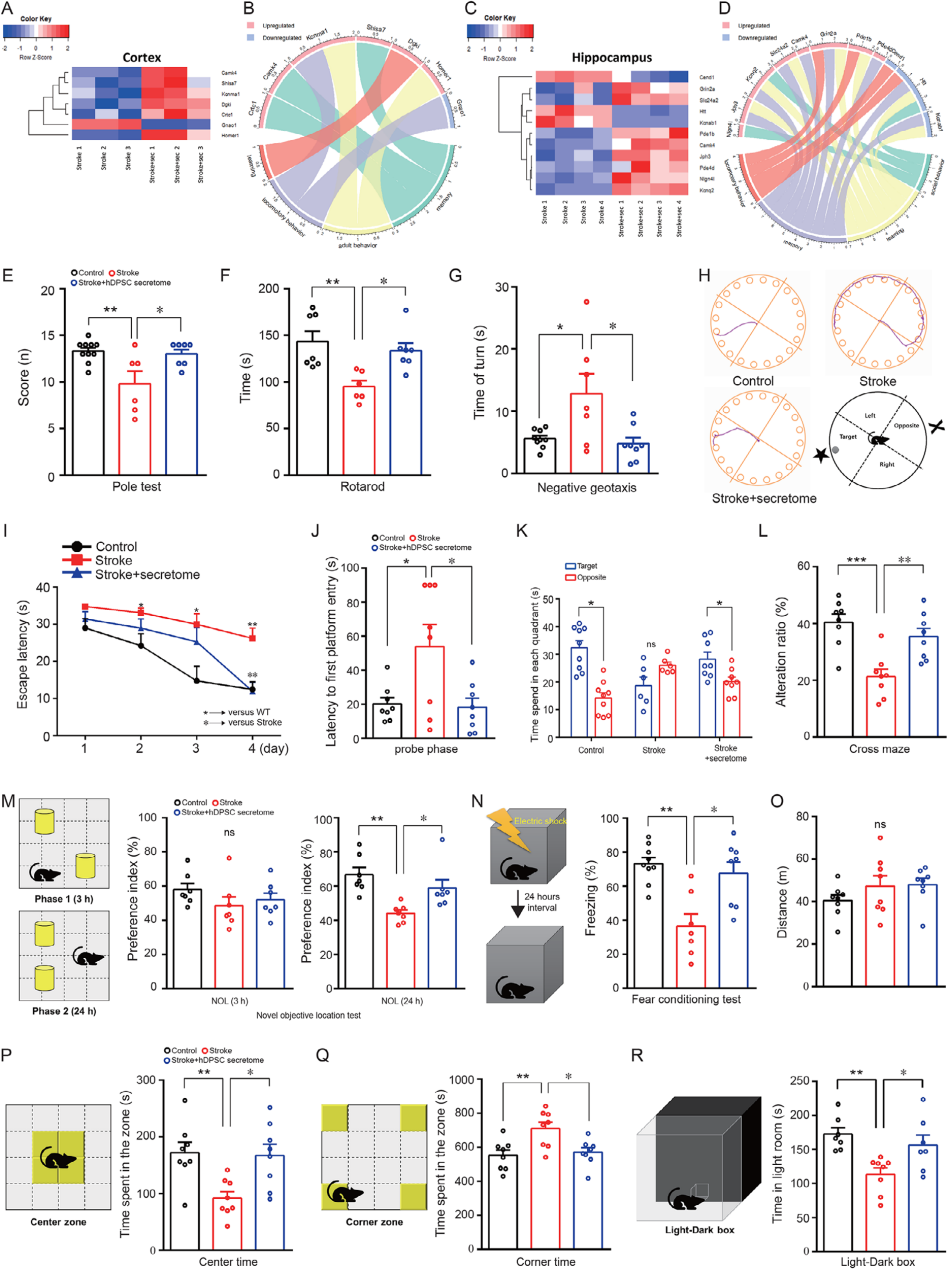
The hDPSC secretome improves motor performance and cognitive behaviors impaired by ischemic stroke. (A,C) Heatmaps showing the relative abundance of DEPs associated with the GOBP term, “behavior”, in the cortex (A) and hippocampus (C), comparing the hDPSC secretome‐treated stroke group with the stroke group. (B,D) Chord diagrams providing detailed annotations of GOBP terms in the “behavior” category. The DEPs upregulated by treatment with the hDPSC secretome relative to stroke are shown in red, while downregulated DEPs are shown in blue. (E) Pole test assessment of motor coordination and balance. (F) Rotarod test measurement of motor endurance and coordination. (G) Negative geotaxis evaluation of sensorimotor reflex and postural control. (H–K) Barnes maze assessment of spatial learning and memory. Representative movement trajectories and escape latency during training sessions are shown in (H,I). Probe test results showing latency to first target entry and the time spent in the target quadrant are presented in (J,K). (L) Cross‐maze alternation evaluation of spatial working memory. (M) Novel object location (NOL) assessment of hippocampus‐dependent spatial memory. (N) Contextual fear conditioning analysis of freezing behavior as a measure of associative memory. (O–Q) Open‐field assessment of basal locomotor activity (O) and the time spent in the center (P) and corner zones (Q). (R) Light–dark box evaluation of anxiety‐like behavior. Open circles represent individual biological replicates. Data are presented as mean ± SEM. Statistical analysis was performed using one‐way ANOVA with Tukey's post hoc test (*p* < 0.05, *p* < 0.01, *p* < 0.001).

We assessed motor coordination and balance using the pole, rotarod, and negative geotaxis tests. In the pole test, the stroke group showed a significantly reduced score compared with the control group, whereas hDPSC secretome treatment significantly increased the score relative to the stroke group (Figure 6E). In the rotarod test, the stroke group showed reduced latency to fall compared with the control group, indicating impaired balance and motor endurance, whereas hDPSC secretome treatment markedly increased fall latency (Figure 6F). In the negative geotaxis assay, the stroke group exhibited prolonged turning time compared with the control group, consistent with impaired postural reflexes, whereas the hDPSC secretome‐treated group reoriented significantly faster than the stroke group (Figure 6G).

Cognitive performance was examined using multiple hippocampus‐dependent behavioral paradigms. In the Barnes maze, the stroke group showed delayed spatial learning compared with the control group, as evidenced by prolonged escape latency during training, whereas hDPSC secretome treatment significantly improved learning acquisition (Figure 6H,I). During the probe test, the stroke group exhibited increased latency to first platform entry compared with the control group, whereas hDPSC secretome treatment significantly reduced this latency relative to the stroke group (Figure 6J). In addition, the stroke group failed to show a clear preference for the target quadrant, whereas hDPSC secretome‐treated mice spent significantly more time in the target quadrant than in the opposite quadrant (Figure 6K). Spatial working memory assessed by the cross‐maze was significantly impaired after stroke but markedly improved in the hDPSC secretome‐treated group (Figure 6L). In the novel object location task, no group difference was observed during the initial exposure phase, whereas the stroke group showed a reduced preference index 24 h later. hDPSC secretome treatment significantly increased the preference index, indicating recovery of hippocampus‐dependent spatial recognition memory (Figure 6M). Associative memory assessed by contextual fear conditioning was also reduced after stroke but significantly restored following hDPSC secretome treatment, as shown by increased freezing time (Figure 6N).

Anxiety‐related behavior was evaluated using the open‐field and light–dark box tests. Total locomotor activity was comparable across all groups (Figure 6O). However, the stroke group spent less time in the center zone and more time in the corner zones compared with the control group, indicating heightened anxiety‐like behavior. hDPSC secretome treatment significantly increased center time and reduced corner time relative to the stroke group (Figure 6P,Q). In the light–dark box test, the hDPSC secretome‐treated group spent significantly more time in the illuminated chamber than the stroke group (Figure 6R), further supporting attenuation of anxiety‐like behavior. Collectively, these behavioral outcomes indicate that hDPSC secretome treatment improves ischemic stroke‐impaired motor coordination, hippocampus‐dependent learning and memory, contextual associative memory, and anxiety‐like behavior. The comparable total locomotor activity among groups further supports that the anxiety‐related behavioral changes were not due to differences in general locomotion.

### 2.7 Integrated Proteomic Profiling Reveals Coordinated Proteomic Changes Induced by Stroke and the hDPSC Secretome Across Brain and Microglia

Principal component analysis of the cortical, hippocampal, and BV2 cell proteomic datasets revealed a clear separation of the experimental groups based on protein abundance (Figure 7A–C). Moreover, volcano plots revealed distinct DEP distributions across comparisons (Figure 7D–F). GO enrichment analysis of upregulated and downregulated DEPs revealed region‐ and condition‐specific biological responses (Figure 7G–L).

**FIGURE 7:**
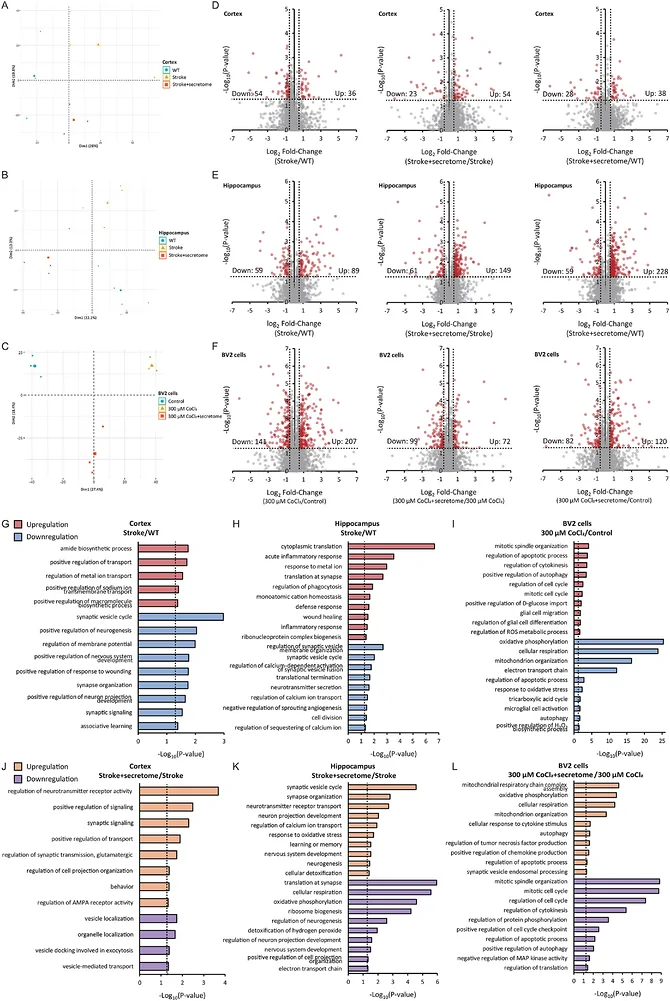
The hDPSC secretome modulates stroke‐ and hypoxia‐induced proteomic alterations in cortex, hippocampus, and BV2 microglial cells. (A–C) PCA plots showing global proteomic clustering of control, stroke, and hDPSC secretome–treated groups in the cortex (A; *n* = 3 per group), hippocampus (B; *n* = 4 per group), and BV2 microglial cells (C; *n* = 3 per group). For BV2 cells, hypoxia was induced using 300 µm CoCl_2_ in the presence or absence of the hDPSC secretome (50 µg/mL). (D–F) Volcano plots illustrating DEPs for the indicated comparisons in the cortex (D), hippocampus (E), and BV2 microglial cells (F). The *x*‐axis represents log_2_‐transformed fold changes, and the *y*‐axis represents −log_10_‐transformed *p‐*values. Vertical and horizontal dotted lines indicate the thresholds for fold change (≥1.5) and statistical significance (*p* < 0.05), respectively. Significantly regulated DEPs meeting both criteria are highlighted in red. (G–L) GOBP enrichment analysis of DEPs identified from stroke versus control comparisons (G–I) and hDPSC secretome–treated stroke versus stroke comparisons (J–L) across cortex, hippocampus, and BV2 microglial cells. Bars represent significantly enriched biological processes (*p* < 0.05, Fisher's exact test). Red and orange bars indicate processes enriched among upregulated DEPs, whereas blue and purple bars indicate processes enriched among downregulated DEPs.

In the cortex, synapse‐ and neuron‐related biological processes were reduced after stroke and increased by treatment with the hDPSC secretome (Figure 7G,J). The hippocampus exhibited a broader proteomic shift, with the DEPs upregulated in the hippocampus of the stroke versus control groups being associated with enhanced inflammatory responses (Figure 7H). This is consistent with the role of stroke in promoting localized inflammation through the release of damage‐associated molecular patterns [26]. Additionally, biological processes related to calcium ion transport were suppressed after stroke and restored by the hDPSC secretome (Figure 7H,K). Notably, ROS‐associated biological processes were significantly enriched in the hippocampus of the hDPSC secretome‐treated group versus the stroke group, indicating a role for the hDPSC secretome in redox modulation (Figure 7K).

In BV2 cells, hypoxia increased biological processes associated with cell cycle progression while suppressing mitochondrial respiration pathways (Figure 7I). These hypoxia‐driven changes were reversed by treatment with the hDPSC secretome, accompanied by partial recovery of oxidative phosphorylation‐associated proteins (Figure 7L). These molecular responses parallel in vivo findings that the hDPSC secretome reduces microglial activation, suppresses inflammatory signaling, and stabilizes metabolic function in the post‐stroke brain. Together, integrated proteomic analyses demonstrate that ischemic stroke disrupts synaptic, inflammatory, and mitochondrial pathways across brain tissue and microglia, and the hDPSC secretome drives a coordinated recovery of these systems through convergent mechanisms that promote neuronal repair and microglial homeostasis.

## 3 Discussion

In the present study, hDPSC secretome treatment was associated with molecular and cellular changes related to post‐stroke neuroprotection and repair. Proteomic profiling showed that the hDPSC secretome contains functionally distinct groups of proteins associated with antioxidant defense, immunomodulation, and tissue repair, suggesting a molecular profile that may support its neuroprotective and regenerative effects after ischemic brain injury. Sequence homology analysis further showed that several hDPSC secretome‐derived proteins are highly conserved between humans and mice (Table S2), supporting the cross‐species relevance of the hDPSC secretome in the mouse stroke model. After systemic administration, PKH26‐labeled hDPSC secretome‐derived exosome‐enriched particles were detected in the cortex and hippocampus. This observation is supported by previous reports showing that stem cell‐derived extracellular vesicles can reach injured brain tissue and exert therapeutic effects in ischemic stroke models [22, 37]. Notably, our findings support the feasibility of using a human‐derived hDPSC secretome in a mouse ischemic stroke model. Collectively, these data suggest that hDPSC secretome‐derived components reach ischemia‐affected brain regions and are associated with molecular and cellular changes related to post‐stroke neuroprotection and repair.

A previous in vitro study using HT22 hippocampal neurons showed that hDPSC secretome treatment attenuated hypoxia‐induced mitochondrial stress, including mitophagy‐associated apoptotic progression, ETC‐associated ROS generation, and NF‐κB‐related inflammatory responses [30]. The present in vivo findings extend these observations to a photothrombotic ischemic stroke model and suggest that hDPSC secretome treatment confers neuronal protection under hypoxic and ischemic conditions by reducing mitochondrial stress, inflammatory signaling, and neuronal apoptosis, thereby contributing to motor and cognitive recovery.

Neuronal apoptosis is a central pathological process contributing to infarct development and neuronal loss after ischemic brain injury. Previous studies have shown that neuronal apoptosis after cerebral ischemia is mediated, at least in part, by mitochondria‐dependent apoptotic signaling involving Bax activation, cytochrome c release, and subsequent caspase‐3 activation [38, 39, 40]. In the present study, hDPSC secretome treatment reduced stroke‐induced infarct volume and neuronal apoptosis in the cortex and hippocampal DG, supporting its neuroprotective effect after ischemic injury. This effect was reflected by decreased TUNEL‐ and cleaved caspase‐3‐positive neurons and coordinated changes in mitochondrial apoptosis‐related proteins, including reduced Bax expression and cytosolic cytochrome c levels, together with increased Bcl‐2 and survivin expression. These findings suggest that hDPSC secretome treatment attenuates ischemia‐associated intrinsic apoptotic signaling by shifting the balance of mitochondrial apoptosis‐related proteins toward neuronal survival. Although mitochondrial function and mitophagy flux were not directly assessed in the present stroke model, the observed reduction in TUNEL‐ and cleaved caspase‐3‐positive neurons, together with the regulation of Bax, cytochrome c, Bcl‐2, and survivin, supports a protective effect of the hDPSC secretome against ischemia‐associated intrinsic apoptotic signaling.

Ischemic stroke markedly increased neuronal ROS levels in the cortex and hippocampal DG, as indicated by elevated DHE/NeuN signals. This oxidative burden was associated with altered glutathione metabolism and changes in mitochondrial ETC‐related proteins, suggesting that ischemic stroke disrupts both antioxidant metabolism and mitochondrial redox regulation in neurons. hDPSC secretome treatment substantially attenuated neuronal ROS accumulation, restored total SOD activity, and increased nuclear Nrf2 accumulation together with its downstream effector HO‐1, indicating reinforcement of the Nrf2/HO‐1‐associated antioxidant defense response after ischemic stroke [7].

The redox‐modulating effect of the hDPSC secretome is supported by the presence of multiple antioxidant enzymes, including SOD2, PRDX6, GSR, and GSTP1, as identified by proteomic analysis (Table S1,2). In addition, reduced mitochondrial ROS coincided with changes in ETC‐related protein expression, suggesting regulation of mitochondrial oxidative stress‐associated pathways after hDPSC secretome treatment. Together, these findings indicate that hDPSC secretome treatment counteracts oxidative stress after ischemic stroke by enhancing antioxidant defense and regulating mitochondrial ROS‐associated pathways, thereby reducing oxidative injury in vulnerable neuronal populations.

The hDPSC secretome‐mediated suppression of ROS production may have contributed to neuronal protection and attenuated microglial inflammatory signaling and cytokine production, consistent with previous studies showing that ROS‐mediated MAPK/NF‐κB signaling promotes microglial inflammatory responses under ischemic or inflammatory conditions [41, 42]. hDPSC secretome treatment significantly reduced TLR4 expression and suppressed NOX1–NOX4 levels, indicating attenuation of the TLR4–NOX–ROS axis after ischemic stroke. Together with the reduction of ETC‐associated ROS, these changes suggest that hDPSC secretome treatment may attenuate a redox‐dependent inflammatory amplification process associated with neuronal and microglial dysfunction after ischemic stroke. These findings indicate that hDPSC secretome treatment reduces the post‐ischemic redox stress environment by suppressing TLR4–NOX–ROS signaling, reducing mitochondrial ROS accumulation, and enhancing antioxidant defense responses.

Post‐ischemic inflammatory signaling exacerbates secondary brain injury through NF‐κB‐related induction of proinflammatory cytokines, including TNF‐α, IL‐1β, and IL‐6, as well as cytotoxic mediators such as COX‐2 and iNOS [43, 44]. In the present study, ischemic stroke markedly increased Iba‐1‐positive microglial responses, as reflected by elevated Iba‐1‐positive cell numbers and increased proinflammatory cytokine expression in the cortex and hippocampus. Although Iba‐1 immunostaining provides qualitative information on microglial morphology, quantitative morphometric analysis, including soma size, process length, process thickness, and branching complexity, was not performed in this study. Therefore, the Iba‐1 immunostaining data should be interpreted as changes in the number of Iba‐1‐positive cells rather than direct evidence of microglial morphological remodeling. In contrast, hDPSC secretome treatment reduced Iba‐1‐positive cell numbers, proinflammatory cytokine expression, NF‐κB DNA‐binding activity, and COX‐2 and iNOS levels, indicating attenuation of inflammatory and cytotoxic mediator‐associated responses in post‐stroke brain regions. The reduction of NF‐κB‐associated inflammatory responses in post‐stroke brain regions supports the anti‐inflammatory effect of the hDPSC secretome under ischemic conditions. In parallel, reduced COX‐2 and iNOS expression indicates attenuation of nitric oxide‐ and prostaglandin‐associated neurotoxic stress [45, 46]. Together with the observed reduction in TLR4 and NOX1–NOX4 expression, these findings suggest that hDPSC secretome treatment suppresses interconnected components of the TLR4–NOX–ROS–NF‐κB inflammatory‐redox axis, contributing to reduced inflammation‐ and oxidative stress‐associated injury in the post‐ischemic brain.

Moreover, treatment with the hDPSC secretome modulated microglial inflammatory status toward an anti‐inflammatory and repair‐associated profile after ischemic stroke. This interpretation is supported by coordinated changes in M1‐associated markers, including CD68 and iNOS, and M2‐associated markers, including CD206, TGF‐β, and Arg1, in the post‐stroke brain. Flow cytometric analysis of BV2 cells further supported this marker‐based interpretation by showing that hDPSC secretome treatment counteracted the hypoxia‐mimetic imbalance in M1/M2‐associated markers, including CD68 and Arg1. These marker‐based changes are consistent with reported roles of proinflammatory microglia in neuronal injury exacerbation and anti‐inflammatory microglia in cellular debris clearance, angiogenesis, and neurogenesis after ischemic stroke [47, 48]. Mechanistically, these changes are consistent with coordinated suppression of TLR4–NF‐κB signaling and restoration of redox homeostasis. TLR4/NF‐κB signaling has been associated with proinflammatory microglial polarization after cerebral ischemia/reperfusion injury, whereas activation of the Nrf2–HO‐1 axis has been reported to favor anti‐inflammatory microglial responses and limit ischemic brain injury [49, 50]. By reducing the expression of NOX1 and NOX4, while enhancing Nrf2–HO‐1 signaling, the hDPSC secretome may support anti‐inflammatory microglial regulation through redox‐inflammatory signaling control. To determine whether these marker‐based changes were accompanied by microglia‐intrinsic molecular signatures, we analyzed proteomic signatures in BV2 cells under hypoxia‐mimetic conditions.

Proteomic profiling of the hDPSC secretome identified candidate proteins associated with microglial phenotype regulation, including GRN, CSF1, and LRP1. These proteins are relevant to microglial homeostasis because GRN has been linked to lysosomal and inflammatory regulation, CSF1 to microglial survival and phagocytic responses, and LRP1 to lipid metabolism and inflammatory signaling [51, 52, 53]. In the present study, PPI network analysis further suggested possible associations between these hDPSC secretome‐derived candidates and M2‐associated DEPs identified in hypoxia‐mimetic BV2 cells, including Fcer1g, Csf1r, and Lpl. These proteomic and PPI‐based findings support a candidate molecular framework related to M2‐associated microglial regulation, but they do not establish that GRN, CSF1, or LRP1 is functionally required for the observed microglial marker changes.

Microglial metabolic signatures provided additional molecular context for the M2‐associated marker changes observed after hDPSC secretome treatment. Hypoxic conditions reduced oxidative phosphorylation‐associated protein expression in BV2 cells, a pattern consistent with proinflammatory microglial activation and glycolytic reprogramming [54, 55, 56]. In contrast, hDPSC secretome treatment partially restored mitochondrial respiratory protein expression, suggesting attenuation of hypoxia‐induced metabolic disruption in microglia. Together with the marker‐based evidence, these proteomic findings support the possibility that hDPSC secretome treatment shifts microglia toward a less inflammatory and more repair‐associated state. Thus, the hDPSC secretome may contribute to reduced neuroinflammatory stress and post‐ischemic neural repair by linking redox regulation, inflammatory signaling suppression, and mitochondrial metabolism‐associated proteomic remodeling.

The photothrombotic ischemic stroke model used in this study induced a focal infarct primarily in the illuminated right somatosensory cortex, as confirmed by TTC‐based infarct assessment in the present study. Because the hippocampal DG was not directly illuminated and is located outside the primary cortical infarct area, the DG changes observed in this study should not be interpreted as primary infarct‐related damage within the hippocampus. Rather, these changes are more appropriately interpreted as secondary hippocampal responses after focal cortical ischemic injury, including neuroinflammation, oxidative stress, neurovascular alterations, apoptosis, and altered adult neurogenesis. This interpretation is supported by previous studies showing that photothrombotic and other focal cortical ischemic injuries modulate adult hippocampal neurogenesis in the DG [57, 58, 59].

Adult hippocampal neurogenesis depends on the coordinated regulation of NSC proliferation, neuronal differentiation, and maturation of newborn neurons, and this process is vulnerable to neurodegenerative and ischemic insults, including Alzheimer disease and focal cerebral ischemic injury [57, 58, 59, 60, 61, 62]. In the present study, hDPSC secretome treatment improved stroke‐impaired NSC proliferation and survival in the hippocampal DG, supporting recovery of the neurogenic niche after ischemic injury. This recovery was accompanied by increased expression of cell cycle regulators, including Cyclin D1, CDK4, Cyclin E1, and CDK2, which cooperatively regulate G1/S phase progression and NSC expansion. Proteomic profiling of the hDPSC secretome identified DDX3X within the Gene Ontology biological process “positive regulation of cell growth”. DDX3X is an ATP‐dependent RNA helicase reported to regulate cell growth through translational control of Cyclin E1 [63]. Given the role of Cyclin E1 in the G1/S transition, identification of DDX3X in the hDPSC secretome provides a candidate molecular link between secretome‐associated proteins and the recovery of cell cycle‐related neurogenic signals after ischemic injury. However, additional functional studies are required to determine whether DDX3X directly contributes to hDPSC secretome‐mediated NSC proliferation. In addition, hDPSC secretome treatment increased the persistence of BrdU‐labeled cells from 5 to 28 days after stroke, suggesting improved survival of newly generated cells during the subacute recovery phase. Together, these findings indicate that hDPSC secretome treatment supports hippocampal neurogenic recovery by enhancing NSC proliferation‐associated signaling and improving the survival of BrdU‐labeled cells after ischemic stroke.

The hDPSC secretome also supported neuronal differentiation in the hippocampal neurogenic niche. Increased numbers of BrdU/DCX‐positive immature neurons and BrdU/NeuN‐positive mature neurons indicate that newly generated cells progressed along the neuronal lineage during the recovery phase, counteracting the stroke‐induced reduction in neuronal differentiation. Because our analyses did not identify a specific hDPSC secretome‐derived factor that directly regulates neuronal differentiation, these changes are more appropriately interpreted as the formation of a permissive neurogenic microenvironment rather than the action of a single dominant molecular driver. This interpretation is further supported by previous studies showing that MSC‐derived secretomes can promote post‐ischemic neural repair through coordinated regulation of inflammation, neuronal survival, angiogenesis, and neurogenesis [37, 64]. Given that neuroinflammation is a well‐established suppressor of hippocampal neurogenesis, the pro‐neurogenic effects observed in the present study are likely linked to both differentiation‐supportive signals from the hDPSC secretome and reduced inflammatory burden in the post‐stroke hippocampus. In addition, neuroinflammation is known to suppress adult hippocampal neurogenesis, whereas inflammatory blockade or suppression of NF‐κB‐related signaling restores neurogenesis under inflammatory, stress‐related, or irradiation‐induced conditions [65, 66, 67]. Consistent with this concept, suppression of the microglial TLR4–NOX3/4–ROS–NF‐κB axis has been reported to restore hippocampal neurogenesis under X‐ray‐induced neuroinflammatory conditions [68]. Thus, the improvement in neuronal differentiation and maturation observed in the present study may reflect both differentiation‐supportive signals associated with the hDPSC secretome and reduced inflammatory burden in the post‐stroke hippocampus, thereby contributing to hippocampal neurogenic recovery after ischemic stroke.

Angiogenesis is a critical component of post‐stroke tissue repair because newly formed or remodeled vessels support oxygen and nutrient delivery and provide vascular niche‐derived trophic signals required for neurogenesis and neuronal survival [69, 70]. In the present study, hDPSC secretome treatment significantly increased CD31‐positive vascular staining and restored CD31 protein expression in the cortex and hippocampal DG, supporting recovery of stroke‐impaired vascular remodeling. Proteomic profiling of hippocampal tissue further showed that hDPSC secretome treatment increased angiogenesis‐associated proteins, including Nras and Slc39a12, and reduced Col4a2, a protein associated with negative regulation of angiogenesis. These changes are consistent with a proteomic profile favorable for vascular remodeling in the ischemic hippocampus. The vascular changes observed after hDPSC secretome treatment are also consistent with the concurrent reduction in oxidative stress and inflammatory signaling, both of which are known to impair endothelial repair and angiogenic responses after ischemic injury. Network analysis further identified hDPSC secretome‐derived proteins associated with angiogenesis‐related biological processes, suggesting candidate molecular links between the secretome composition and vascular remodeling‐associated responses. However, these network‐based findings should be interpreted as pathway‐level associations rather than direct evidence that specific secretome‐derived proteins functionally drive endothelial restoration. Together, these findings indicate that hDPSC secretome treatment supports post‐stroke vascular remodeling in parallel with hippocampal neurogenic and synaptic recovery, suggesting coordinated repair of vascular and neural compartments after ischemic stroke.

Mitochondrial dysfunction is a major contributor to synaptic failure after ischemic injury. Ischemia disrupts mitochondrial homeostasis and energy metabolism, reduces ATP production, and increases mitochondrial ROS generation, thereby impairing calcium homeostasis, synaptic vesicle cycling, and neurotransmitter release [71, 72, 73]. In the present study, hDPSC secretome treatment reduced mitochondrial ROS accumulation and increased the expression of calcium signaling‐ and SNARE‐associated proteins in the post‐stroke brain. These changes suggest that hDPSC secretome treatment supports presynaptic vesicular transport and synaptic signaling by reducing mitochondrial oxidative stress and partially restoring protein networks associated with calcium handling and vesicle trafficking. Thus, the mitochondrial redox‐regulating effect of the hDPSC secretome provides a plausible molecular context for the recovery of synaptic function after ischemic injury.

Synaptic integrity is highly vulnerable to oxidative stress and mitochondrial dysfunction because presynaptic and postsynaptic structures depend on sustained ATP production, tightly regulated calcium flux, vesicular transport, and cytoskeletal stability [73]. In this study, hDPSC secretome treatment increased the expression of ischemic stroke‐reduced synaptic markers, including synapsin 1, synaptophysin, and PSD95, in the cortex and hippocampus. Immunofluorescence analysis further showed improved presynaptic and postsynaptic puncta organization, as reflected by SYP‐, PSD95‐positive, and SYP/PSD95 double‐positive puncta. Proteomic analyses identified enrichment of calcium signaling, vesicular transport, and ROBO–Rho–actin remodeling‐related pathways in the hDPSC secretome‐treated group. Proteomic and database‐based network analyses also identified ROBO and SLIT2 as candidate proteins associated with Rho GTPase‐related networks in the post‐stroke hippocampus, suggesting possible pathway‐level associations with cytoskeletal remodeling, axonal organization, and dendritic spine stability [74, 75]. However, these proteomic and network‐based findings do not establish direct causality, and additional loss‐of‐function or pathway inhibition studies are required to determine whether SLIT2–ROBO–Rho GTPase–actin signaling directly contributes to hDPSC secretome‐mediated synaptic recovery. Together, these findings indicate that hDPSC secretome treatment supports the recovery of stroke‐impaired synaptic organization in the post‐ischemic brain and identifies calcium‐dependent signaling, vesicular trafficking, and ROBO–Rho–actin remodeling as candidate pathways associated with synaptic recovery.

The behavioral recovery observed after hDPSC secretome treatment demonstrates functional improvement of stroke‐impaired cortical‐ and hippocampus‐dependent behaviors. These improvements were accompanied by reduced oxidative stress, attenuated neuroinflammatory signaling, vascular remodeling, and restoration of synaptic marker expression in the cortex and hippocampus. Motor coordination and balance improvements in the pole and rotarod tests indicate recovery from stroke‐induced sensorimotor deficits. Given that cortical injury disrupts motor coordination, balance, and sensorimotor integration, these behavioral outcomes are consistent with post‐stroke cortical repair and synaptic organization after hDPSC secretome treatment [11, 12].

Moreover, hDPSC secretome treatment improved stroke‐impaired performance in learning and memory tasks, including the Barnes maze, novel object location, cross‐maze, and contextual fear conditioning tests. These behavioral improvements are consistent with the observed recovery of hippocampal neurogenesis, synaptic marker expression, and presynaptic‐postsynaptic organization, which are closely associated with hippocampus‐dependent cognitive functions [61, 73, 76]. Because motor execution and memory formation depend on activity‐dependent synaptic plasticity as well as neurogenic and vascular support within cortical and hippocampal networks [69, 70, 76, 77, 78], the behavioral gains observed after hDPSC secretome treatment were accompanied by coordinated recovery across redox, inflammatory, neurogenic, angiogenic, and synaptic domains. Together, these findings indicate that hDPSC secretome treatment improves functional outcomes after ischemic stroke by supporting multiple repair‐associated processes in cortical and hippocampal regions, thereby counteracting stroke‐induced motor and cognitive impairments.

In the present study, proteomic profiling revealed stroke‐associated alterations in synaptic signaling, inflammatory responses, and mitochondrial metabolism across cortical, hippocampal, and microglial compartments. Stroke markedly reduced neuronal and synaptic signaling‐related proteins while increasing inflammatory mediators and metabolic stress‐associated factors, consistent with previously reported proteomic alterations after ischemic stroke and ischemia/reperfusion injury [79, 80]. hDPSC secretome treatment partially counteracted these stroke‐associated proteomic alterations, with changes in pathways related to neuronal communication, calcium signaling, mitochondrial respiration, and inflammation‐associated processes. Principal component analysis showed that hDPSC secretome‐treated samples were separated from both the control and stroke groups. This result suggests that hDPSC secretome treatment modulated stroke‐associated proteomic changes and generated a treatment‐specific proteomic profile, rather than simply restoring the injured brain to the baseline control state. Collectively, these findings indicate that hDPSC secretome treatment is associated with coordinated proteomic remodeling across multiple injury‐related domains, including redox regulation, mitochondrial metabolism, inflammatory signaling, and synaptic organization, in the post‐ischemic brain.

Although the present study provides multi‐level evidence that hDPSC secretome treatment improves post‐stroke functional recovery and is associated with redox regulation, inflammatory suppression, microglial phenotype changes, neurovascular remodeling, and synaptic recovery, several limitations remain. Previous studies have indicated that the biological activity and therapeutic efficacy of stem cell‐derived secretomes are not always uniform across experimental settings and may differ depending on cell source, donor‐related characteristics, disease model, treatment timing, dose, and route of administration [27, 28, 81]. Therefore, the present findings should be interpreted in the context of the specific experimental conditions used in this study, including the hDPSC source, secretome preparation method, dosing regimen, route of administration, and photothrombotic ischemic stroke model. At the mechanistic level, proteomic and PPI network analyses identified candidate secretome‐derived factors and pathway‐level associations, but they do not establish direct causality. The functional requirement of candidate proteins such as SOD2, GRN, CSF1, LRP1, DDX3X, ROBO, and SLIT2 was not validated using loss‐of‐function approaches, pathway inhibition, or targeted depletion from the hDPSC secretome. In addition, although Fcer1g, Csf1r, and Lpl were identified as microglial phenotype‐associated candidates, further cellular‐level validation is needed to confirm their localization and functional relevance in microglia after ischemic stroke. Therefore, future studies using neutralizing antibodies, pharmacological inhibitors, genetic knockdown approaches, secretome component‐depletion strategies, and standardized quality‐control procedures will be necessary to define the causal contribution of individual hDPSC secretome‐derived factors and signaling pathways to redox regulation, microglial phenotype modulation, neurovascular remodeling, and synaptic repair.

## 4 Conclusion

This study demonstrates that hDPSC secretome treatment improves functional recovery after ischemic stroke and reduces stroke‐associated pathological changes in cortical and hippocampal regions. Multi‐level analyses showed that hDPSC secretome treatment decreased neuronal apoptosis, oxidative stress, and neuroinflammatory responses, while increasing antioxidant defense‐related signaling, M2‐associated microglial marker expression, vascular remodeling, hippocampal neurogenesis, and synaptic organization. Proteomic analyses further identified coordinated changes in pathways related to redox regulation, mitochondrial metabolism, inflammatory signaling, angiogenesis, calcium signaling, SNARE‐associated vesicular transport, and ROBO–Rho‐associated cytoskeletal remodeling. These findings support a model in which hDPSC secretome treatment promotes post‐stroke recovery through combined modulation of redox‐inflammatory responses, neurovascular remodeling, and synaptic repair‐associated processes. Although additional loss‐of‐function and pathway inhibition studies are required to define the causal contribution of individual secretome components and signaling pathways, the present study supports the hDPSC secretome as a promising cell‐free therapeutic candidate for functional recovery after ischemic brain injury.

## 5 Experimental Section

### 5.1 hDPSC Isolation and Culture

Teeth from twenty pediatric donors were obtained under approval from the Institutional Review Board of Chonnam National University Dental Hospital (approval no. CNUDH‐2024‐003). Written informed consent was obtained from the legal guardians before sample collection, and all procedures were conducted in accordance with the Declaration of Helsinki. Dental pulp tissues were isolated in aseptic conditions, washed with Hanks’ Balanced Salt Solution (Thermo Fisher Scientific), and minced using sterile scalpels. Cell isolation and primary culture were performed as described previously [30]. Briefly, the pellet was resuspended in Dulbecco's Modified Eagle Medium (DMEM; Thermo Fisher Scientific) supplemented with 10% fetal bovine serum (FBS, Thermo Fisher Scientific) and 100 U/mL of penicillin–streptomycin (P/S, Life Technologies). Cells were cultured in a humidified incubator at 37°C with 5% CO_2_. The expression of the hDPSC‐associated markers CD146 and STRO‐1 in hDPSCs isolated using the same protocol was previously confirmed by flow cytometry and immunocytochemistry [82]. In the present study, the hDPSCs used for secretome collection were further characterized by flow cytometric analysis of MSC‐associated surface markers, including CD73, CD90, and CD105 (Figure S10A).

### 5.2 hDPSC Secretome Collection and Preparation

hDPSC cultures at 80%–90% confluence were washed with phosphate‐buffered saline (PBS) to remove residual serum and incubated with serum‐free DMEM for 24 h. Conditioned medium was then collected and centrifuged at 300 × g for 10 min to remove detached cells. The supernatant was filtered through 0.22 µm syringe filters (Millipore), and the filtrates were concentrated using Vivaspin 20 centrifugal concentrators with a 5 kDa molecular weight cutoff (Cytiva) at 8000 × g for 20 min at 4°C. Total protein concentration in the concentrated hDPSC secretome was measured using a BCA protein assay kit (Thermo Fisher Scientific) according to the manufacturer's protocol. For all experiments, the hDPSC secretome was normalized based on total protein concentration before use. Independent hDPSC secretome batches were prepared using the same culture, collection, centrifugation, filtration, concentration, and storage procedures. Total protein concentration was measured for each batch to evaluate batch‐to‐batch reproducibility, and only total protein‐normalized preparations were used for subsequent experiments. The hDPSC secretome was aliquoted and stored at −80°C until use, and repeated freeze–thaw cycles were avoided.

For quality‐control analysis of the hDPSC secretome preparation, hDPSC cell lysates and hDPSC secretome samples were subjected to Coomassie brilliant blue G‐250 staining, Ponceau S staining, and Western blot analysis for Lamin B1. hDPSC cell lysates were used as a positive control for Lamin B1 detection. Coomassie brilliant blue G‐250 staining was performed to visualize total protein profiles after SDS‐PAGE. Ponceau S staining was performed after membrane transfer to confirm protein transfer. Lamin B1 was used as a nuclear contamination marker to assess detectable cellular contamination in the hDPSC secretome fraction (Figure S10B,C).

### 5.3 Photothrombotic Ischemic Stroke Induction and hDPSC Secretome Administration

All animal experimental procedures were approved by the Animal Care and Use Committee of Chonnam National University (approval no. CNU IACUC‐YB‐2021‐91) and were performed in accordance with institutional guidelines and national regulations for the care and use of laboratory animals. Seven‐week‐old male C57BL/6 mice (Orient Bio) were anesthetized with Zoletil 50 (Virbac) and Rompun (xylazine, Bayer), and their heads were secured in a stereotaxic frame (SR‐5N, Narishige). They were then intraperitoneally administered Rose Bengal (10 mg/mL; Sigma–Aldrich) at 20 mg/kg. The right somatosensory cortex was illuminated for 10 min using a 532‐nm light source (ZEISS KL1500 LCD) mounted on a stereotaxic workstation (Stoelting) as we described previously [59]. This photothrombotic procedure induces local microvascular thrombosis and produces a focal ischemic lesion primarily in the illuminated cortical region. Because the hippocampal DG was not directly illuminated and is located outside the primary cortical infarct area, DG analyses were performed to evaluate secondary hippocampal responses after focal cortical ischemic injury rather than primary infarct‐related damage within the hippocampus. Beginning 6 h after stroke induction, mice were intraperitoneally administered total protein‐normalized hDPSC secretome at a dose of 100 µg total protein per mouse three times at 6 h intervals. The systemic administration strategy was based on previous studies showing therapeutic effects of dental pulp stem cell‐derived secretome or conditioned medium in neurological disease models, including DPSC secretome therapy in an ALS mouse model and dental stem cell‐derived conditioned medium treatment in focal cerebral ischemia models [83, 84]. The dose was selected based on the dose‐response analysis of BrdU‐positive cells shown in Figure S7. Because the hDPSC secretome was prepared in serum‐free DMEM, a serum‐free DMEM‐treated control group was included as a vehicle control. No significant difference was detected between the untreated control and serum‐free DMEM‐treated control groups. The experimental timeline is presented in Figure S8.

### 5.4 Exosome Labeling and In Vivo Tracking

Exosome‐enriched fractions were isolated from the hDPSC‐derived secretome using ExoQuick‐TC precipitation reagent (System Biosciences) according to the manufacturer protocol. Serum‐free conditioned medium was centrifuged at 3000 × g for 15 min, mixed with ExoQuick‐TC reagent at a 5:1 volume ratio, incubated overnight at 4°C, and centrifuged at 1500 × g for 30 min. The resulting exosome‐enriched pellet was resuspended in sterile PBS, and total protein concentration was measured using a BCA protein assay kit. For fluorescent labeling, exosome‐enriched fractions were stained using a PKH26 red fluorescent linker kit (Sigma‐Aldrich). Briefly, exosome‐enriched fractions were incubated with PKH26 dye in Diluent C for 5 min at room temperature, quenched with 1% BSA, and ultracentrifuged at 100 000 × g for 1 h at 4°C to remove unbound dye. Labeled exosome‐enriched pellets were washed twice with sterile PBS and resuspended in sterile PBS. A dye‐only control was processed in parallel using the same labeling, quenching, centrifugation, and washing procedures to exclude nonspecific PKH26 dye aggregation. For in vivo tracking, mice were intraperitoneally administered PKH26‐labeled exosome‐enriched fractions at a dose of 100 µg total protein per injection. Injections were performed three times at 6 h intervals beginning 6 h after photothrombotic stroke induction, based on previous extracellular vesicle or exosome studies in brain injury models [85, 86]. Twenty‐four hours after the final injection, mice were perfused, and brains were collected for fluorescence imaging. Coronal cryosections with a thickness of 20 µm were examined using a Zeiss LSM900 confocal microscope (Carl Zeiss, Oberkochen, Germany). Fluorescent signals were analyzed using Zen 3.2 software, and figures were assembled using Adobe Photoshop CS6.

### 5.5 TTC Staining for Infarct Volume Evaluation

Mice were anesthetized using Zoletil (40 mg/kg; Virbac) and Rompun (10 mg/kg; Bayer) and perfused transcardially with 10 mL of ice‐cold PBS. Mouse brains were collected and sectioned into 1‐mm‐thick coronal slices using a mouse brain matrix (Harvard Apparatus). The sections were incubated in 0.5% TTC solution (Sigma–Aldrich) in PBS for 30 min at 37°C in the dark and then rinsed briefly with PBS. TTC‐stained sections were scanned using a digital scanner (Epson Perfection V600), and infarct areas were quantified as TTC‐negative regions using ImageJ software (NIH). To correct for brain edema, the corrected infarct area for each section was calculated by subtracting the non‐infarcted area of the ipsilateral hemisphere from the area of the contralateral hemisphere. The corrected infarct volume was obtained by summing the corrected infarct areas across serial sections and multiplying by section thickness. Infarct volume was expressed as a percentage of the contralateral hemispheric volume.

### 5.6 Brain Tissue Preparation and Immunohistochemistry

Mice were anesthetized as described above, followed by transcardial perfusion with ice‐cold PBS (10 mL), and then 10 mL of 4% paraformaldehyde (GeneAll). Next, brains were post‐fixed in 4% paraformaldehyde (24 h), cryoprotected in 30% sucrose (Sigma–Aldrich), embedded in OCT (Tissue‐Tek, Sakura), frozen on dry ice, and stored at −80°C. Coronal sections (30 µm) were obtained using a cryostat (Leica CM1950). For immunohistochemistry, sections were treated with 2 N HCl at 37°C for 30 min to retrieve BrdU antigen, neutralized, and blocked with 5% normal horse serum (Gibco). They were then incubated overnight (4°C) with primary antibodies against NeuN, cleaved caspase‐3, Iba‐1, BrdU, DCX, CD31, synaptophysin, and PSD95 (Abcam; all at 1:200). After washing with PBS, sections were incubated with Alexa Fluor 488‐ or Alexa Fluor 543‐conjugated secondary antibodies (Thermo Fisher Scientific), counterstained with DAPI (Vector Laboratories), and imaged on a Zeiss LSM900 confocal microscope (magnification: 10× or 20×).

### 5.7 Western Blot Analysis

Cortical tissues, hippocampal tissues, and BV2 cells were rinsed with ice‐cold PBS and lysed with RIPA buffer (BIOMAX) supplemented with protease inhibitors (Thermo Fisher Scientific). Lysates were incubated on ice (20 min) and cleared by centrifugation (12 000 g, 4°C) for 10 min, followed by protein quantification using a BCA assay kit (Thermo Fisher Scientific). For whole‐cell lysates, protein samples (30 µg) were mixed with the LDS sample buffer, heated at 95°C (five minutes), separated using 10%–12% SDS–PAGE, and transferred onto 0.45‐µm nitrocellulose membranes (Thermo Fisher). Next, membranes were blocked by incubating at room temperature (one hour) with 5% non‐fat milk in TBST, followed by overnight incubation (4°C) with primary antibodies against pro‐caspase‐3, cleaved caspase‐3, Bax, Cytochrome c, Bcl‐2, Bcl‐xL, survivin, Nrf2, HO‐1, TLR4, NOX1–NOX4, COX2, iNOS, TNF‐α, IL‐1β, IL‐6, TGF‐β, CD206, CD68, CD31, synaptic markers (SYP, PSD95, and Synapsin‐1), ROBO, SLIT2 (Abcam), and β‐actin (Cell Signaling) at 1:1000–1:2000 concentrations. For nuclear proteins, Lamin B1 was used as the loading control. Membranes were then washed thrice with TBST and incubated with HRP‐conjugated secondary antibodies (Cell Signaling) for one hour at room temperature, followed by signal development using a chemiluminescent substrate (Millipore), imaging on a Fusion FX system (Vilber Lourmat), and band intensity quantification using ImageMaster Assistant (Vilber).

### 5.8 DHE Staining in the Stroke Model

Brain sections were thawed, rinsed with PBS, incubated with 30 µm DHE (Sigma–Aldrich) at 37°C for 30 min in the dark, washed with PBS, and mounted using a fluorescent mounting medium. Neuronal ROS levels were assessed by co‐staining with NeuN.

### 5.9 SOD Activity Measurement

SOD activity in brain tissue was measured using the Superoxide Dismutase Colorimetric Activity Kit (Invitrogen). Tissue samples were homogenized in assay buffer and centrifuged to obtain supernatants. A standard curve was generated using SOD standards (U/mL). Reaction mixtures were incubated at 37°C, and absorbance was measured at 450 m on a microplate reader. SOD activity was expressed in units per milliliter (U/mL). The sample activity was calculated by comparing absorbance values to the standard curve.

### 5.10 BV2 Cell Culture, Hypoxia Induction, and Treatment With the hDPSC Secretome

BV2 cells were cultured at 37°C in a humidified incubator with 5% CO_2_ in high‐glucose DMEM supplemented with 10% FBS and 1% P/S. For experiments, BV2 cells were seeded in six‐well plates at a density of 1 × 10^6^ cells per well and grown to approximately 70% confluence. Based on preliminary cell viability experiments, 300 µm CoCl_2_ reduced cell viability to approximately 60%–70%, providing a suitable condition for evaluating partial recovery by hDPSC secretome treatment (Figure S2A). Hypoxia‐mimetic stress was induced by replacing the culture medium with serum‐free DMEM containing 300 µm CoCl_2_. For hDPSC secretome treatment, total protein‐normalized hDPSC secretome was added concurrently at a final concentration of 50 µg/mL. Control cells received serum‐free DMEM alone, whereas CoCl_2_‐treated cells received serum‐free DMEM containing 300 µm CoCl_2_. The CoCl_2_ + hDPSC secretome group received serum‐free DMEM containing both 300 µm CoCl_2_ and 50 µg/mL hDPSC secretome. Unless otherwise specified, all treatments were performed for 24 h under standard culture conditions.

### 5.11 Cell Viability Assay

BV2 cells were treated with CoCl_2_ or the hDPSC secretome at concentrations of 0–400 µm and 0–70 µg/mL, respectively, to determine treatment doses for subsequent experiments. For CoCl_2_, 300 µm was the lowest concentration with consistent cell viability reduction. The hDPSC secretome attenuated the 300 µm CoCl_2_‐induced viability loss at 50 µg/mL. Cell viability was assessed using the EZ‐Cytox assay (Dogen), and absorbance was measured at 450 nm on a microplate reader (BioTek Instruments).

### 5.12 Cellular ROS Measurement

BV2 intracellular ROS levels were measured 24 h after treatment with 300 µm CoCl_2_, with or without the hDPSC secretome (50 µg/mL). N‐acetylcysteine (5 µm) was applied as an antioxidant control. After treatment, cells were incubated (37°C) with 10 µm MitoSOX Red (Invitrogen) for 30 min in the dark, washed with PBS, and fluorescence was measured on a microplate reader (BioTek Instruments; excitation: 399 nm, emission: 610 nm).

### 5.13 Live Cell Migration Assay

BV2 cell migration was assessed using scratch wound‐healing assays in hypoxia‐mimetic conditions. BV2 cells (3 × 10^4^ per well) were seeded in 24‐well plates, grown to about 90% confluence, and scratched using a sterile 200‐µL pipette tip. They were then washed twice with serum‐free medium to remove detached cells, followed by treatment with 300 µm CoCl_2_ for 72 h, with or without the hDPSC secretome (50 µg/mL). Time‐lapse phase‐contrast images were acquired every 60 min for 72 h on a JuLI Stage Real‐Time Cell History Recorder (NanoEntek) in a humidified incubator (37°C, 5% CO_2_). Migration distance was quantified using the JuLI Stage software by measuring wound width reduction over time. The migration rate was calculated as a percentage of wound closure relative to the initial scratch area.

### 5.14 Flow Cytometry

Flow cytometric analysis was performed to characterize hDPSCs and to assess inflammatory marker expression and M1‐ and M2‐associated markers in BV2 microglial cells. For hDPSC characterization, the hDPSCs used for secretome collection were stained with fluorophore‐conjugated antibodies against CD73, CD90, and CD105 according to the manufacturer's instructions. For BV2 microglial cell analysis, BV2 cells were treated with 300 µm CoCl2 for 24 h, with or without hDPSC secretome treatment (50 µg/mL). Cells were harvested, washed, and stained with primary antibodies against COX‐2 and IL‐1β (Cell Signaling Technology), followed by PE‐ or Alexa 488‐conjugated secondary antibodies according to the manufacturer's instructions. To further assess M1‐ and M2‐associated microglial markers, BV2 cells were also stained with antibodies against CD86 and Arg1. CD86 was used as a representative M1‐associated marker, and Arg1 was used as a representative M2‐associated marker. Flow cytometry was performed using a FACS Calibur system (BD Biosciences). Debris was excluded using FSC/SSC gating, and singlets were selected using FSC‐A and FSC‐H. A minimum of 10 000 events per sample was acquired. The percentages of CD90/CD105 double‐positive cells and cells positive for CD73, COX‐2, IL‐1β, CD86, or Arg1 were quantified using fluorescence intensity‐based gating. The gating strategy used for BV2 flow cytometry analysis is illustrated in Figure S9.

### 5.15 Inflammatory Cytokine Measurement

Cytokine secretion by BV2 cells was measured using an arigoPLEX Mouse M1/M2 Cytokines Multiplex ELISA Kit (Arigo Biolaboratories) according to the manufacturer's instructions. BV2 cells were treated with 300 µm CoCl_2_ for 24 h, with or without the hDPSC secretome (50 µg/mL). Culture supernatants were collected, and IL‐6, TNF‐α, IL‐4, and IL‐10 were quantified on a microplate reader (BioTek Instruments) based on standard curves. Data were expressed as cytokine concentrations (pg/mL).

### 5.16 NFκB Binding Activity Assay

Cortical and hippocampal tissue nuclear proteins were isolated using a NE‐PER Nuclear and Cytoplasmic Extraction Reagents Kit (Thermo Fisher Scientific) as reported previously [48]. NFκB p65 binding activity was measured using an NFκB p65 Transcription Factor Kit (Thermo Fisher Scientific). Nuclear extracts were incubated in 96‐well plates pre‐coated with NFκB‐specific oligonucleotide probes, followed by incubation with an anti‐p65 antibody and an HRP‐conjugated secondary antibody. After washing, a chemiluminescent substrate was added, and signals were quantified on a luminometer (Thermo Fisher Scientific).

### 5.17 BrdU Injection

BrdU (Thermo Fisher Scientific) was dissolved in physiological saline and administered intraperitoneally at 50 mg/kg. Stroke was induced on Day 0. For NSC proliferation, BrdU was injected three hours before sacrifice on Days 1 and 3. For immature neuronal differentiation, BrdU was administered on Day 1, and mice were sacrificed on Day 3. For NSC survival, BrdU was injected once daily from Days 1–5, and mice were sacrificed on Day 5 or Day 28. For mature neuronal differentiation, the mice injected with BrdU on Days 1–5 were sacrificed on Day 28. Brains were processed for BrdU immunohistochemistry.

### 5.18 Image Analysis

Fluorescence images were acquired using a Zeiss LSM900 confocal microscope equipped with 10× or 20× objective lenses. Z‐stack imaging was performed for colocalization analyses. Image processing and quantitative analyses were conducted using ZEN 3.2 software (Zeiss). For quantification of TUNEL‐, cleaved caspase‐3‐, IBA‐1‐, BrdU‐, SYP‐, and PSD95‐positive cells, DHE/NeuN‐, BrdU/DCX‐, and BrdU/NeuN‐double‐positive cells, and SYP/PSD95‐double‐positive puncta, every 10th anatomically matched coronal section through the cortex, CA1, and hippocampal DG was analyzed under identical imaging conditions. TUNEL‐positive cells were defined as cells showing clear TUNEL fluorescence signals within the analyzed region. Cleaved caspase‐3‐positive cells were defined as cells showing clearly detectable cleaved caspase‐3 immunofluorescence signals above background. Non‐specific signals outside the defined regions of interest or signals not associated with identifiable cell‐like structures were excluded from quantification. Figures were assembled using Adobe Photoshop CS6.

### 5.19 LC‐MS/MS Based Proteomic Analysis

Protein extraction, digestion, LC‐MS/MS acquisition, and data processing were performed essentially as previously described in our earlier work [30]. For the hDPSC secretome proteomic analysis, three independent biological replicates of hDPSC‐conditioned media were analyzed. Serum‐free DMEM was processed in parallel as a medium control. Proteins detected in any serum‐free DMEM control replicate were excluded from the hDPSC secretome protein list, and potential contaminant proteins such as keratins were also removed. After this filtering process, proteins detected in at least two of the three independent hDPSC secretome biological replicates were retained as high‐confidence hDPSC secretome‐associated proteins for downstream analyses. For tissue and cell proteomic analyses, three biological replicates per group were analyzed for the cortex and BV2 microglial cells, and four biological replicates per group were analyzed for the hippocampus. Briefly, proteins from hDPSC‐conditioned media and control media were processed using the S‐Trap micro protocol, whereas mouse brain tissues and BV2 microglial cells were prepared using the FASP method. Digested peptides were desalted, dried, and stored at −80°C until analysis. Peptides were analyzed using a nanoLC system coupled to a Q Exactive mass spectrometer operating in a data‐dependent acquisition mode. A conventional C18 reversed‐phase gradient was applied for peptide separation. MS1 and MS2 scans were acquired using standard settings for high‐resolution DDA proteomics. Raw files were processed with Proteome Discoverer (Thermo Fisher Scientific) using the Sequest HT search engine against the UniProt human and mouse databases. Carbamidomethylation of cysteine was set as a fixed modification, and methionine oxidation and N‐terminal acetylation as variable modifications. Peptide‐ and protein‐level false‐discovery rates were controlled at <1%. Label‐free quantification was performed using unique and razor peptides with total peptide amount normalization. The mass spectrometry proteomics data (dataset identifier: PXD062727) were deposited to the ProteomeXchange Consortium via the PRIDE partner repository [87, 88].

### 5.20 Open Field Test

Locomotor activity and anxiety‐like behavior were evaluated using an open field test. Mice were individually placed at the center of a clear 40 × 40‐cm square arena (with 36 cm‐high walls) and allowed to explore freely for 20 min. Behavior was recorded using an overhead video camera. Parameters, such as total distance traveled, time spent in the center zone (e.g., the central 20 × 20 cm area), and entries into the center zone were analyzed using the ANY‐maze software (Stoelting).

### 5.21 Rotarod

Motor coordination and balance were assessed using a rotarod apparatus (B.S Techno Lab Inc., Seoul, Korea). The rod accelerated from 4 to 49 revolutions per minute over three minutes. Mice were subjected to five trials with a 30‐min (minimum) rest interval between trials. The latency to fall from the rotating rod, as well as the traveled distance and running time on the rod, were recorded using a camera.

### 5.22 Pole Test

Mice were placed head‐upward on a wooden pole (1.5 cm diameter, 43 cm height). The time taken for each mouse to descend from the top of the pole to the floor was recorded, with a maximum observation time of 60 s. Motor function was evaluated using a scoring system ranging from 1 to 15: 1 = fell before reaching the top; 2 = fell from the top in <10 s; 3 = fell in <20 s; 4 = fell in <30 s; 5 = fell in <40 s; 6 = fell in <50 s; 7 = fell in <60 s; 8 = stayed on the pole for 60 s; 9 = climbed down to the lower half of the pole; 10 = climbed down to the ground in >50 s; 11 = climbed down in >40 s; 12 = climbed down in >30 s; 13 = climbed down in >20 s; 14 = climbed down in >10 s; 15 = climbed down in <10 s.

### 5.23 Negative Geotaxis Test

Postural reflexes and motor coordination were evaluated using the negative geotaxis test. Mice were placed facing downward on a flat surface inclined at a 60° angle. The time taken for each mouse to turn 180° (to orient itself facing upward) was recorded, with a maximum cut‐off time of 120 s per trial. The test was performed twice for each mouse, with a 30‐min inter‐trial interval.

### 5.24 Barnes Maze

Spatial learning and memory were assessed using a Barnes maze, which consisted of a circular platform (diameter: 91 cm) with 20 peripheral holes, one leading to a fixed escape tunnel (25 × 6 × 5 cm). Mice underwent training for four consecutive days, with three trials daily. In each trial, a mouse was placed under a PVC start ring in the center of the platform, and the trial began upon removal of the ring. Mice that did not enter the escape tunnel within 60 s were gently guided to it. Trials were terminated once the mouse entered the tunnel or after 180 s (maximum). After training (e.g., 24 h after the last training session), a 180‐s probe trial was conducted without the escape tunnel. Behavioral parameters, including latency to first reach the target location, traveled distance, and time spent in the target quadrant, were recorded and analyzed using a video camera and the ANY‐maze software.

### 5.25 Novel Object Location Test

Recognition memory was assessed using a novel object location test in an open field box. Initially, during the familiarization phase, mice were placed in the arena for eight minutes and exposed to two identical objects. After a three‐hour delay, mice were returned to the same arena for an eight‐minute test session (Phase 1) after moving one of the objects to a novel location. The next day, mice were exposed to the arena for eight minutes (Phase 2) after moving one object to a previously unoccupied location. The novel location for Phase 1 was in the corner opposite the object's original position during familiarization, and for Phase 2, it was in a corner not previously used for any object. All object locations were counterbalanced across the animals, and objects were placed 3 cm from the walls. The time spent exploring each object was recorded using the ANY‐maze software. A preference index was calculated as follows: [(time exploring the novel‐located object)/(total exploration time for both objects)] × 100%.

### 5.26 Cross‐Maze

Short‐term spatial working memory was assessed by recording spontaneous alternation behavior in a four‐arm cross‐maze. The maze had four arms (labeled A, B, C, and D) extending from a central platform. Each mouse was initially placed at the end of arm A and allowed to freely explore the maze for 10 min. Arm entry was indicated by the four paws entering an arm. The sequence of arm entries was recorded. Spontaneous alternation was defined as consecutive entries into four different arms in overlapping sets. The percentage of spontaneous alternations was calculated as follows: [number of alternations/(total number of arm entries – 3)] × 100%.

### 5.27 Contextual Fear Conditioning Test

Contextual fear memory was assessed using a fear conditioning paradigm in a square (20 cm × 20 cm × 50 cm) conditioning chamber with a stainless‐steel grid floor connected to an electric shock generator. The test involved two phases. On the training day (Day 1), mice were placed in the chamber and allowed to explore for two minutes, after which a single electric foot shock (0.5 mA) was delivered for two seconds. Twenty‐four hours later (Day 2, context test), mice were returned to the same chamber for three minutes without the foot shock. Freezing behavior, the complete absence of movement except for respiration, was recorded during both phases using the ANY‐maze system.

### 5.28 Statistical Analyses

Data are presented as mean ± standard error of the mean (SEM). Differences between groups were compared using a one‐way analysis of variance (ANOVA) followed by Tukey's post hoc test for multiple comparisons or unpaired *t*‐tests for two‐group comparisons, using GraphPad Prism 6. Statistical significance was defined as follows: *, **, *** and **** indicate *p* < 0.05, *p* < 0.01, *p* < 0.001, and *p* < 0.0001, respectively, with *p* < 0.05 considered statistically significant. Statistical evaluation of the LC‐MS/MS data was conducted based on protein abundance levels. Each sample group was composed of three biological replicates, except for the hippocampus samples, which included four biological replicates. For proteomic data analysis, ANOVA followed by Tukey's post hoc test was applied to assess differences in protein abundance ratios among groups. Proteins showing a *p*‐value below 0.05 and a fold‐change greater than or equal to 1.5 were classified as DEPs. Functional enrichment analyses, including GO. KEGG, and Reactome pathway, were conducted using the online DAVID tool (https://david.ncifcrf.gov/) [89, 90]. PPI networks were constructed using the online STRING database (https://string‐db.org/) [91], where edges were drawn based on confidence scores with a minimum interaction score threshold of 0.15. In addition, PCA, pathway mapping, and visualization (heatmaps, chord diagrams) were performed in R 4.2.2 using standard packages.

## Author Contributions

K.‐J.S. and S.P. conceived the study and designed the experiments; K.‐J.S. supervised the research; K.‐J.S., S.P., S.‐W.B., D.K., and J.H.L. conducted the main experiments; K.‐J.S. and S.P. performed data curation and formal analysis; S.P. contributed to validation and resources; K.‐J.S. and S.P. wrote the original draft; K.‐J.S., S.P., W.‐S.C., Z.‐Y.P., J.‐Y.J., and W.‐J.K. reviewed and edited the manuscript; K.‐J.S., W.‐S.C., Z.‐Y.P., J.‐Y.J., and W.‐J.K. acquired funding. All authors reviewed and approved the final manuscript.

## Conflicts of Interest

The authors declare no conflict of interest.

## Supporting information




**Supporting File 1**: advs76717‐sup‐0001‐SuppMat.docx.

**Supporting File 2**: advs76717‐sup‐0002‐SuppTable.docx.

## Data Availability

The data underlying this study are available from the corresponding authors upon reasonable request. Mass spectrometry data are available on ProteomeXchange Consortium with dataset identifier PXD062727. Reviewer can access the data using Project accession: PXD062727, Token: DubPujtki3BN or username: reviewer_pxd062727@ebi.ac.uk, password: xJjGwtmcyYOj.
