# Supplementary material for: Human Dental Pulp Stem Cell Secretome Restores Ischemic Stroke–Impaired Motor and Cognitive Functions by Reprogramming Redox and Inflammatory Signaling
Source: Adv Sci (Weinh). 2026 Jul 23:e76717. Online ahead of print. doi: 10.1002/advs.76717 (PMC13393272; doi:10.1002/advs.76717)

**Supplementary Information**

**Human dental pulp stem cell secretome restores ischemic stroke–impaired motor and cognitive functions by reprogramming redox and inflammatory signaling**

**Abbreviations**

CM: Conditioned medium; MSC: Mesenchymal stem cell; hDPSC: Human dental pulp stem cell; HIF: Hypoxia-inducible factor; ROS: Reactive oxygen species; ETC: Electron transport chain; MitoSOX Red: Mitochondrial Superoxide Indicator; DCFDA: 2’,7’-dichlorodihydrofluorescein diacetate staining; SOD: Superoxide dismutase; 7-AAD: 7-Amino-Actinomycin; ICC: Immunocytochemistry; S-Trap: Suspension-Trapping method; FASP: Filter Aided Sample Preparation; GO: Gene Ontology; KEGG: Kyoto Encyclopedia of Genes and Genomes; CC: Cellular component; MF: Molecular function; BP: Biological process; PPI: Protein-protein interaction; PCA: Principal component analysis; DEP: Differentially expressed protein; NAC: N-acetylcysteine; B-cell lymphoma-2: Bcl-2; B-cell lymphoma-extra-large: Bcl-XL; Toll-like receptor 4: TLR4; Tumor necrosis factor-alpha: TNF-α; Interleukin-1 beta: IL-1β; Interleukin-6: IL-6; Cyclooxygenase-2: COX-2; Inducible nitric oxide synthase: iNOS; NF-H: neurofilament heavy chain


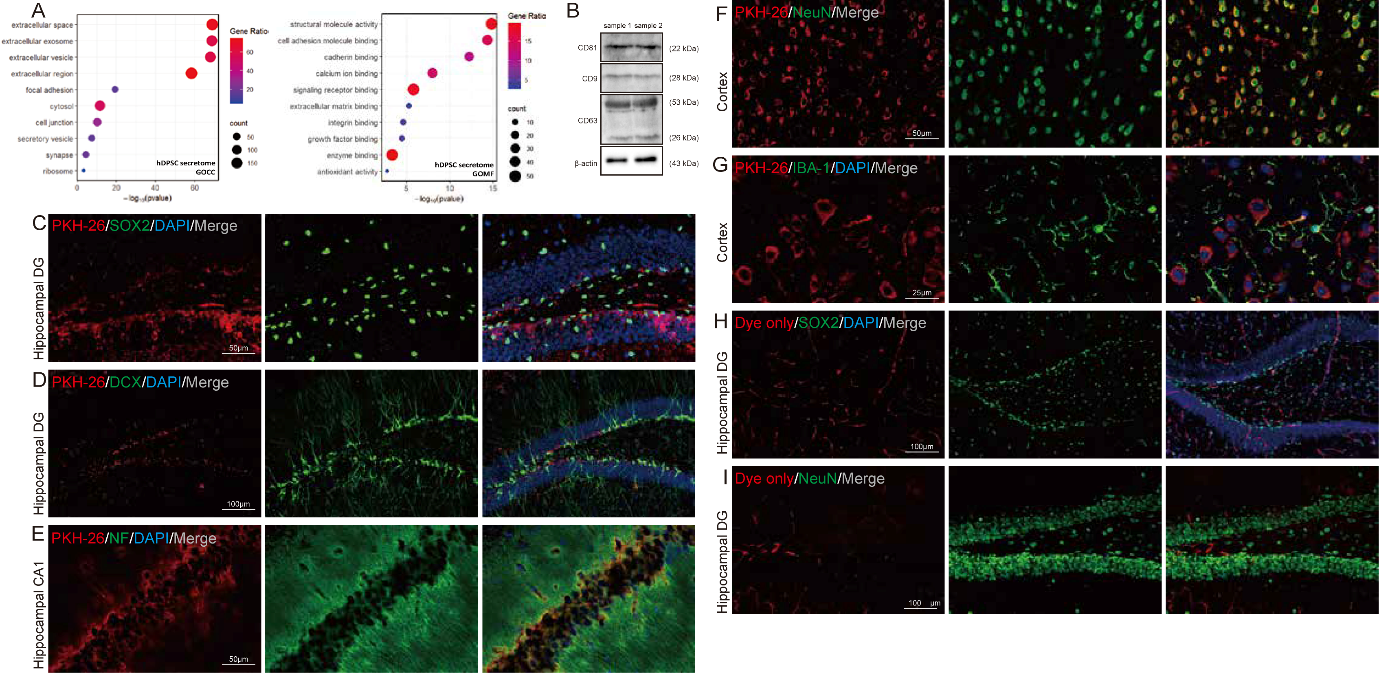


Figure S1. Brain distribution of PKH26-labeled hDPSC secretome-derived exosome-enriched particles and proteomic features of the hDPSC secretome. (A) Gene Ontology enrichment analyses of the proteins that were consistently identified in at least two biological replicates of the hDPSC secretome. GOCC terms revealed enrichment in extracellular exosome, vesicle, and synapse-related proteins. GOMF terms indicated enrichment in structural molecule activity, enzyme binding, growth factor binding, and antioxidant activity. The displayed GO terms were significantly enriched (*p* < 0.05). (B) Representative Western blot images showing the expression of the extracellular vesicle markers CD81, CD9, and CD63 in exosome-enriched particles derived from the hDPSC secretome. (C~G) Representative confocal images of the biodistribution of systemically-administered PKH26-labeled secretome-derived particles. PKH26-labeled particles (red) were detected in SOX2-positive NSCs (C) and DCX-positive immature neurons (D) in the hippocampal DG, neurofilament (NF)-positive fibers in the hippocampal CA1 region (E), NeuN-positive mature neurons in the cortex (F), and IBA-1-positive microglia in the cortex. (G) Representative confocal images of dye-only control sections processed without exosome-enriched fractions(H,I). Nuclei were stained with DAPI (blue). Scale bars: 50–100 µm.

**
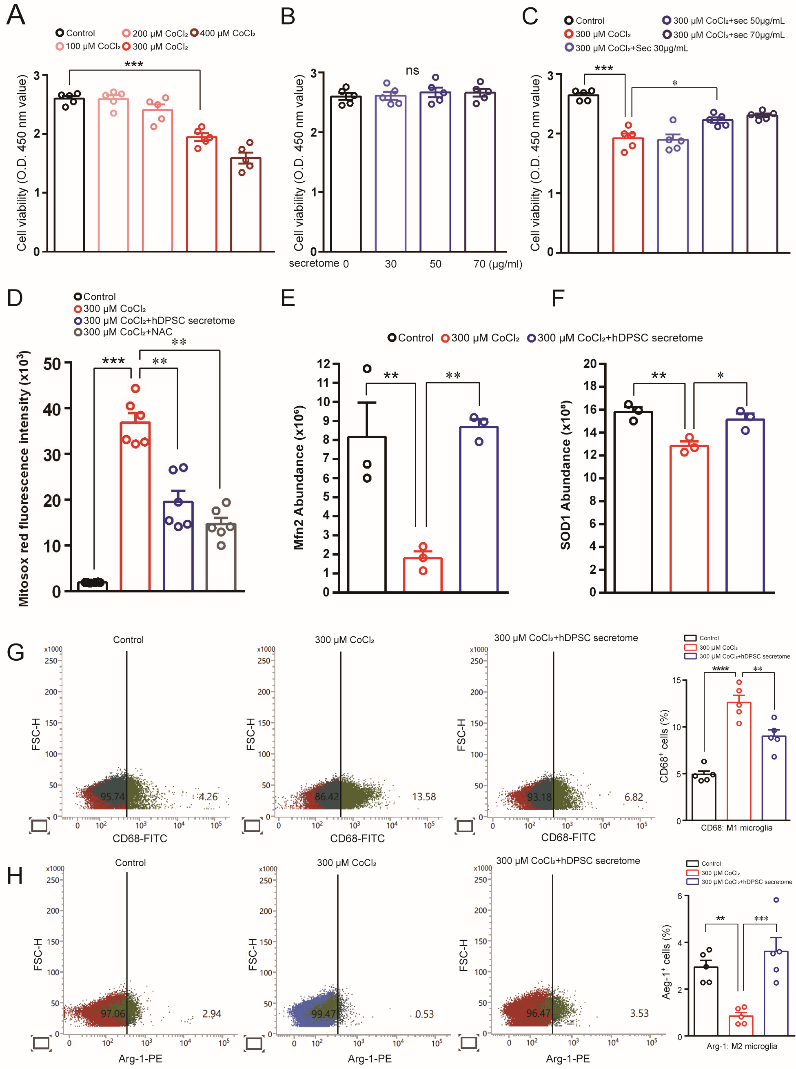
**

Figure S2. Mitochondrial ROS levels, proteomic changes, and M1/M2-associated marker expression in BV2 cells. (A–C) Cell viability was determined using the EZ-Cytox assay. (A) BV2 cells were treated with CoCl₂ at 0, 100, 200, 300, and 400 µM. (B) BV2 cells were treated with hDPSC secretome at 0, 30, 50, and 70 µg/mL to evaluate its dose-dependent effect. (C) BV2 cells were treated with 300 µM CoCl₂ with or without hDPSC secretome treatment at 30, 50, or 70 µg/mL to assess its rescue effect. (D) MitoSOX red fluorescence intensity in BV2 cells indicates mitochondrial ROS levels in different treatment groups. (E,F) Relative Mfn2 (E) and SOD1 (F) abundance in BV2 cells based on proteomic analysis. (G,H) Representative flow cytometry plots and quantification of CD86-positive cells as an M1-associated marker (G) and Arg1-positive cells as an M2-associated marker (H) in BV2 cells treated with 300 µM CoCl₂ with or without hDPSC secretome treatment. Open circles represent individual biological replicates. Data are presented as mean ± SEM. Statistical comparisons were performed using one-way ANOVA with Tukey’s post hoc test (*p < 0.05, **p < 0.01, ***p < 0.001).


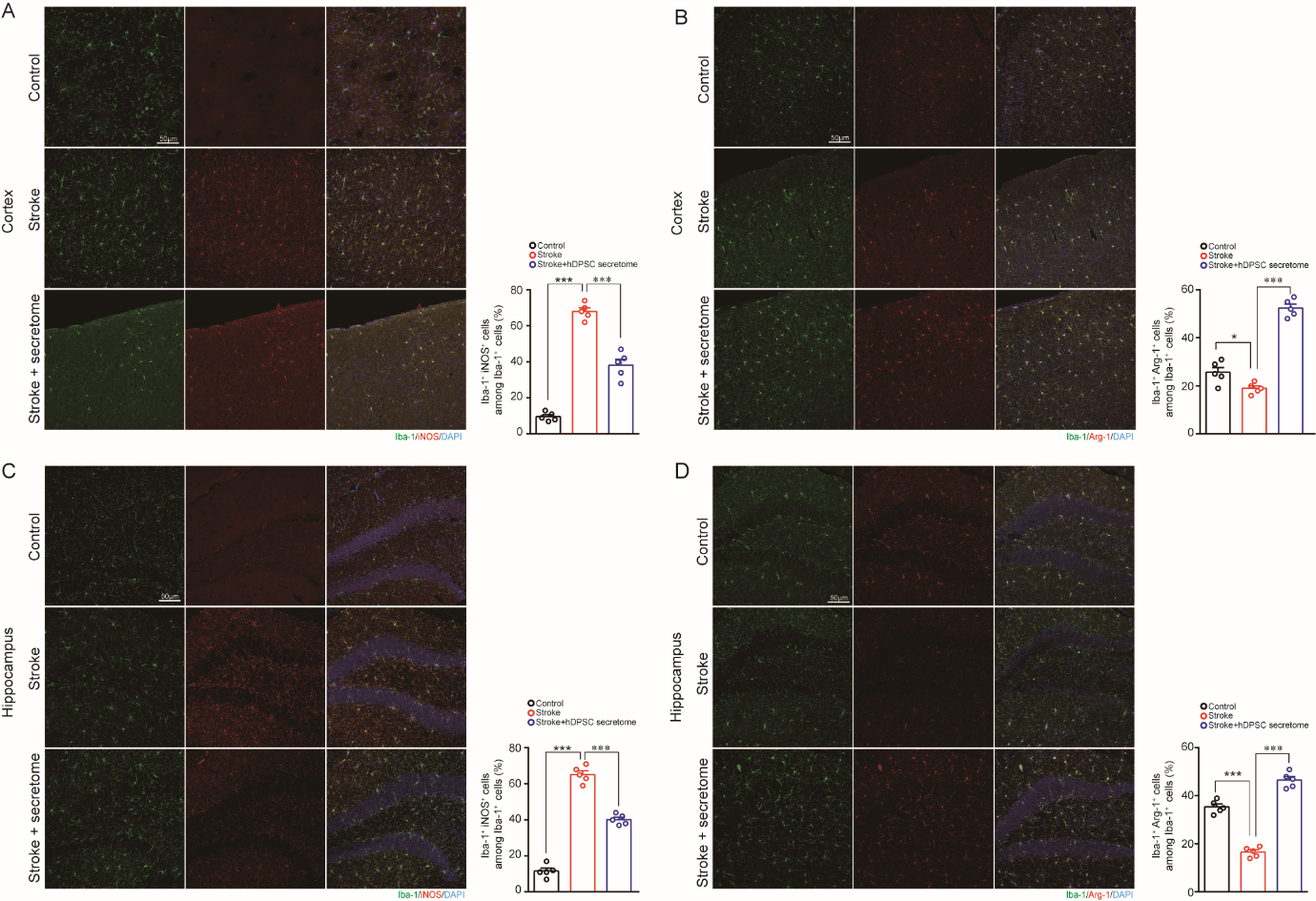


Figure S3. hDPSC secretome regulates microglial phenotype-associated markers after ischemic stroke. (A,C) Representative immunofluorescence images and quantification of Iba-1/iNOS-double-positive cells in the cortex (A) and hippocampus (C). (B,D) Representative immunofluorescence images and quantification of Iba-1/Arg-1-double-positive cells in the cortex (B) and hippocampus (D). The percentage of double-positive cells was calculated among total Iba-1-positive cells in the same region. Five ROIs were analyzed per animal, and five animals were included in each group. Nuclei were counterstained with DAPI. Open circles represent individual biological replicates. Data are presented as mean ± SEM. Statistical comparisons were performed using one-way ANOVA with Tukey’s post hoc test (*p < 0.05, ***p < 0.001). Scale bar = 50 μm.


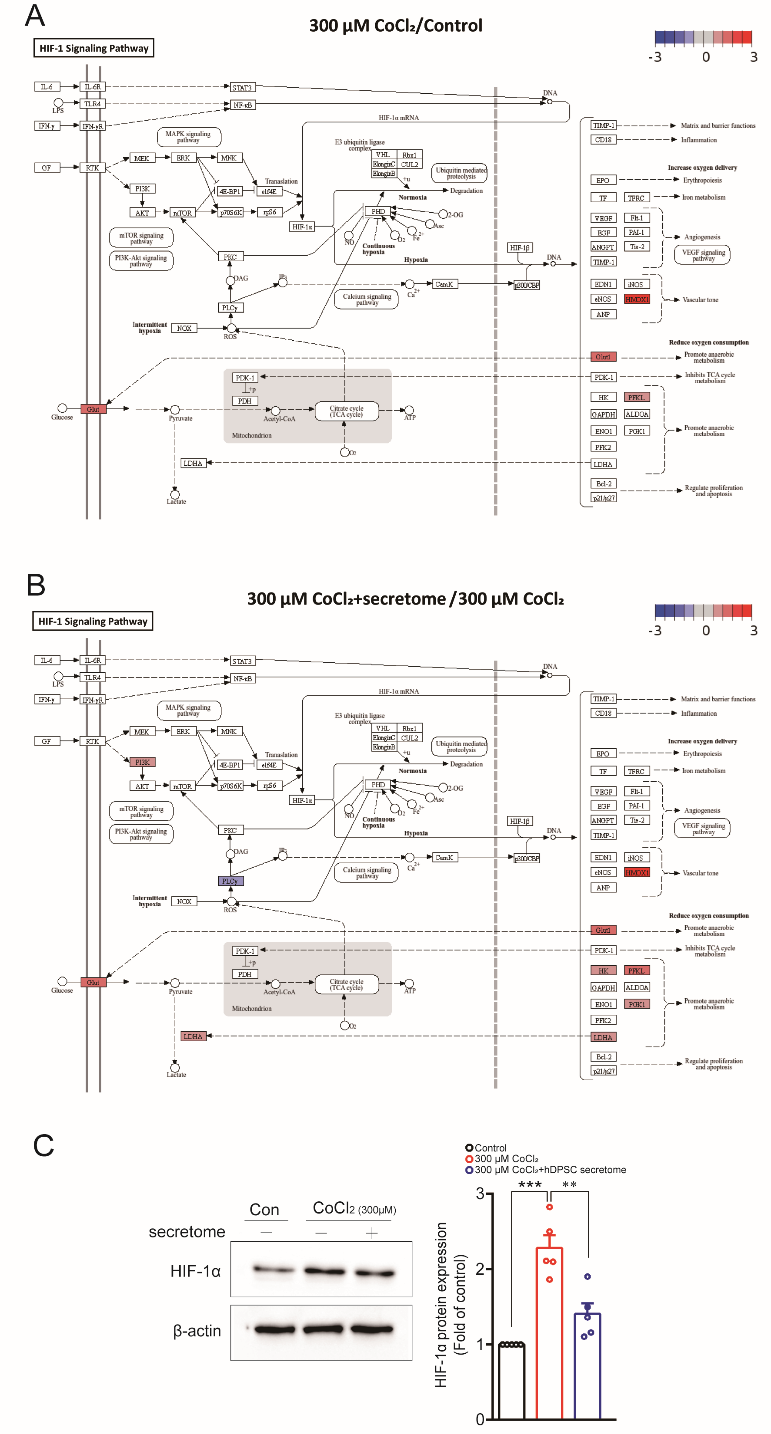


Figure S4. KEGG pathway analysis of HIF-1 signaling in BV-2 microglial cells. (A,B) KEGG pathway diagrams for “HIF-1 signaling pathway” showing mapped DEPs from the BV2 cells in hypoxia/control (A) and hypoxia with the hDPSC secretome/hypoxia (B). Node colors represent log2-transformed fold changes in protein expression. (C) Representative Western blot images and HIF-1α quantification. Protein levels were normalized to β-actin (loading control). Open circles represent individual biological replicates. Data are presented as mean ± SEM. Statistical comparisons were performed using one-way ANOVA with Tukey’s post hoc test (*p < 0.05, ***p < 0.001).


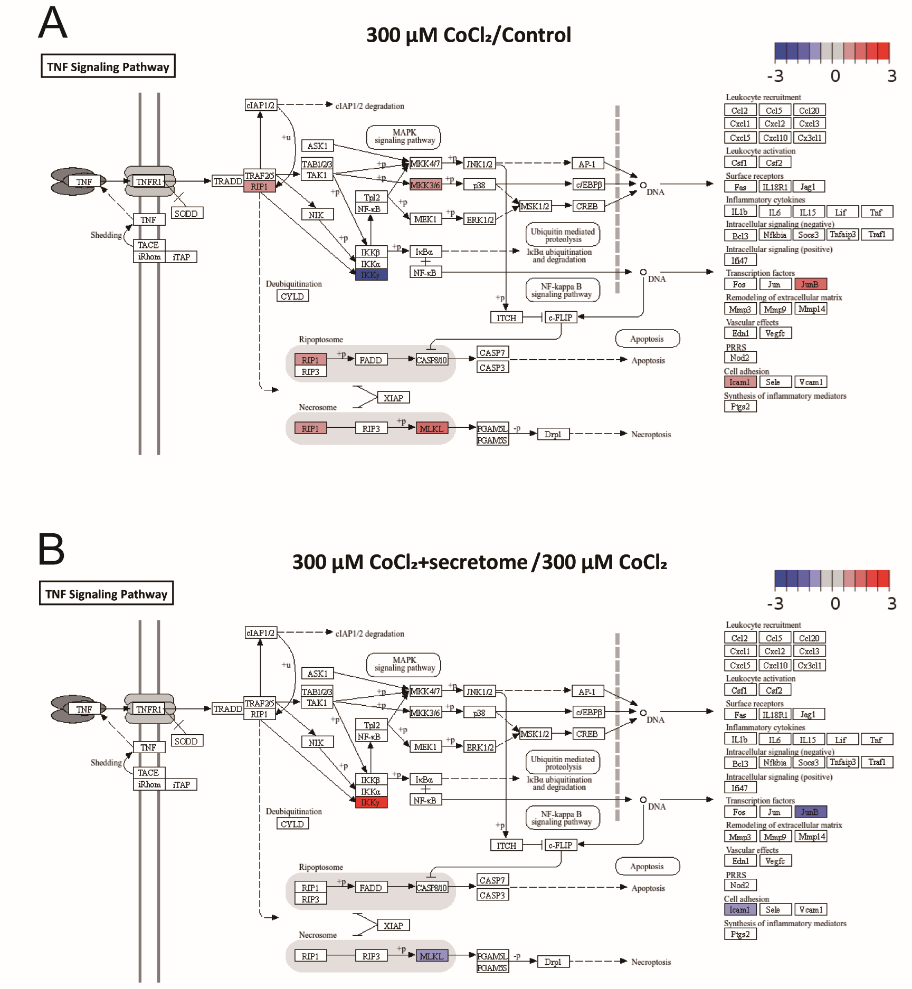


Figure S5. KEGG pathway analysis of TNF signaling in BV2 cells treated with 300 µM CoCl_2_. (A,B) KEGG pathway diagrams for “TNF signaling pathway” showing mapped DEPs from the BV2 cells in hypoxia/control (A) and hypoxia with the hDPSC secretome/hypoxia (B). Node colors represent log2-transformed fold changes in protein expression.

**
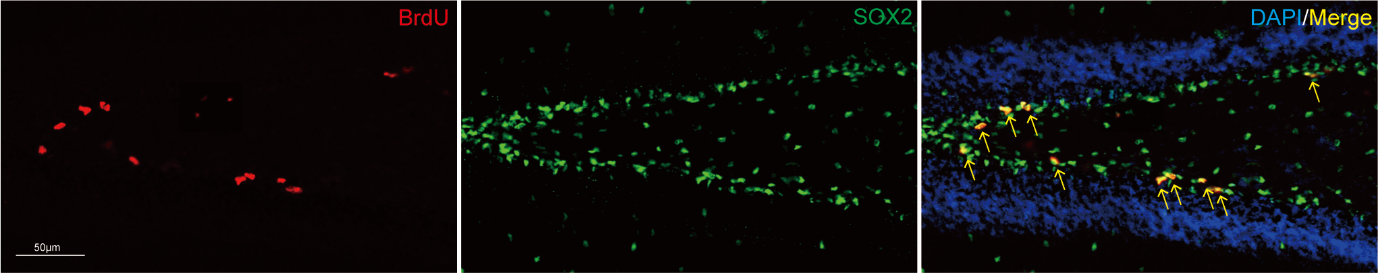
**

Figure S6. Identification of type 2a NSCs in the hippocampal DG. Representative immunofluorescence images of the hippocampal DG showing BrdU-positive cells (red), SOX2-positive cells (green), and merged images with DAPI nuclear staining (blue). Co-localization of BrdU and SOX2 signals (yellow arrows) indicates that BrdU-incorporating cells correspond to SOX2-expressing type 2a NSCs. Scale bar: 50 µm.


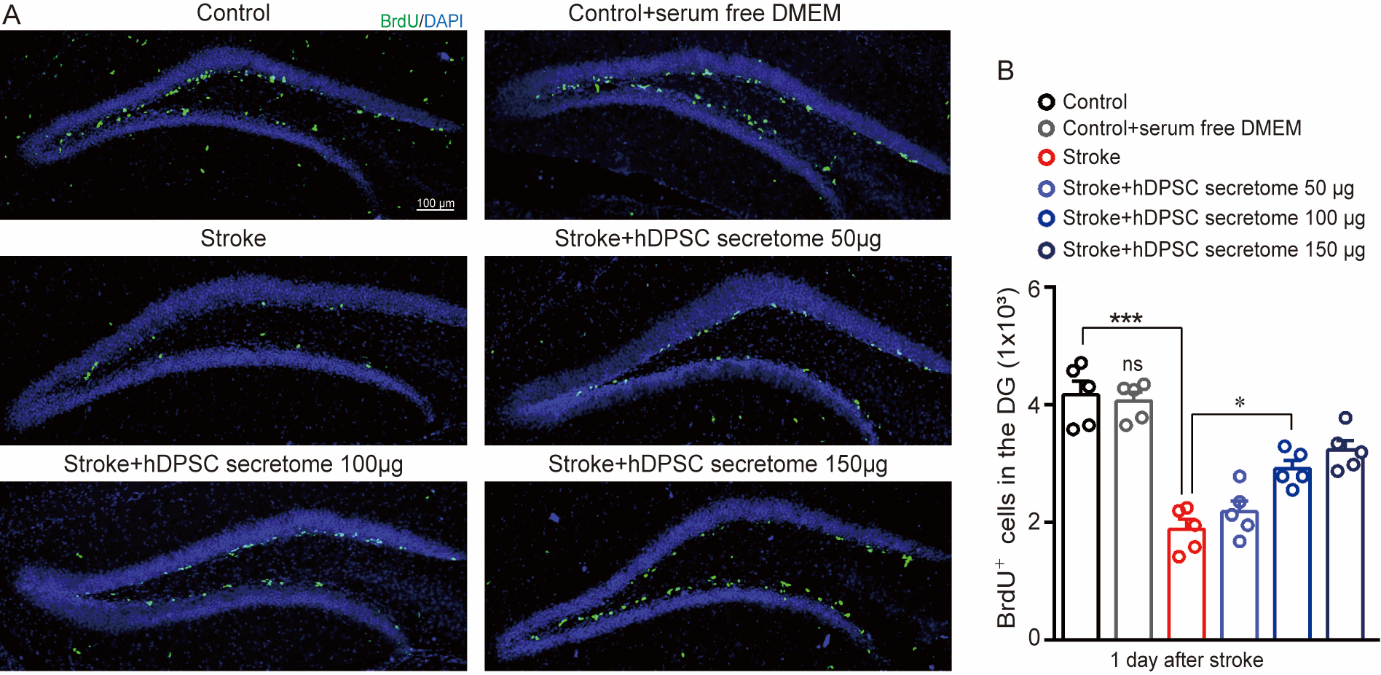


Figure S7. The hDPSC secretome attenuates ischemic stroke-induced reduction in NSC proliferation in the hippocampal DG. (A) Representative BrdU immunofluorescence images of the hippocampal DG in the control, serum-free DMEM-treated control, stroke, and hDPSC secretome-treated stroke groups (50, 100, and 150 μg) at 1 day after stroke. BrdU-positive cells are shown in green, and nuclei are stained with DAPI (blue). (B) Quantification of BrdU-positive cells in the DG at 1 day after stroke. Open circles represent individual biological replicates. Data are presented as mean ± SEM. Statistical comparisons were performed using one-way ANOVA with Tukey’s post hoc test (*p < 0.05, ***p < 0.001). Scale bar: 100 μm.


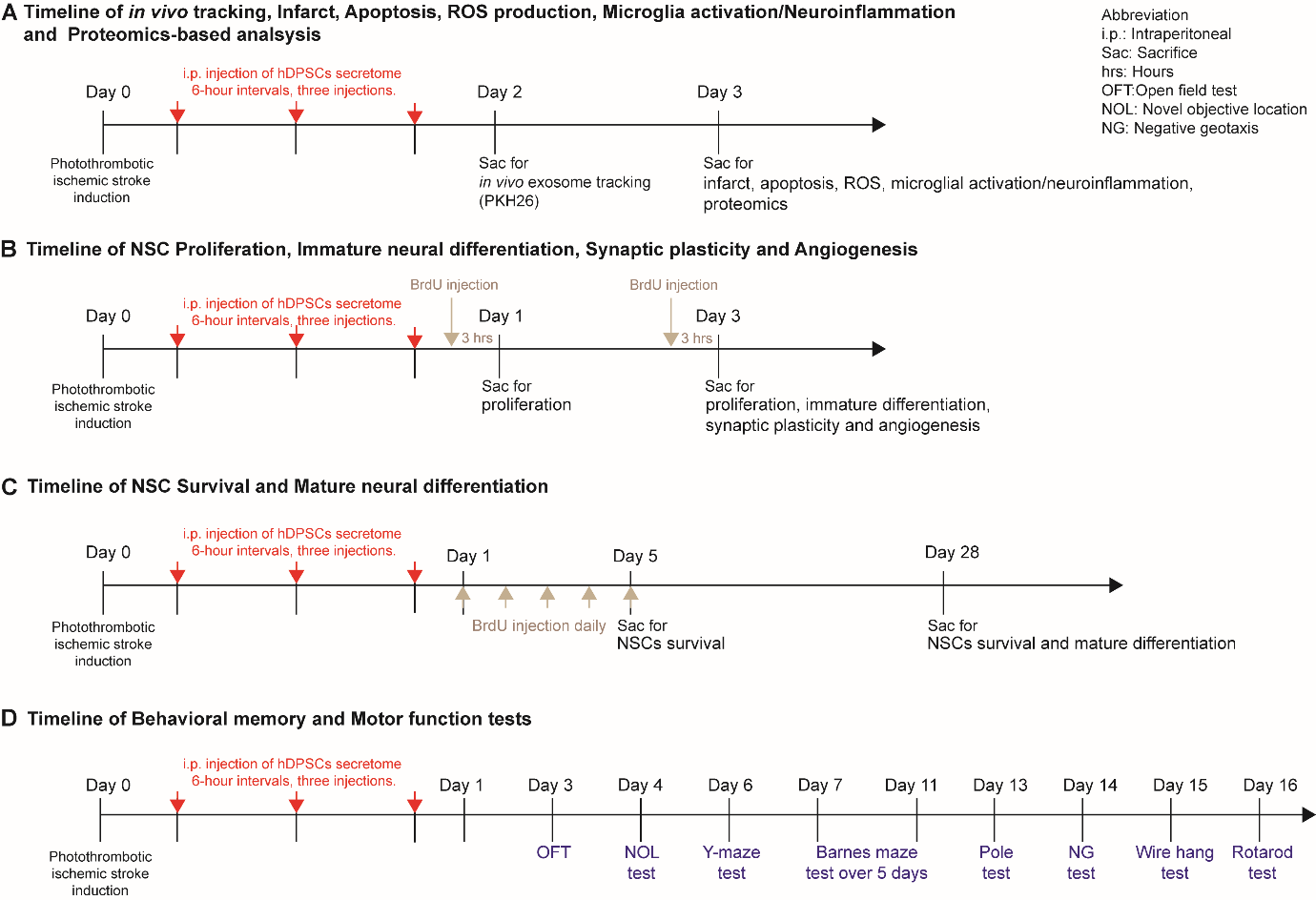


Figure S8. Experimental timelines for the ischemic stroke model and hDPSC secretome administration. (A) The hDPSC secretome was administered intraperitoneally (thrice) at six-hour intervals after stroke induction. Timeline of *in* *vivo* tracking, infarct size measurement, apoptosis, ROS production, microglia activation, neuroinflammation, and proteomics-based analyses up to three days after inducing photothrombotic ischemic stroke. (B) Timeline of NSC proliferation, immature neural differentiation, synaptic plasticity, and angiogenesis, including BrdU injections one and three days after stroke. (C) NSC survival timeline and analyses of mature neural differentiation five and 28 days after stroke. (D) Timeline of behavioral test schedule to evaluate motor and cognitive functions. Motor function tests: pole test, negative geotaxis (NG), wire hang test, and rotarod test. Cognitive function tests: open field test (OFT), novel object location (NOL) test, Y-maze test, and Barnes maze test. Contextual fear conditioning (CFC) test.

**
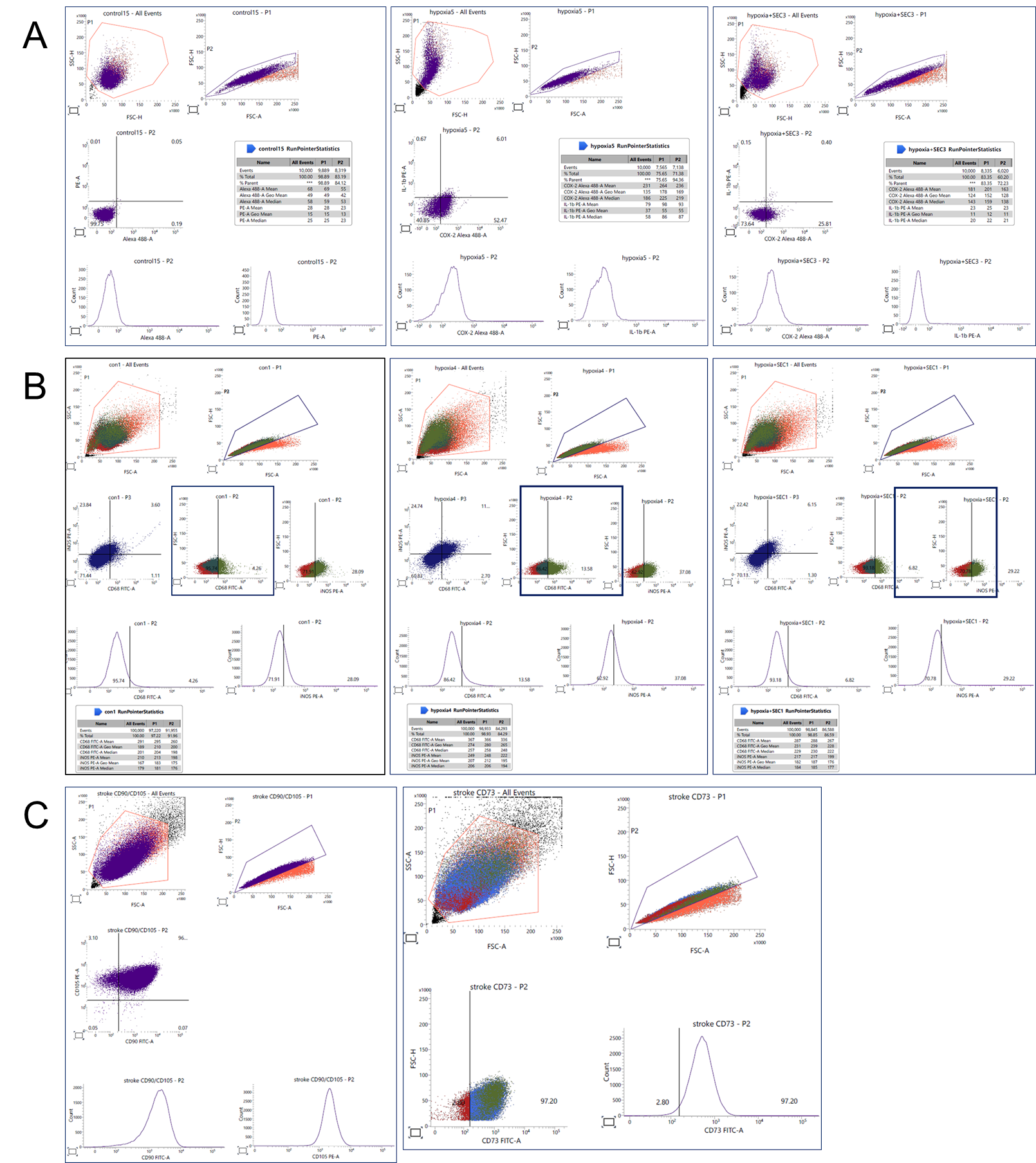
**

Figure S9. Gating strategies for flow cytometry analyses. Representative gating strategies showing debris exclusion using FSC/SSC, singlet selection using FSC-A/FSC-H, and marker-positive population analysis based on fluorescence intensity. (A-C) For BV2 microglial cell analysis, COX-2- and IL-1β-positive cells were quantified using Alexa Fluor 488 and PE channels, respectively (A). CD86-positive and Arg1-positive populations were also analyzed to assess M1- and M2-associated microglial markers (B). For hDPSC characterization, CD90/CD105 double-positive and CD73-positive populations were analyzed (C). A minimum of 10,000 events was acquired for each sample.

**
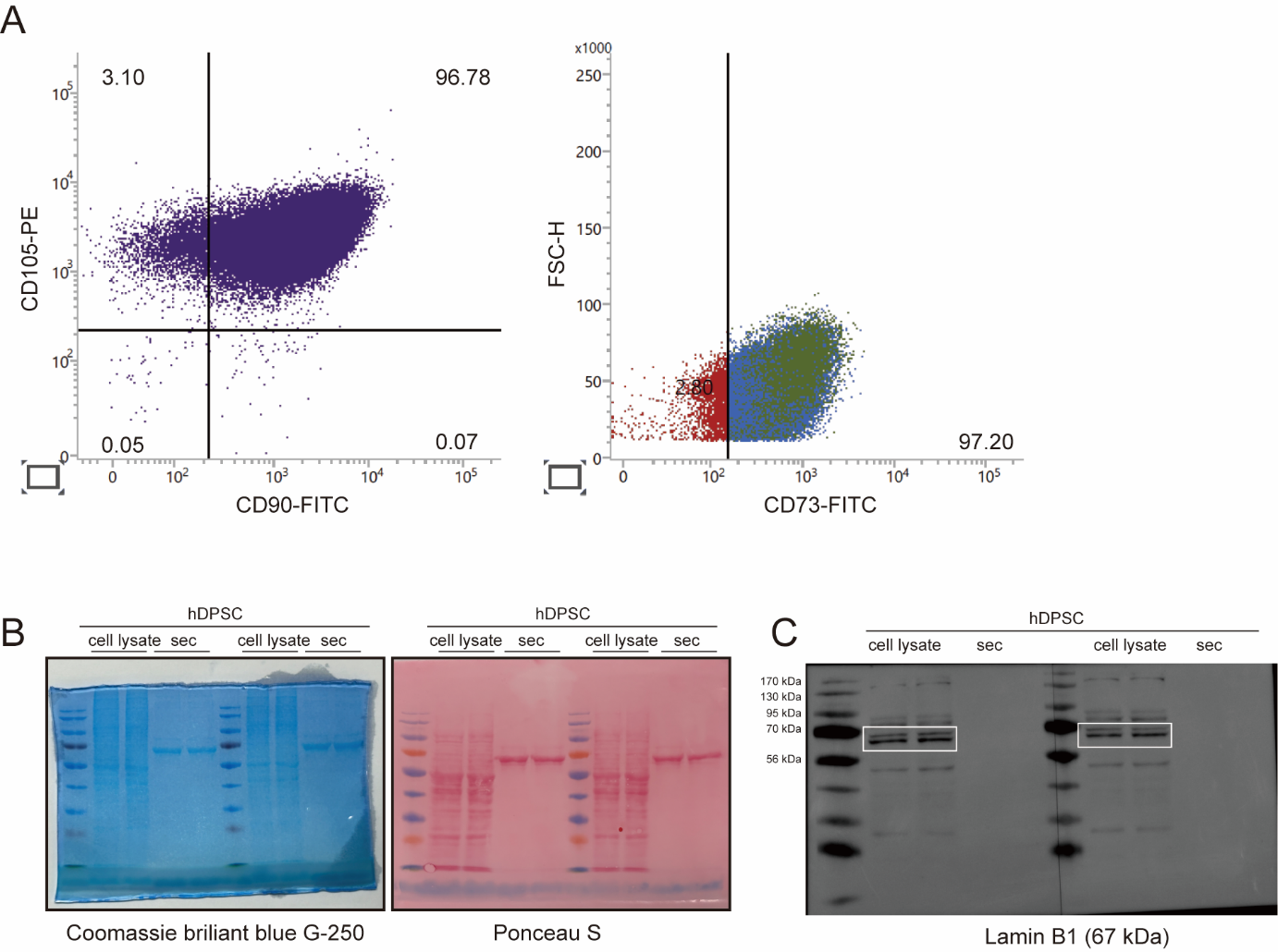
**

Figure S10. Characterization of hDPSCs and hDPSC secretome. (A) Flow cytometric analysis of MSC-associated surface markers in hDPSCs. hDPSCs were CD90/CD105 double-positive and CD73-positive. (B) Coomassie brilliant blue G-250 and Ponceau S staining of hDPSC cell lysate and hDPSC secretome samples. (C) Western blot analysis of Lamin B1 in hDPSC cell lysate and hDPSC secretome samples. hDPSC cell lysate was used as a positive control for Lamin B1 detection. sec: secretome.

**Source Data**

The original Full-length blots of the Fig. 1P


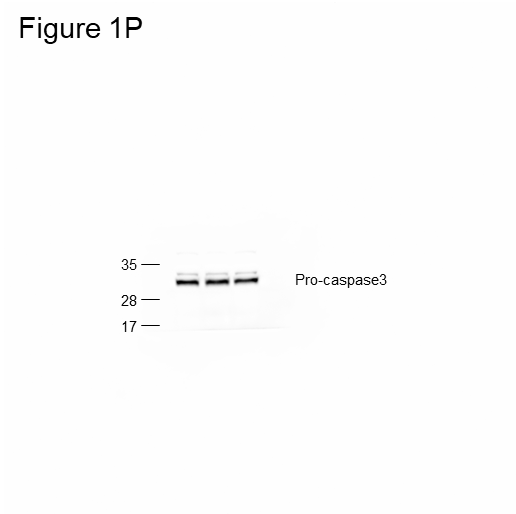

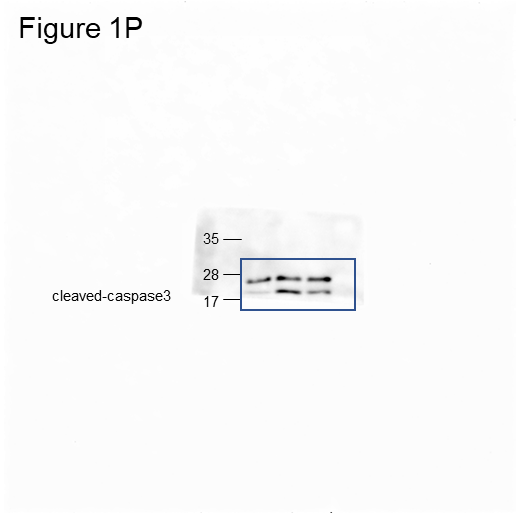

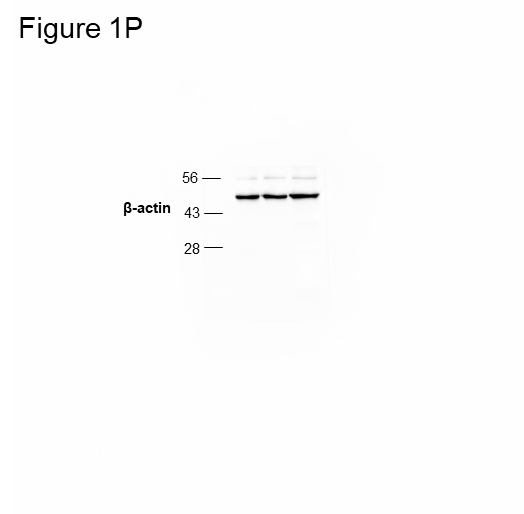


The original Full-length blots of the Fig. 1Q


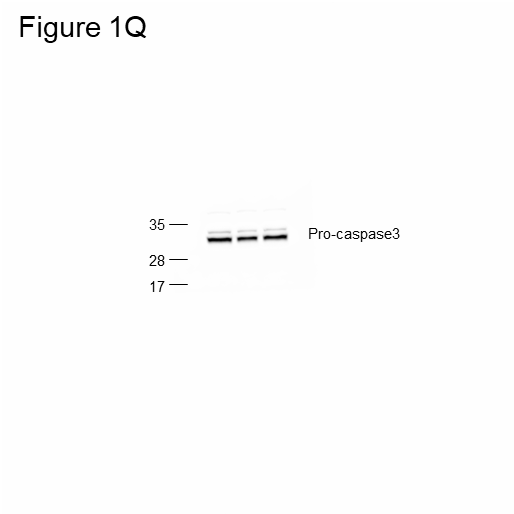

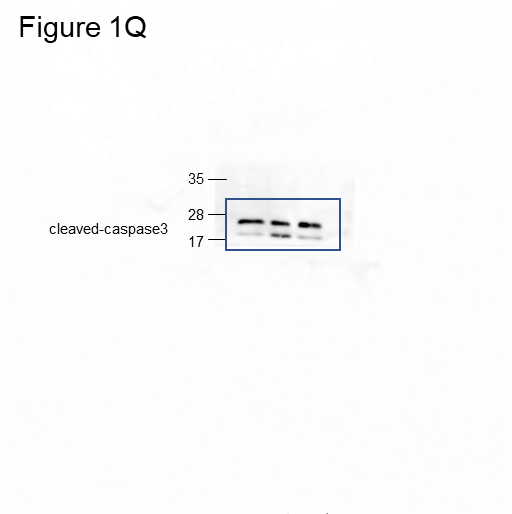

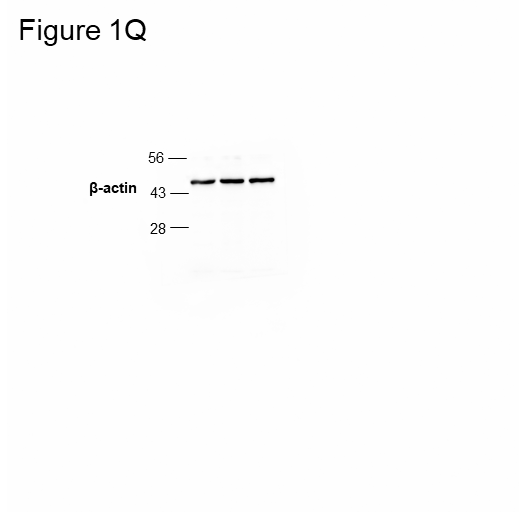


The original Full-length blots of the Fig. 1R


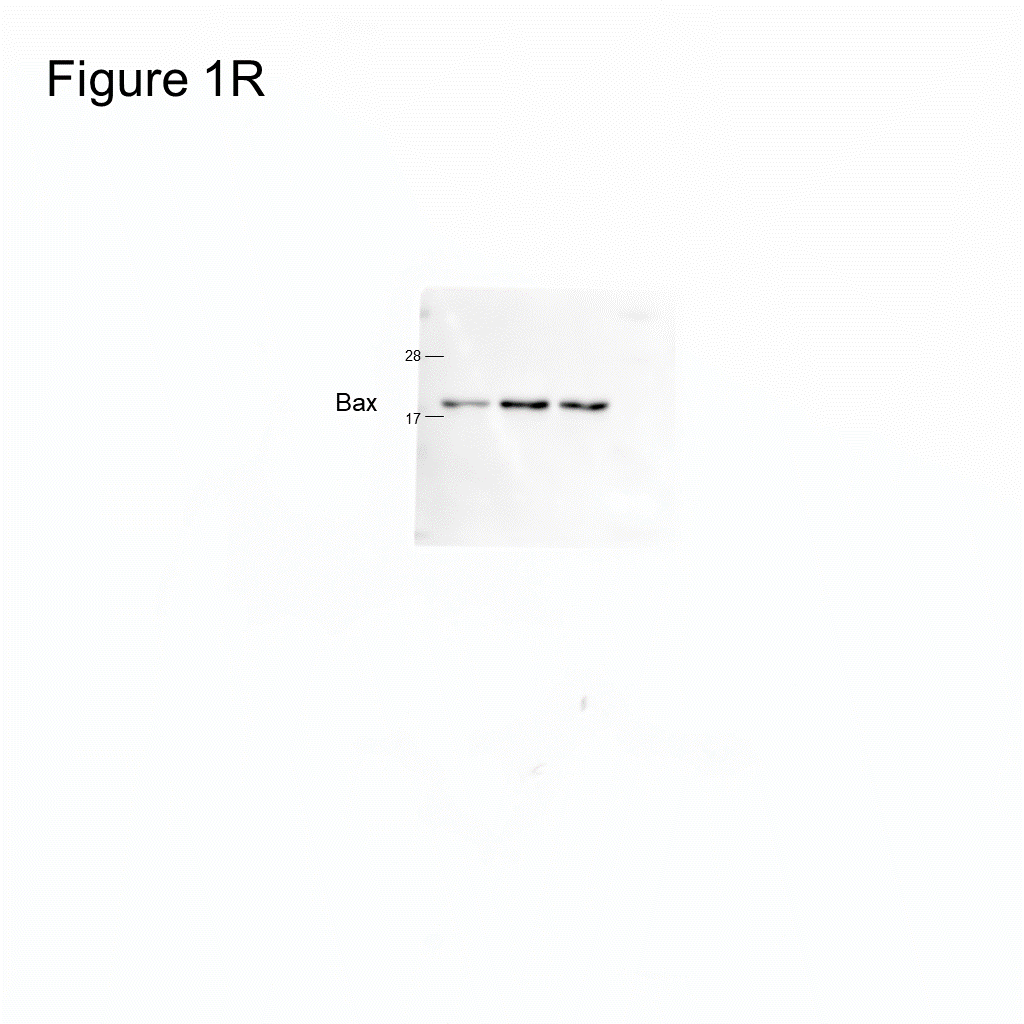

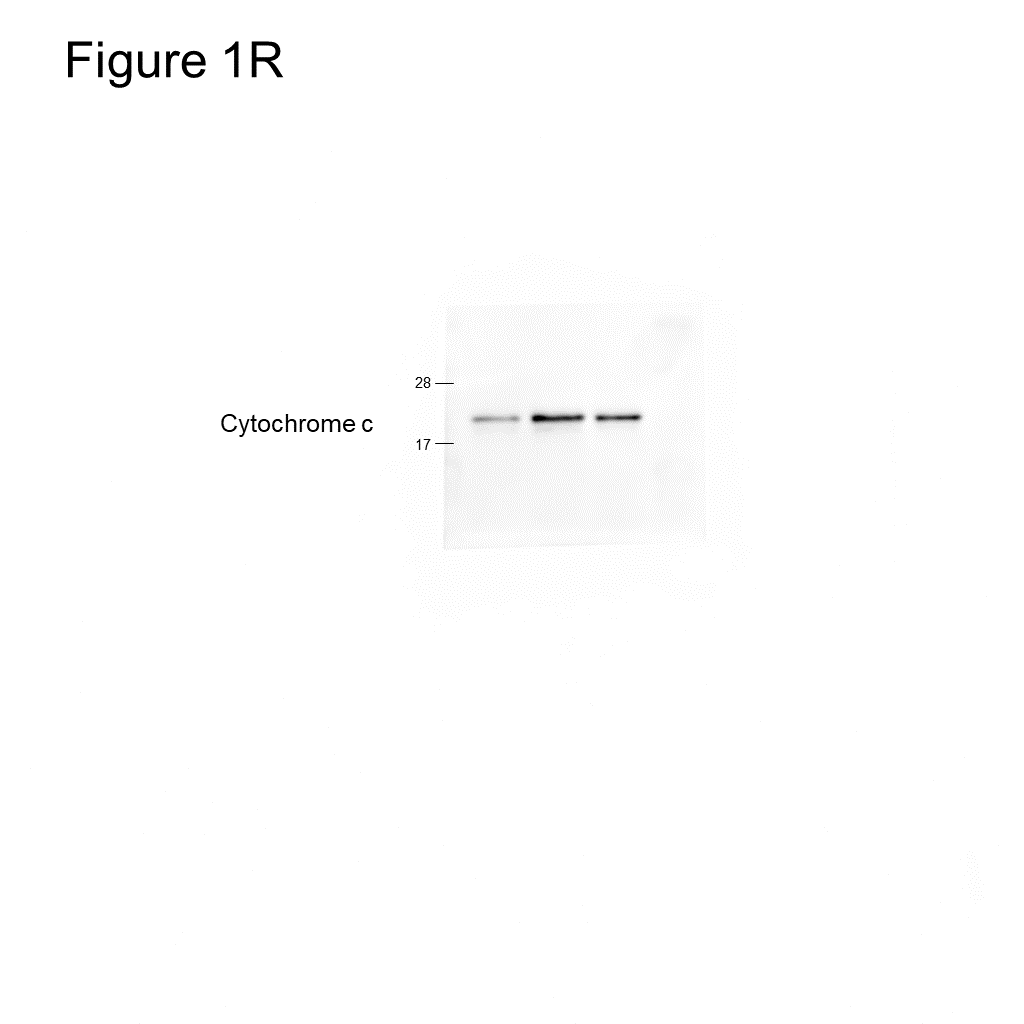

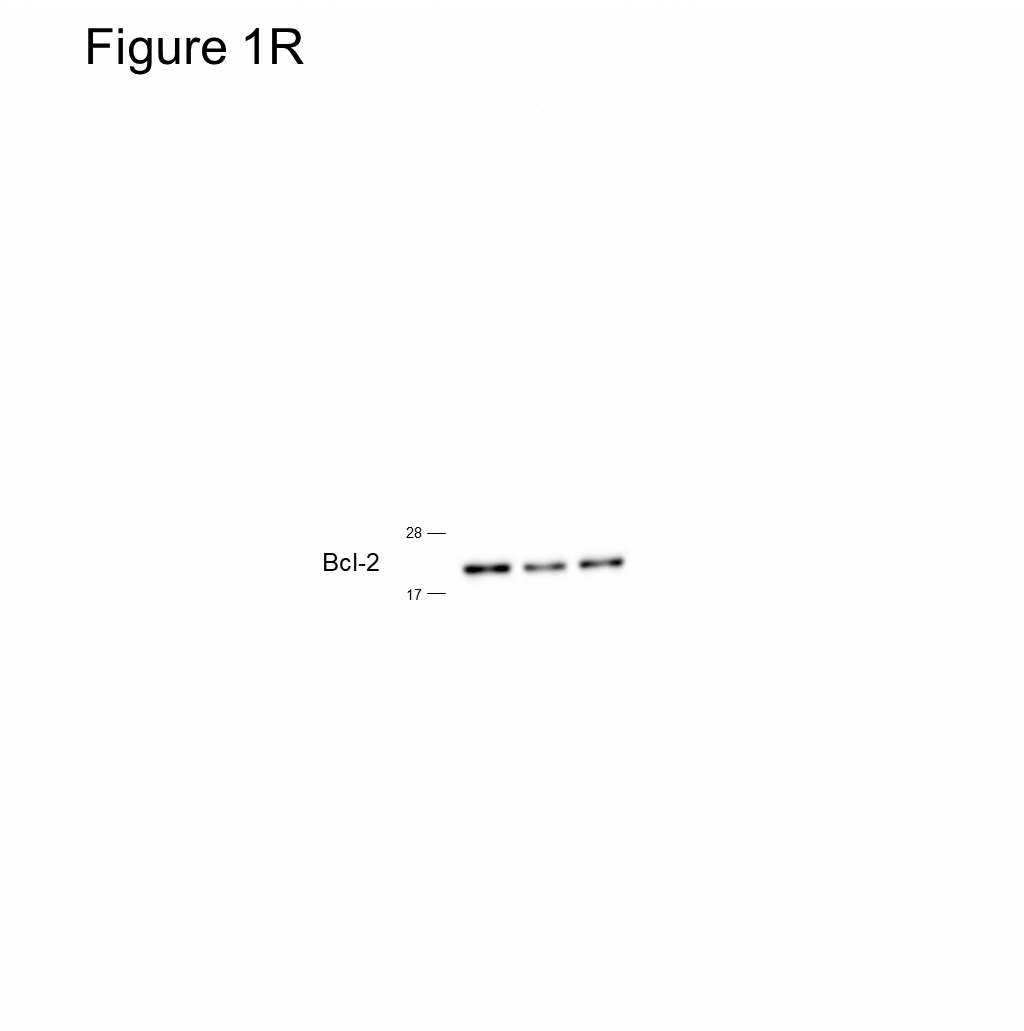


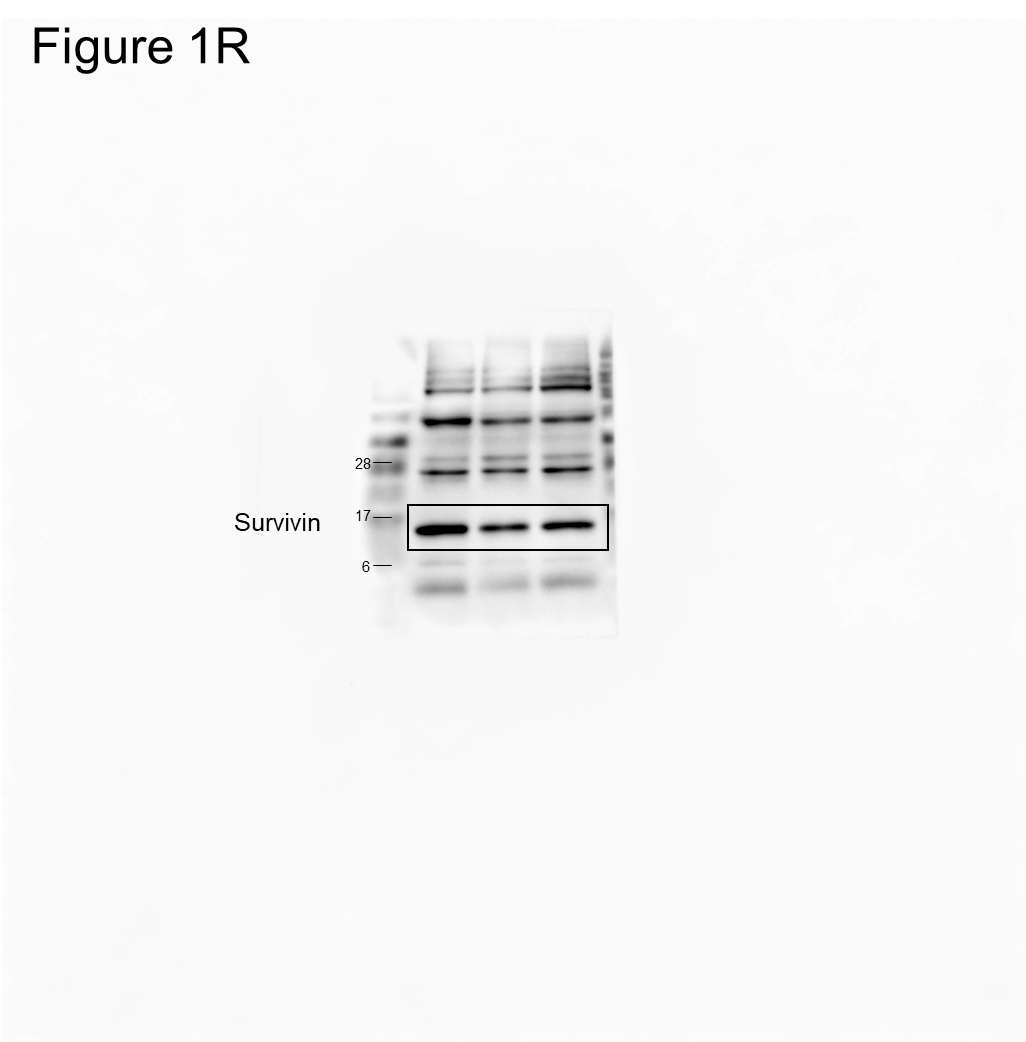

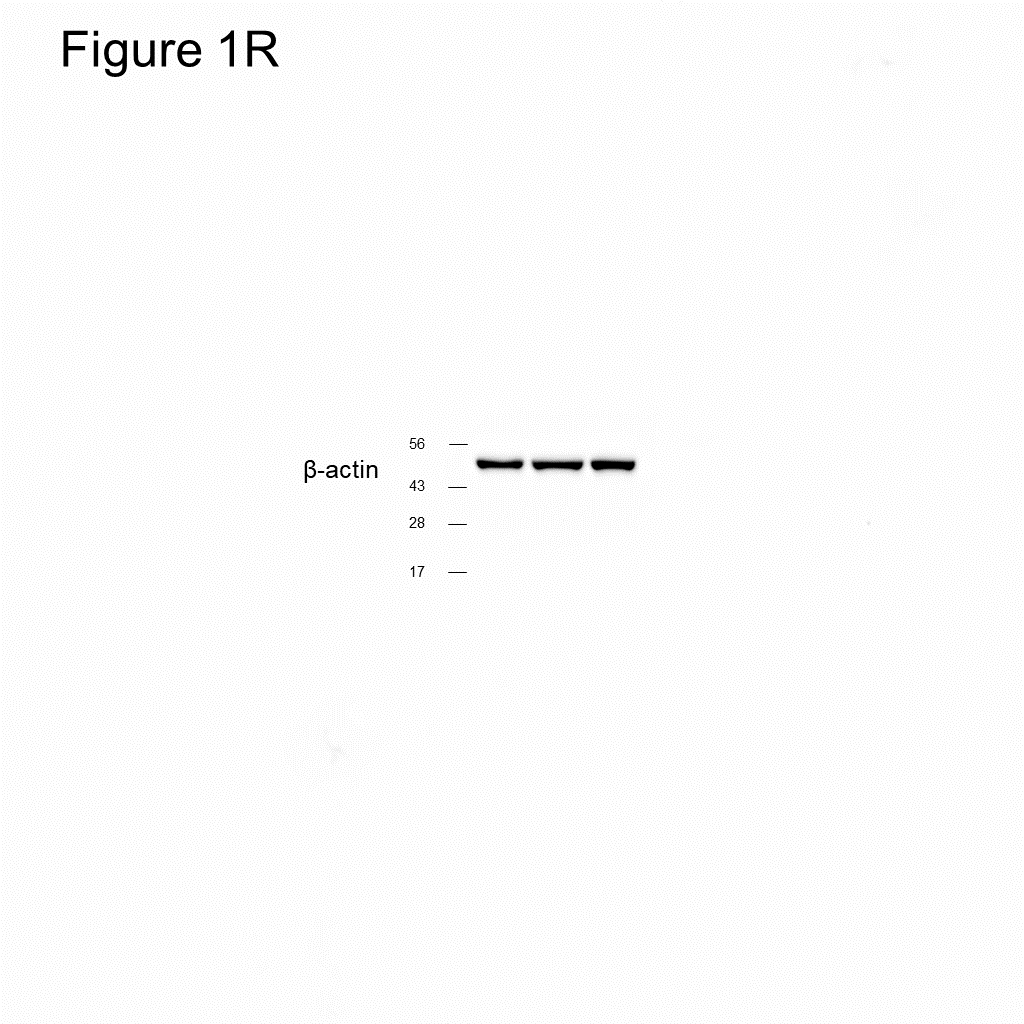


The original Full-length blots of the Fig. 1S


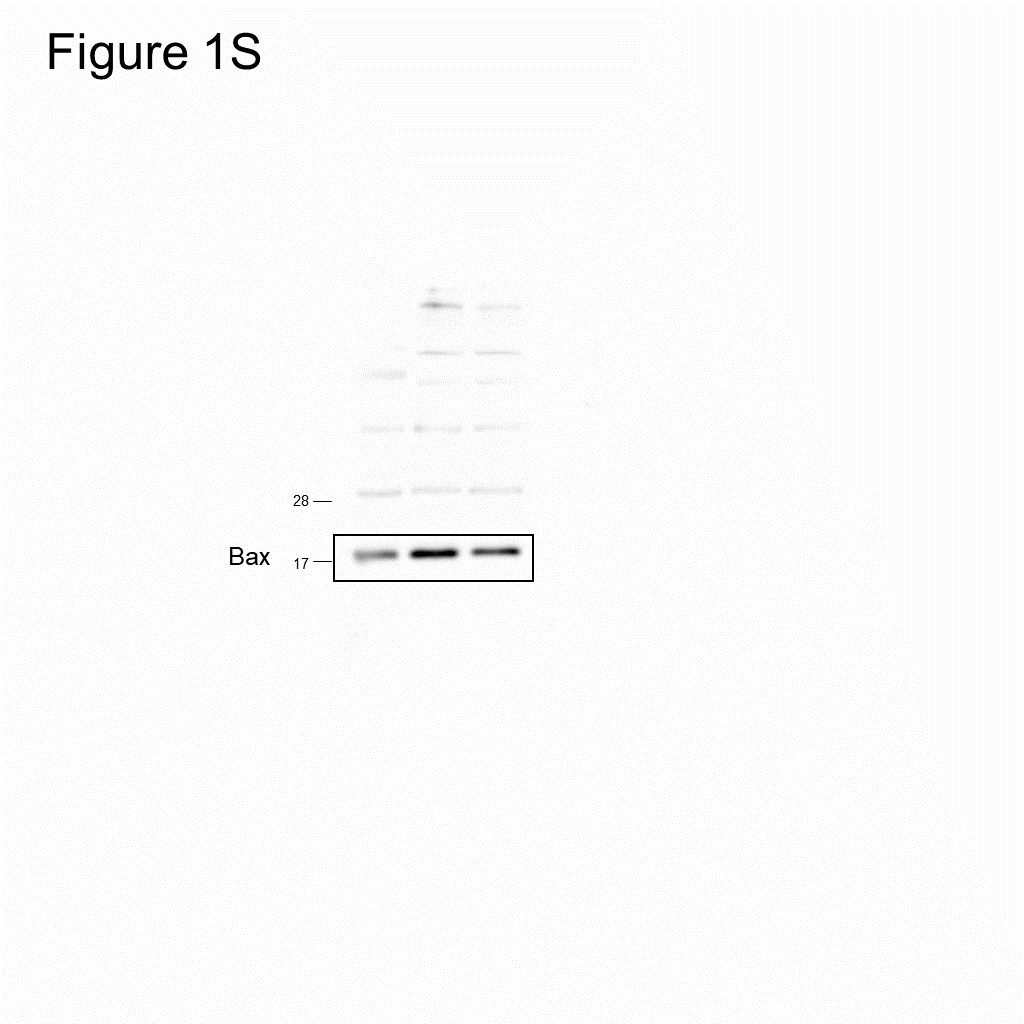

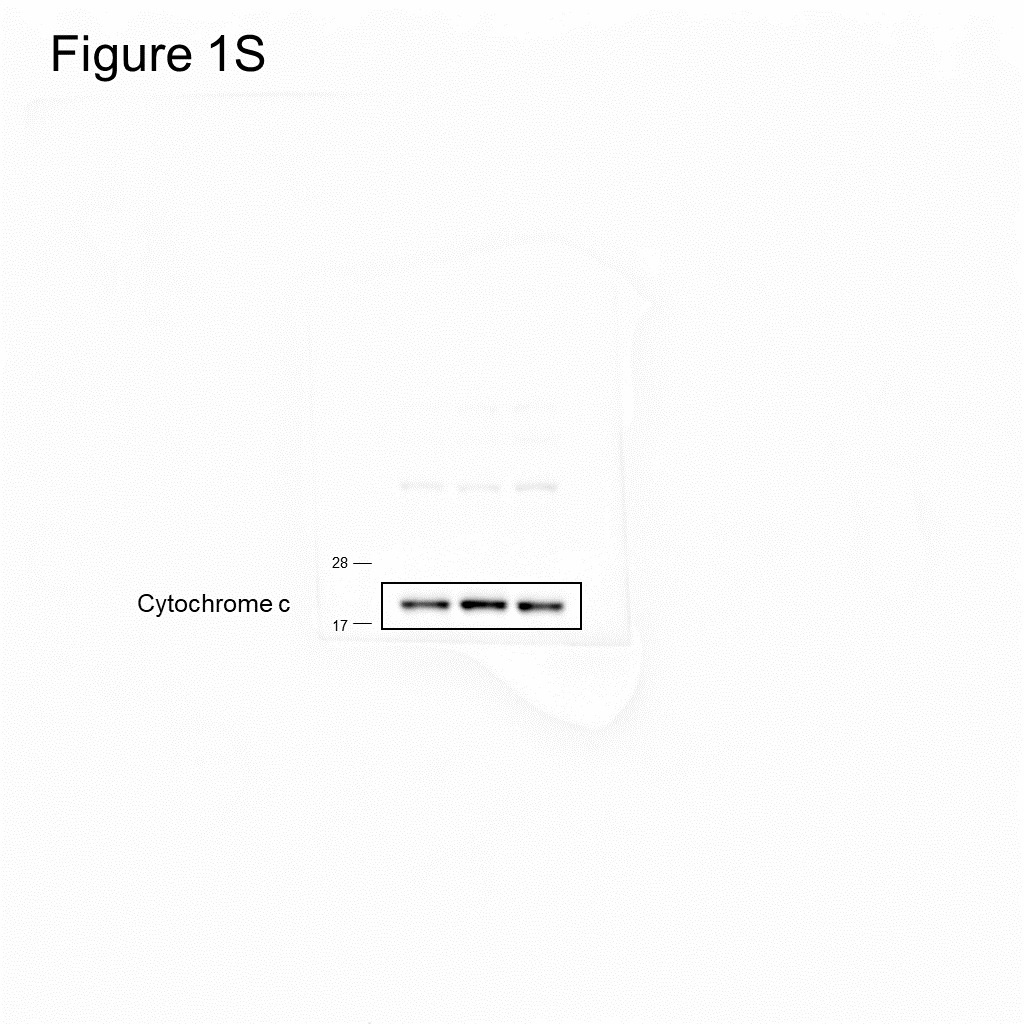

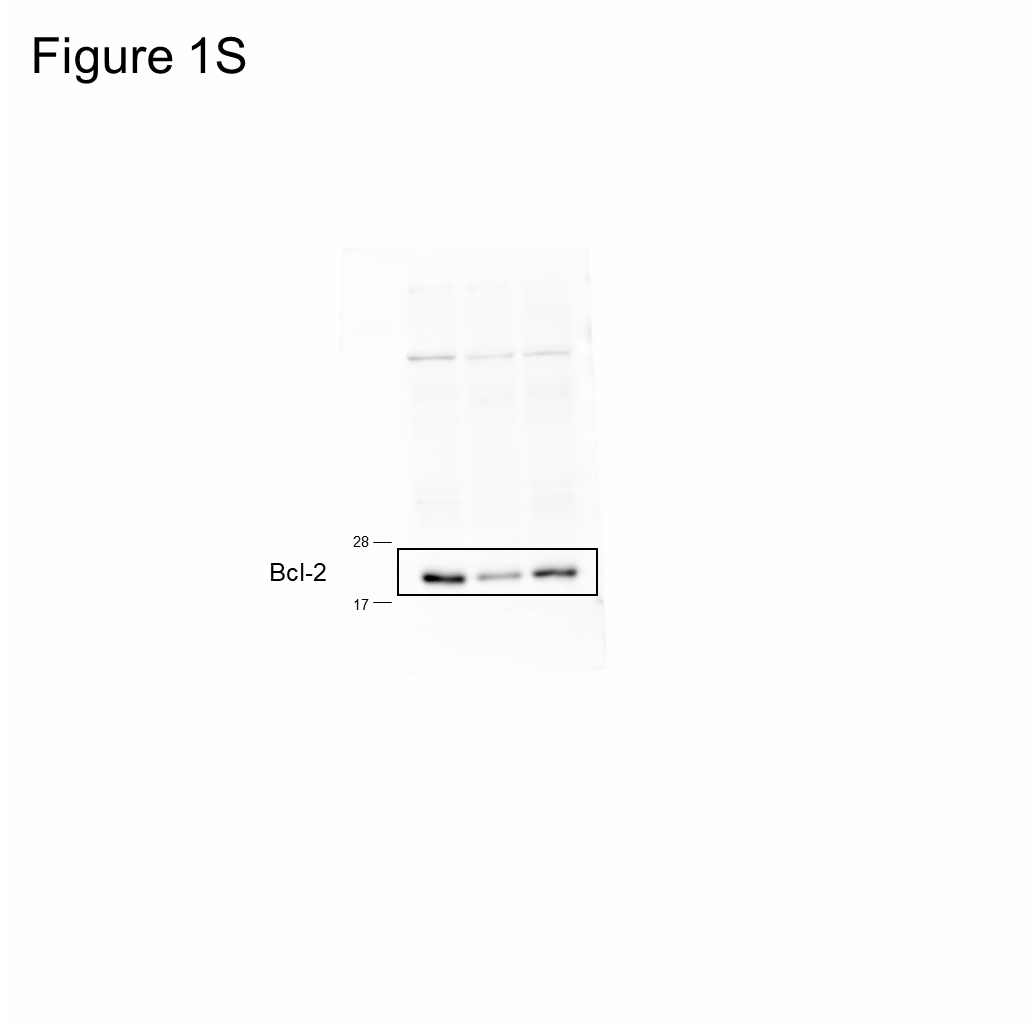

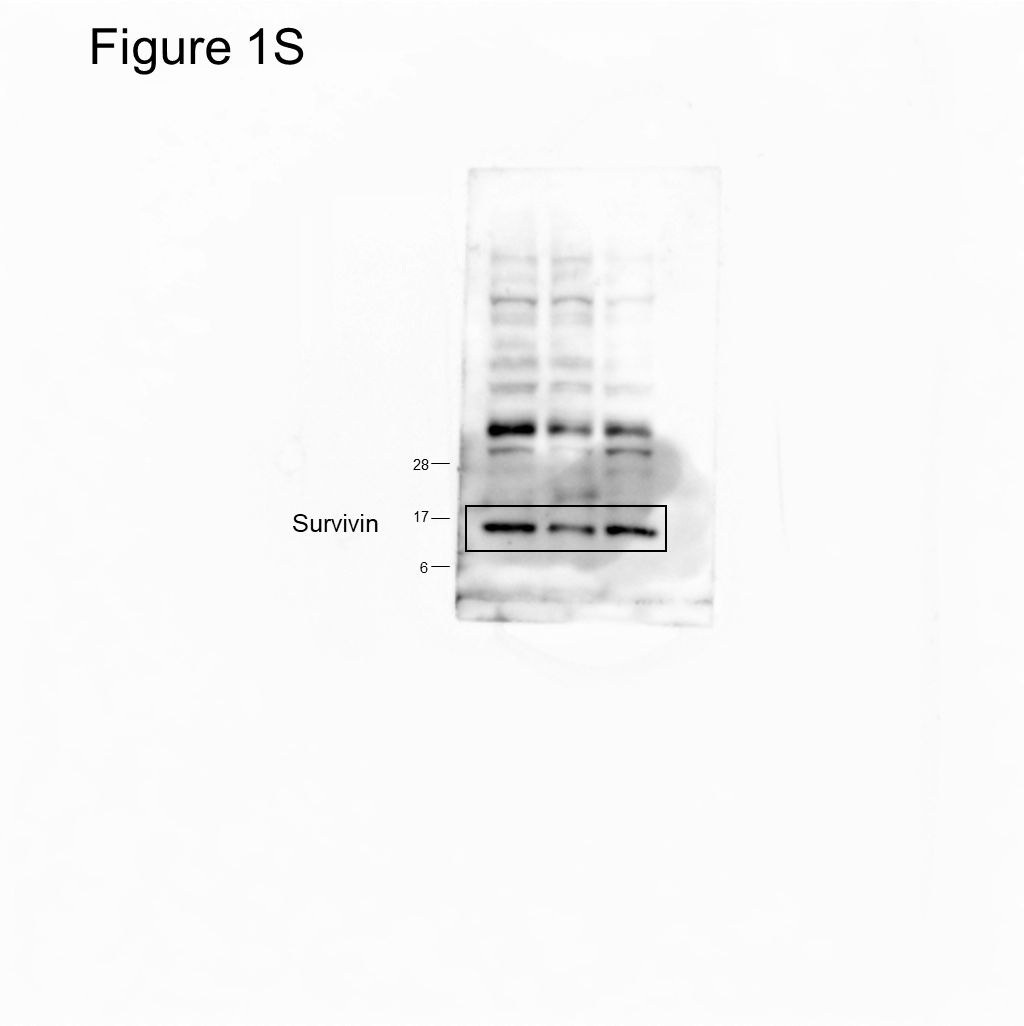

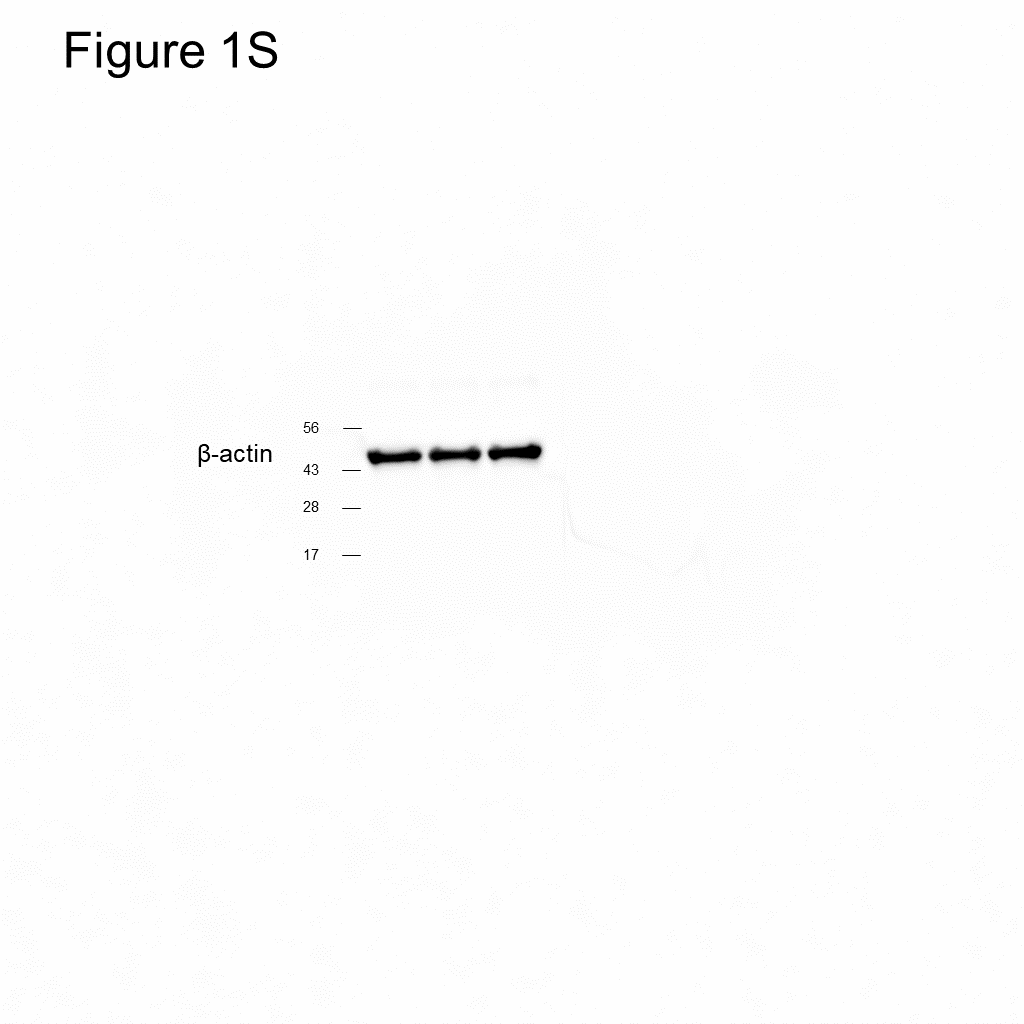


The original Full-length blots of the Fig. 2G


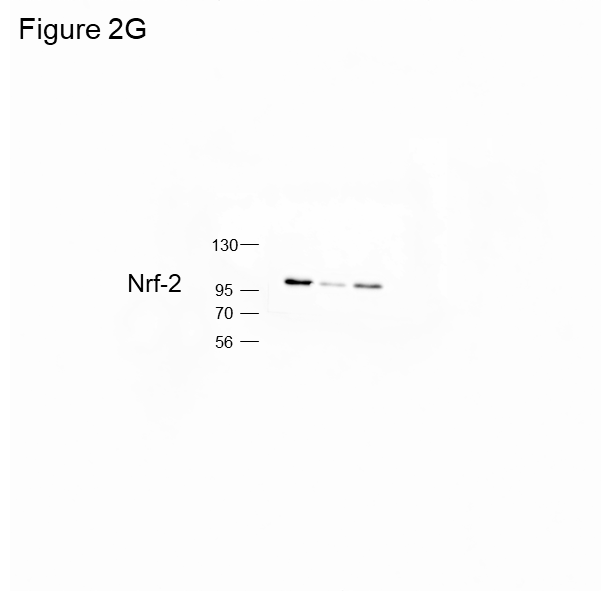

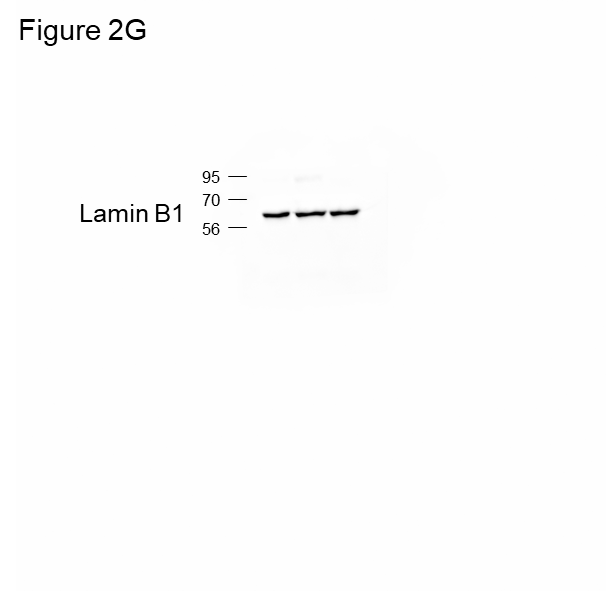

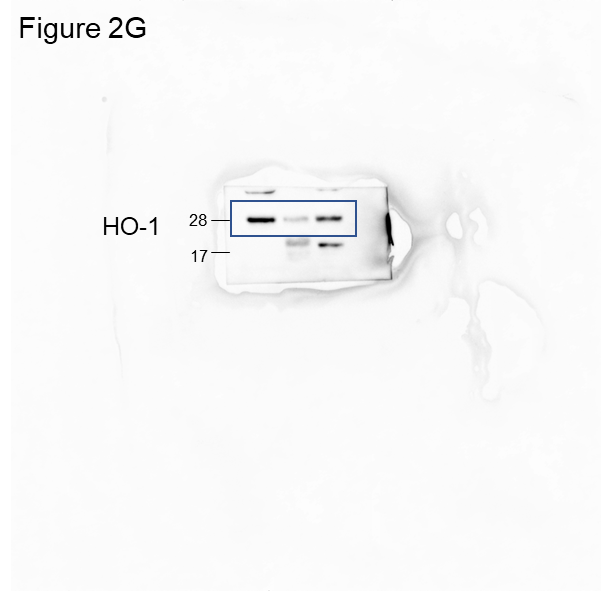

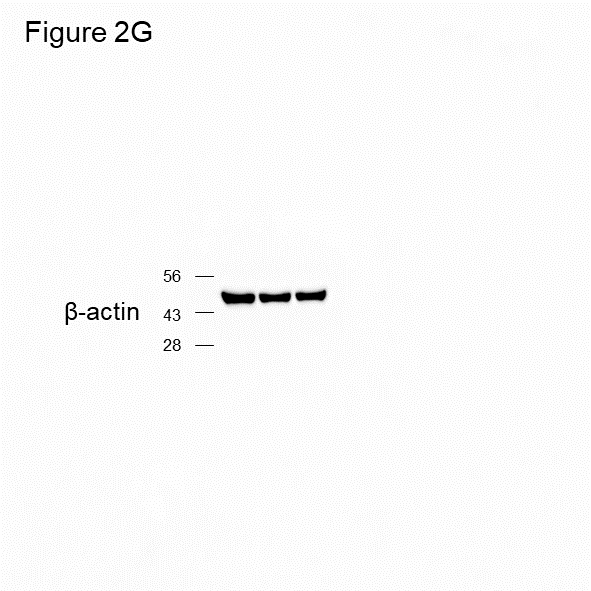


The original Full-length blots of the Fig. 2H


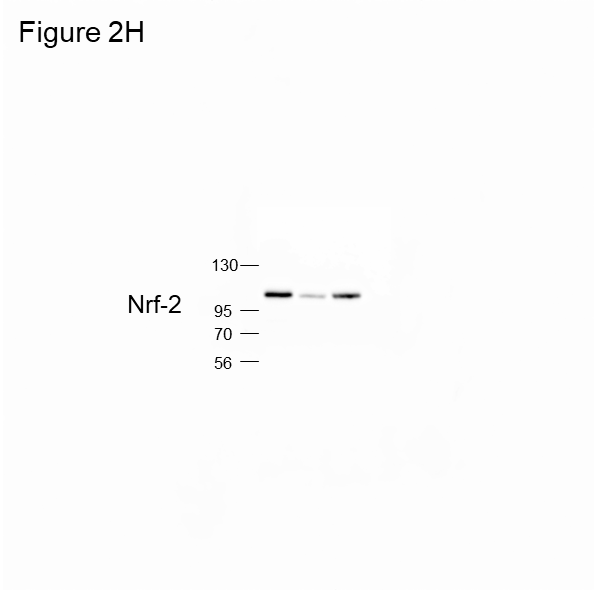

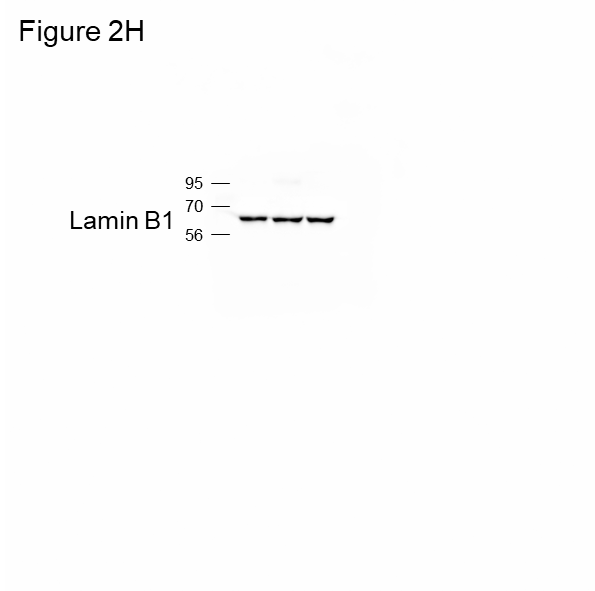

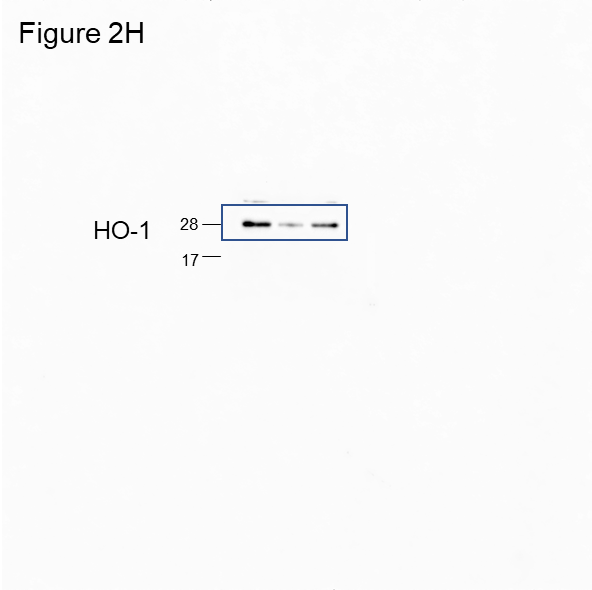


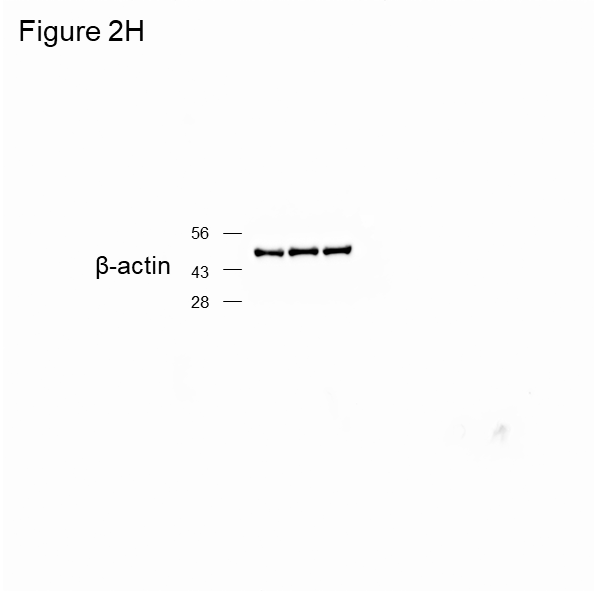


The original Full-length blots of the Fig. 2K


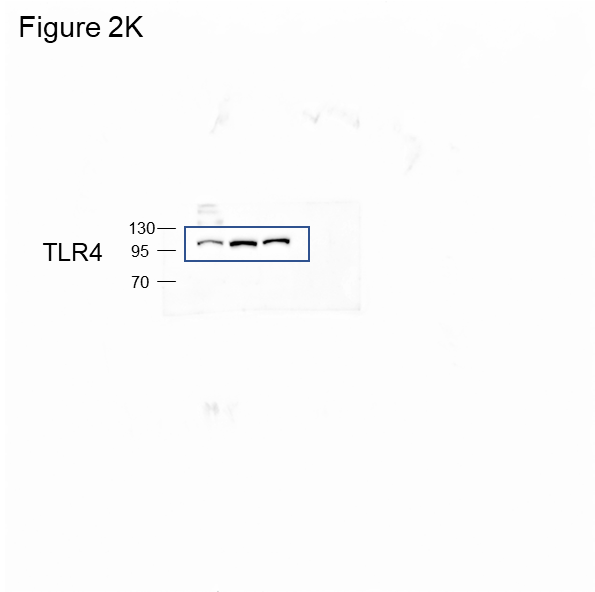

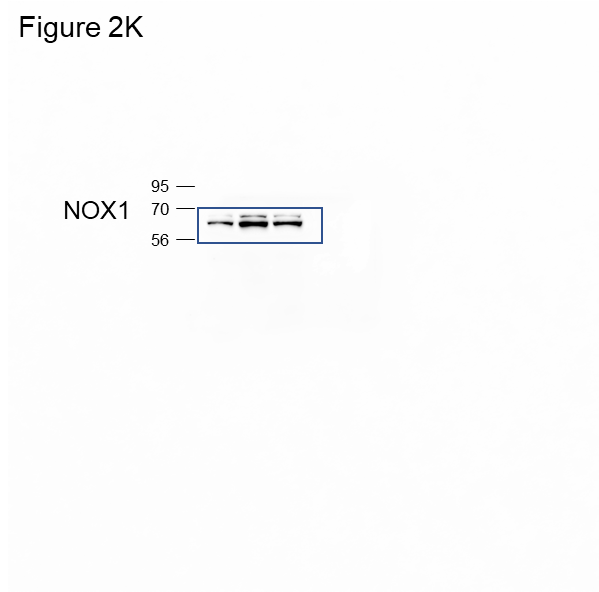

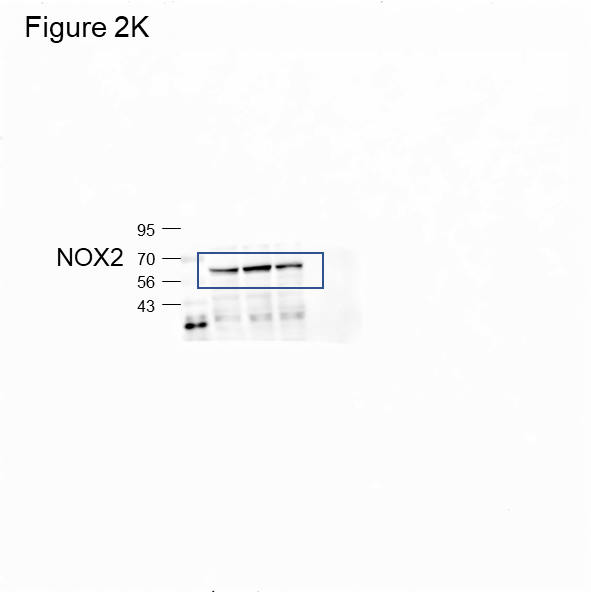


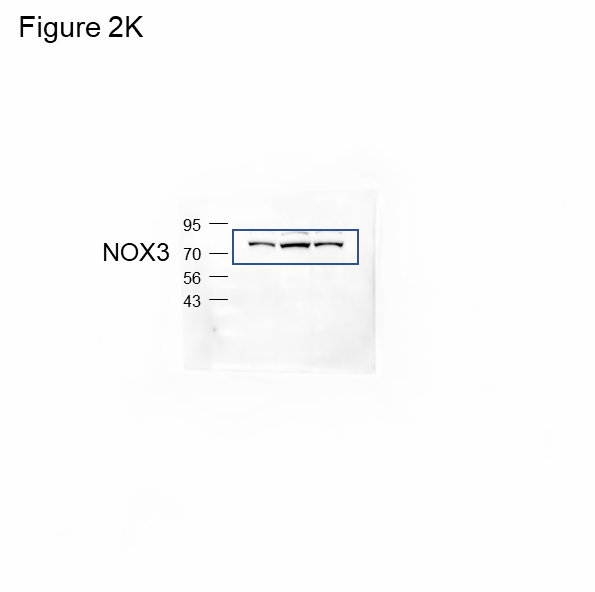

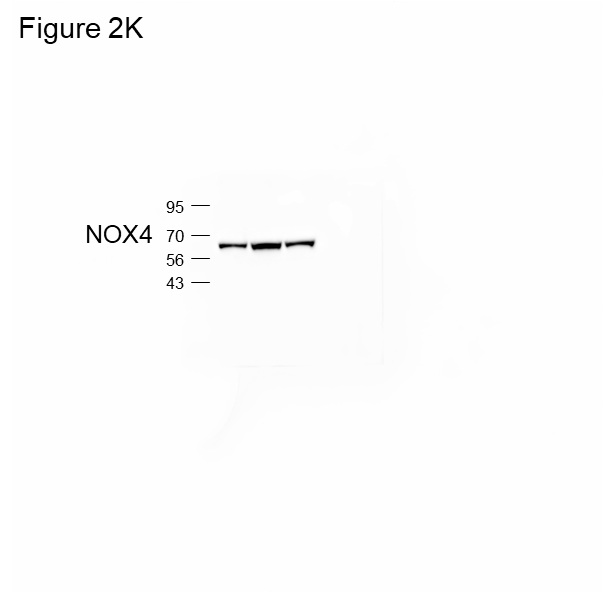

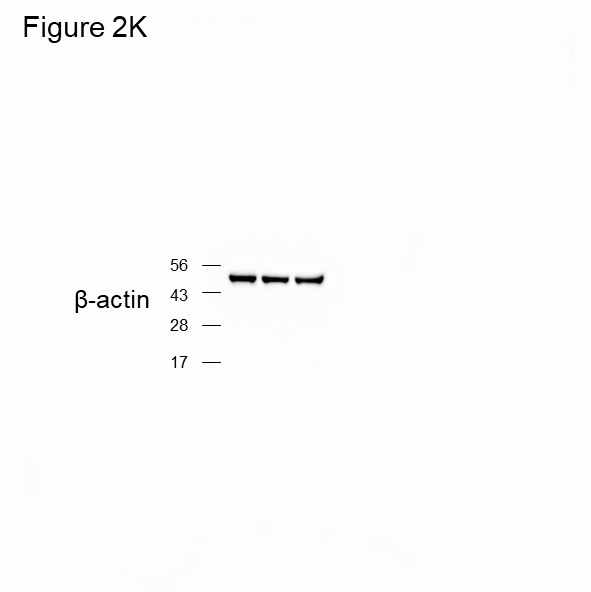


The original Full-length blots of the Fig. 2L


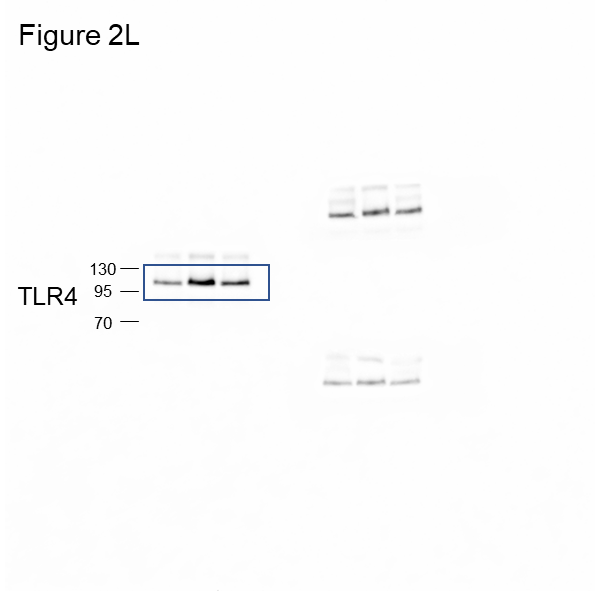

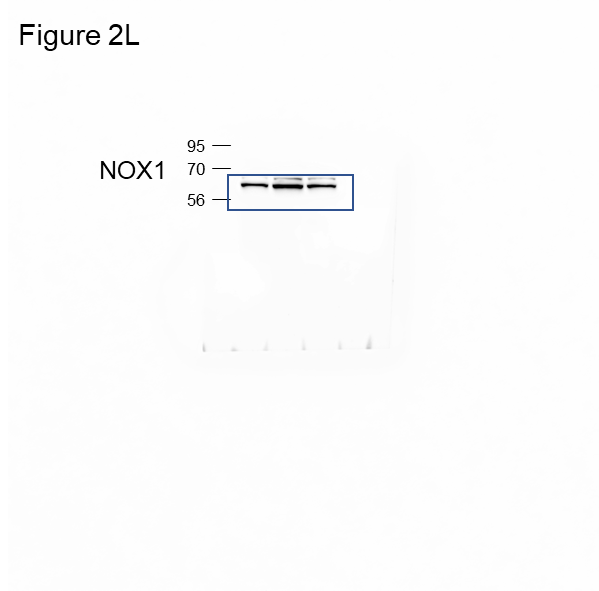

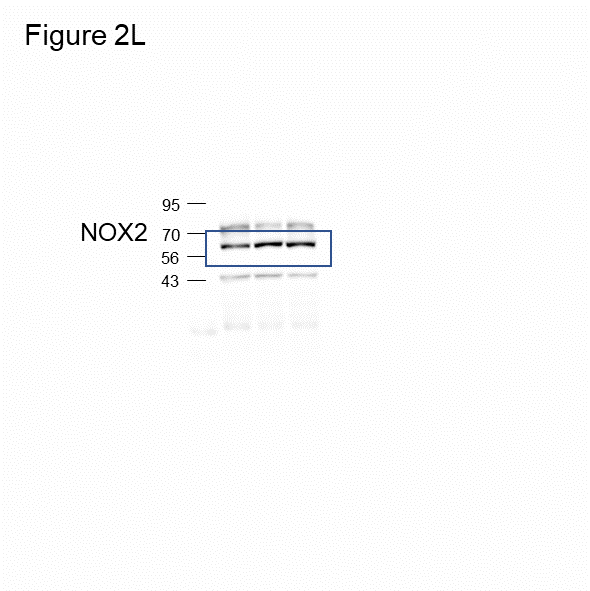


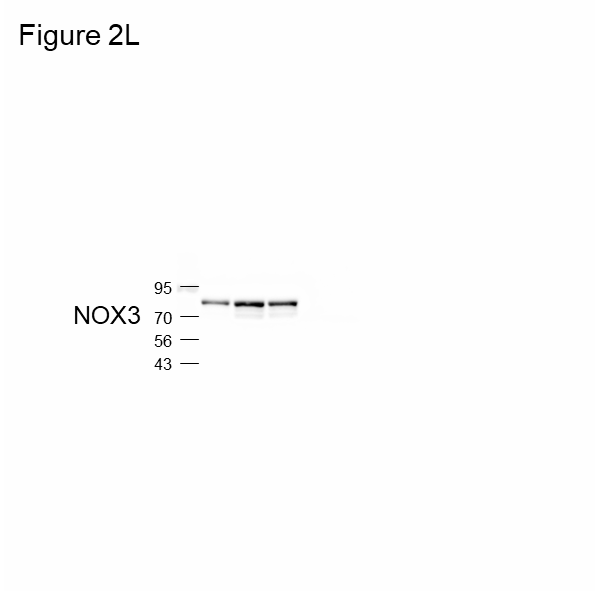

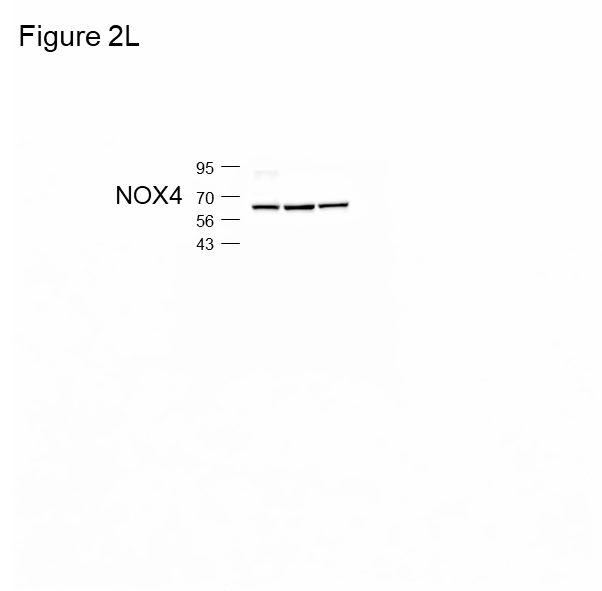

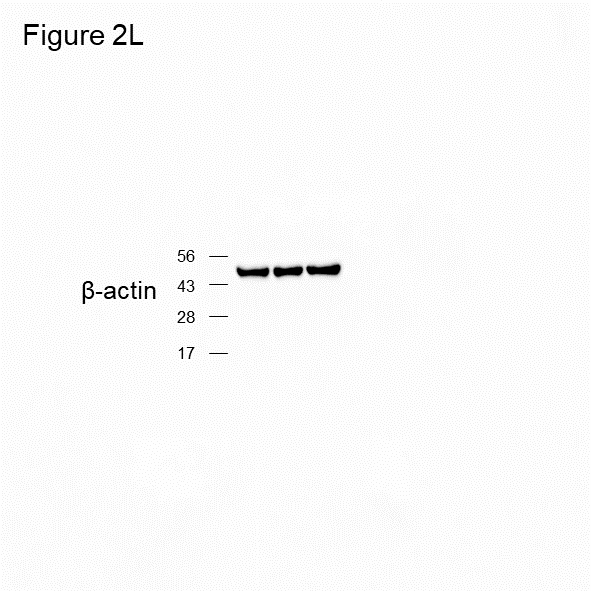


The original Full-length blots of the Fig. 3C


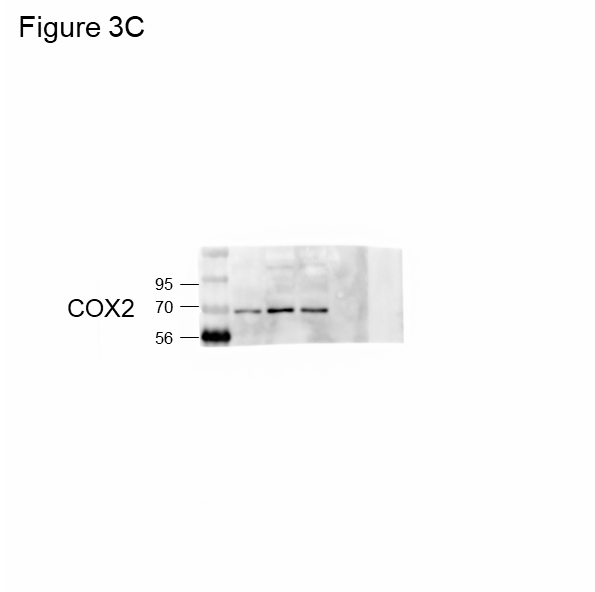

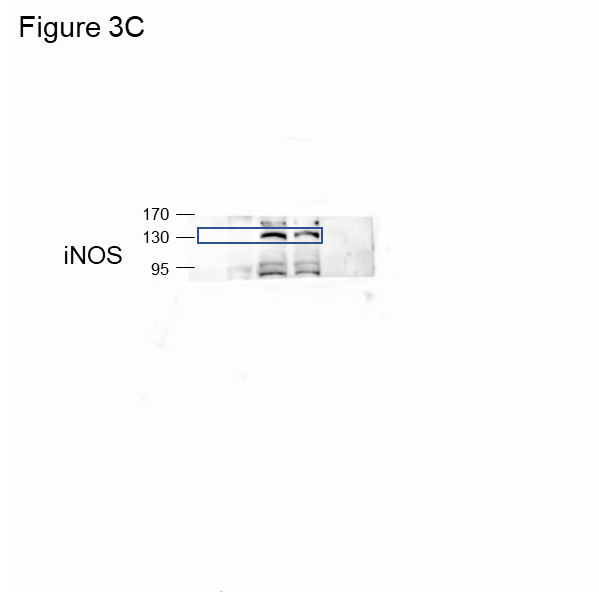

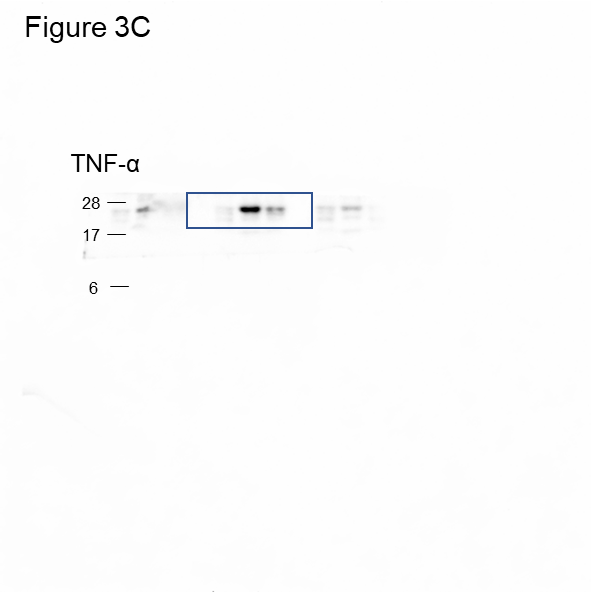


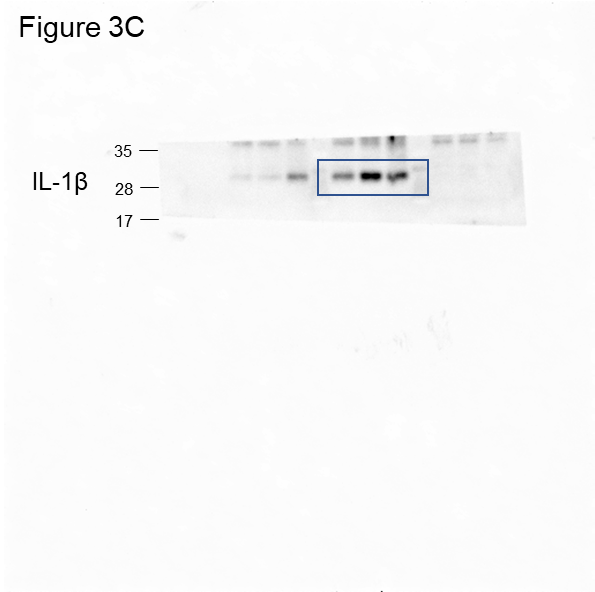

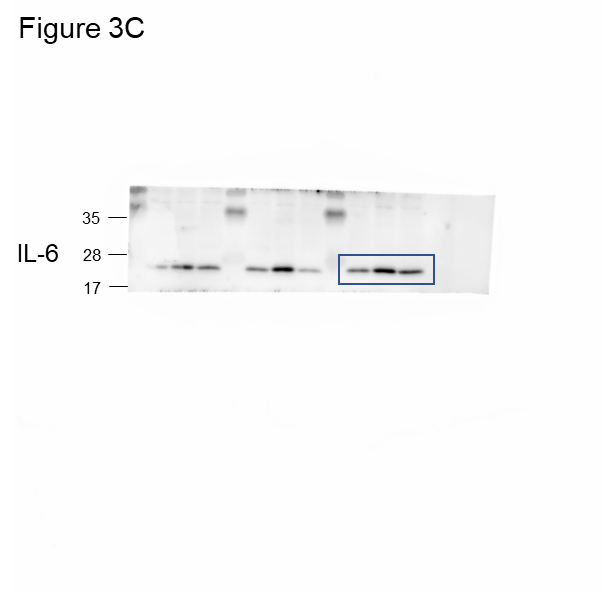

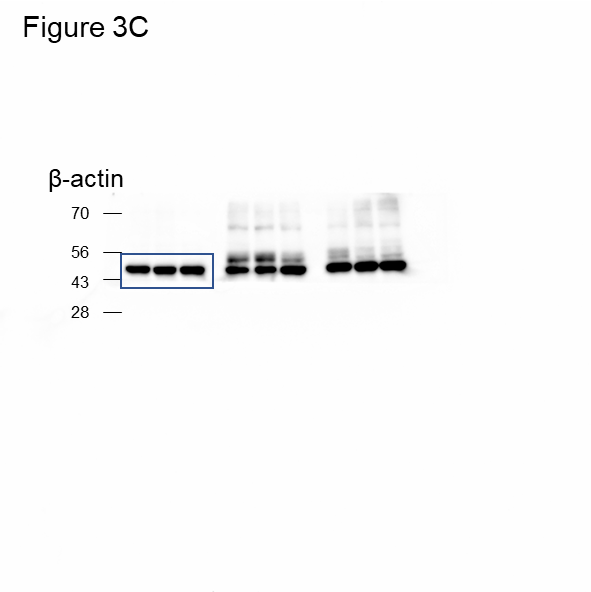


The original Full-length blots of the Fig. 3H


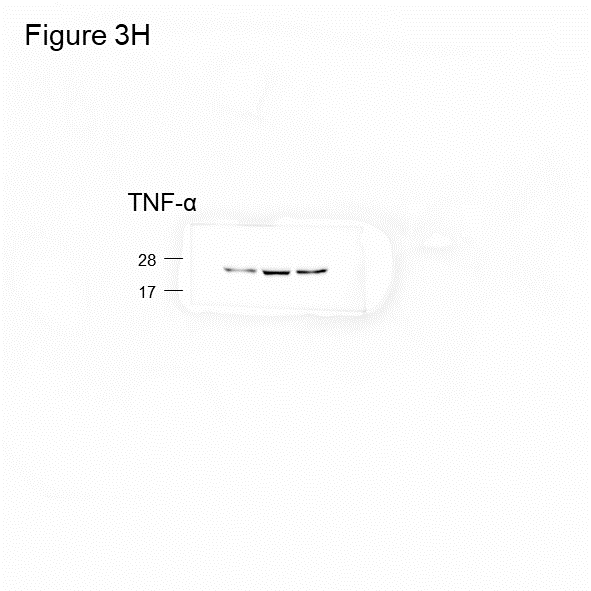

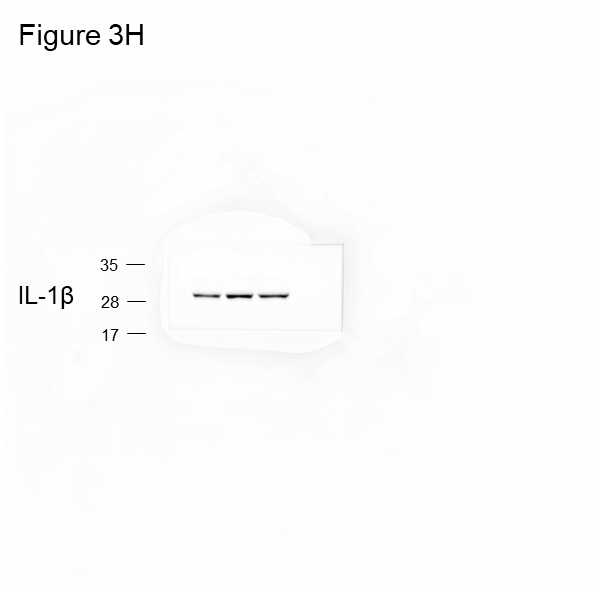

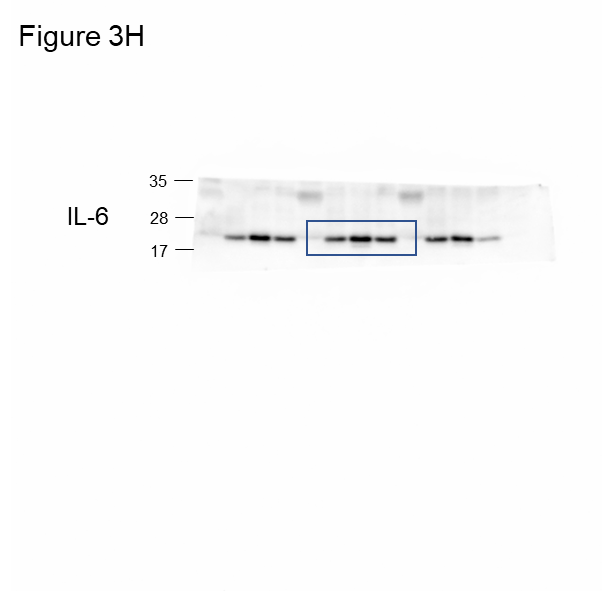

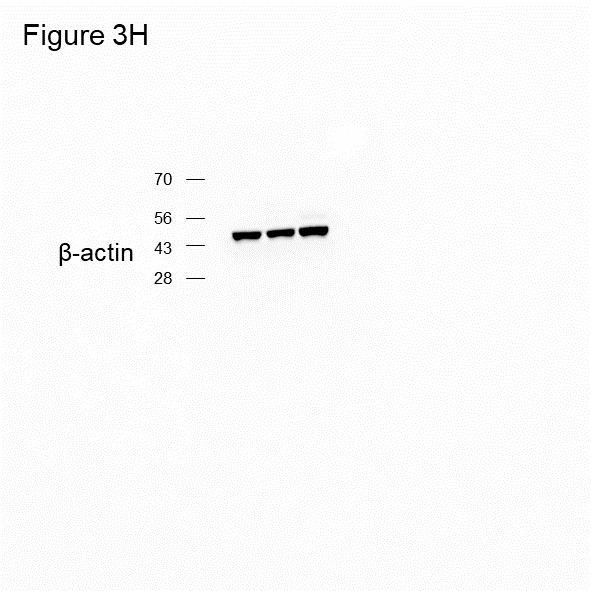


The original Full-length blots of the Fig. 3K


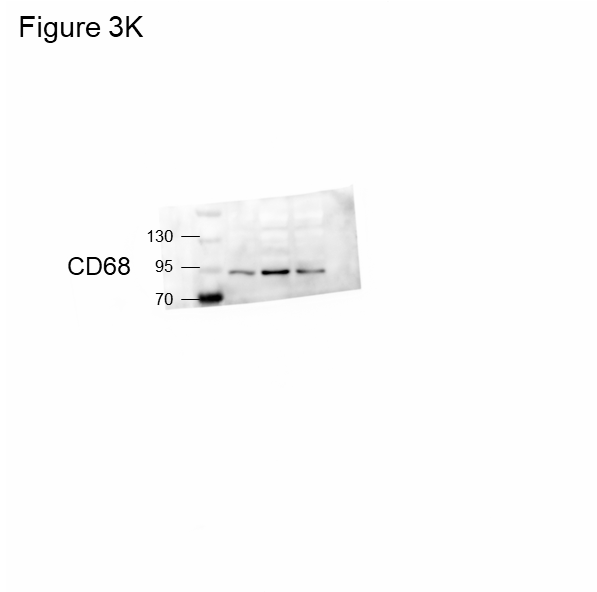

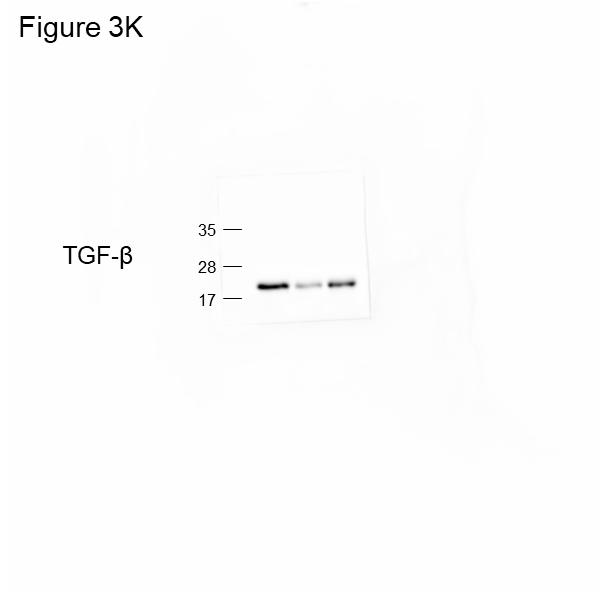

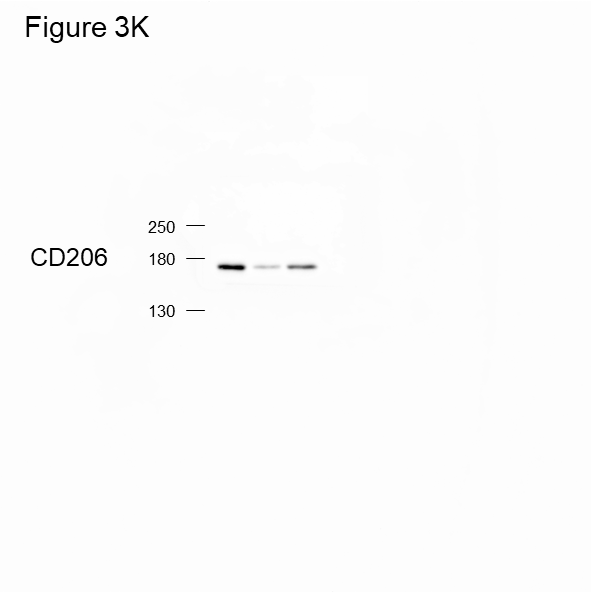


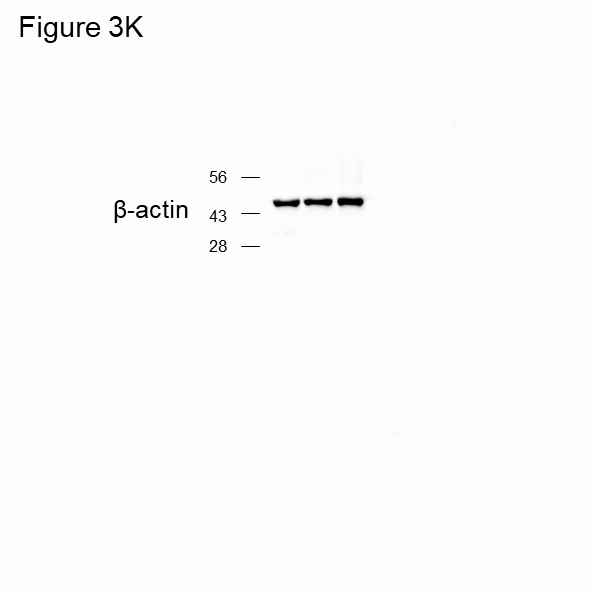


The original Full-length blots of the Fig. 3L


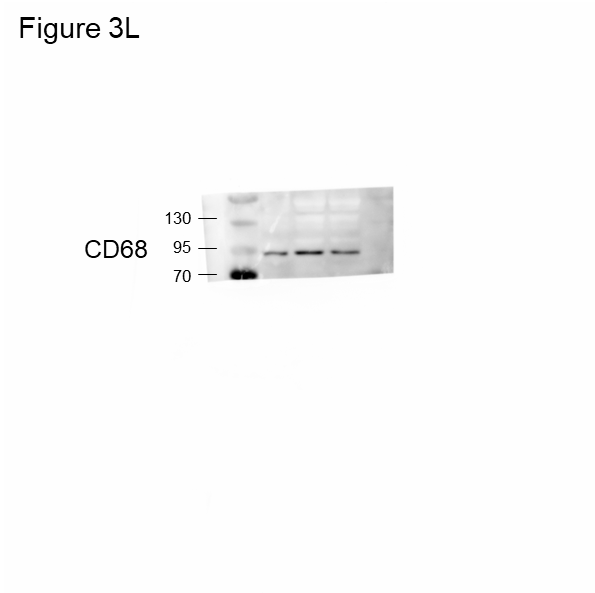

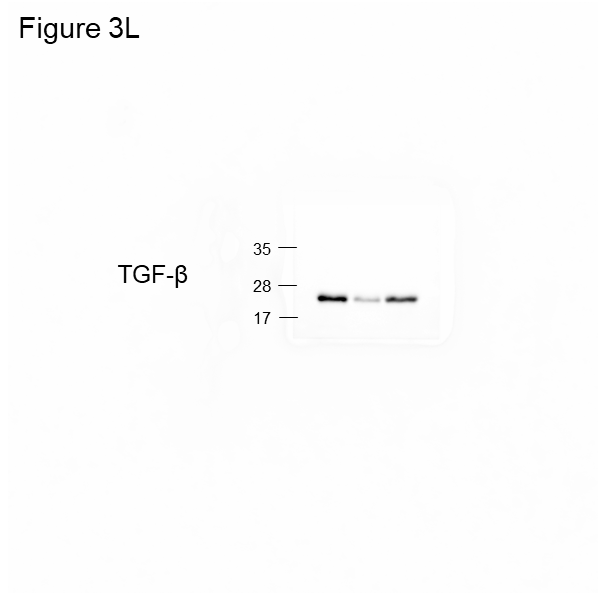

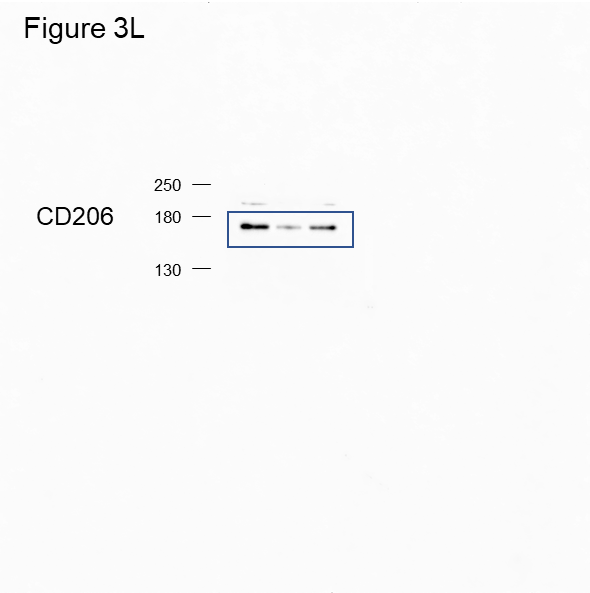

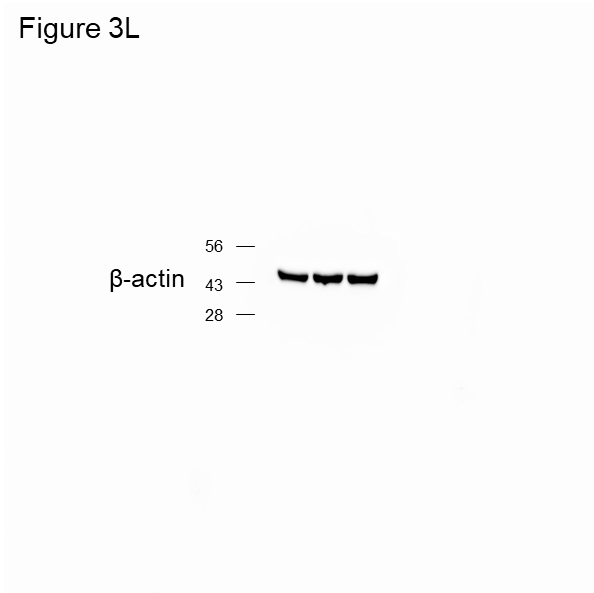


The original Full-length blots of the Fig. 4D


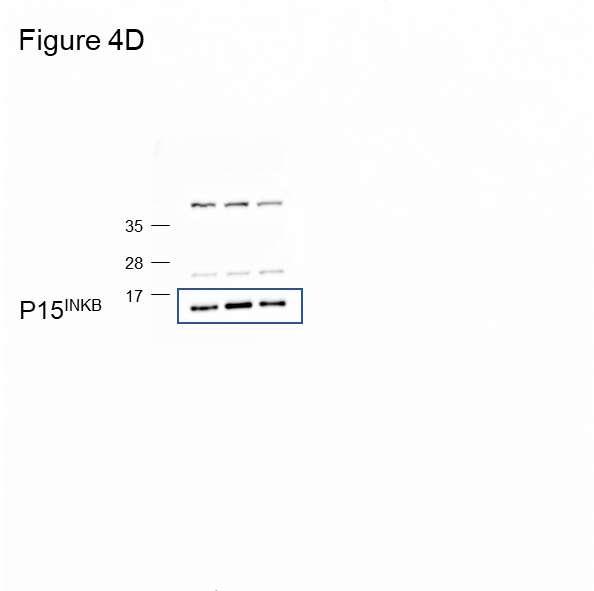

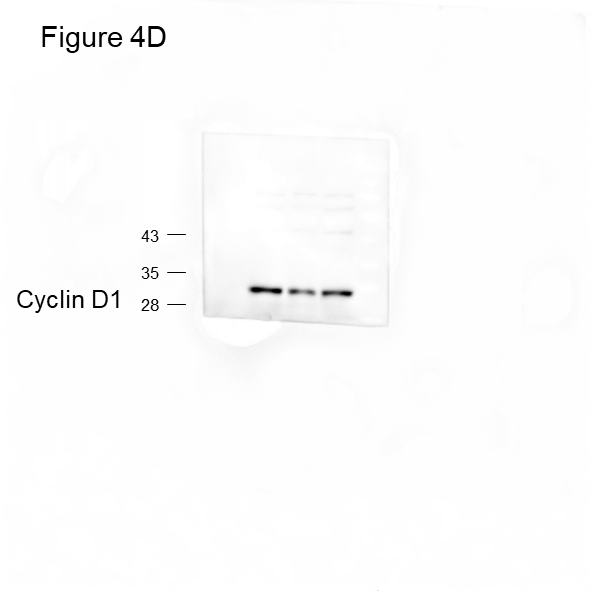

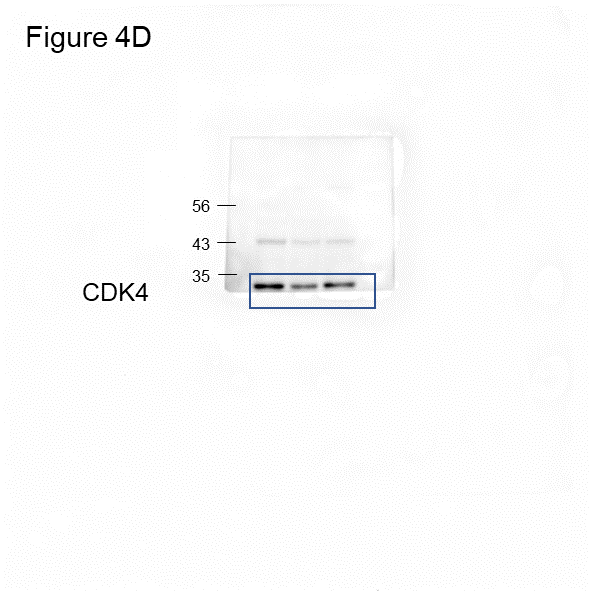


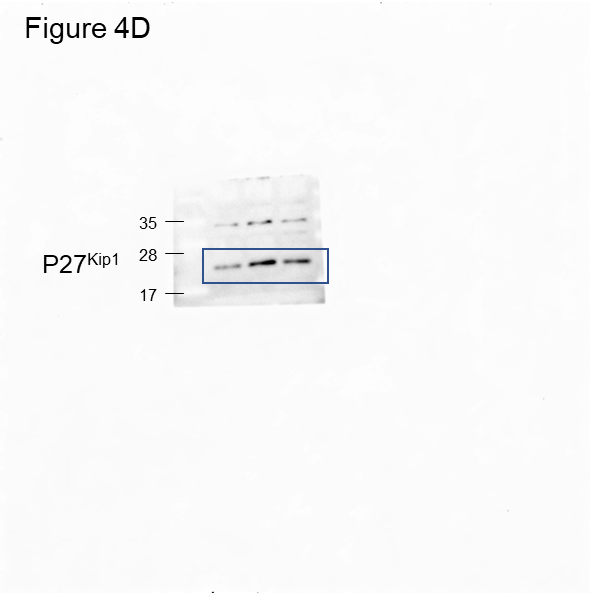

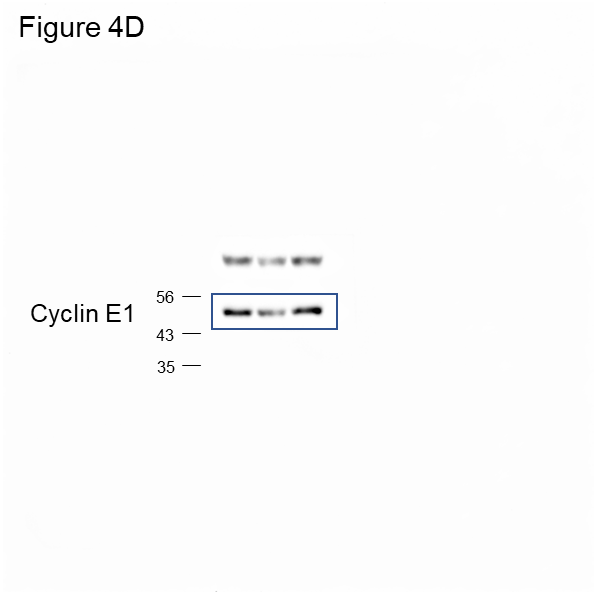

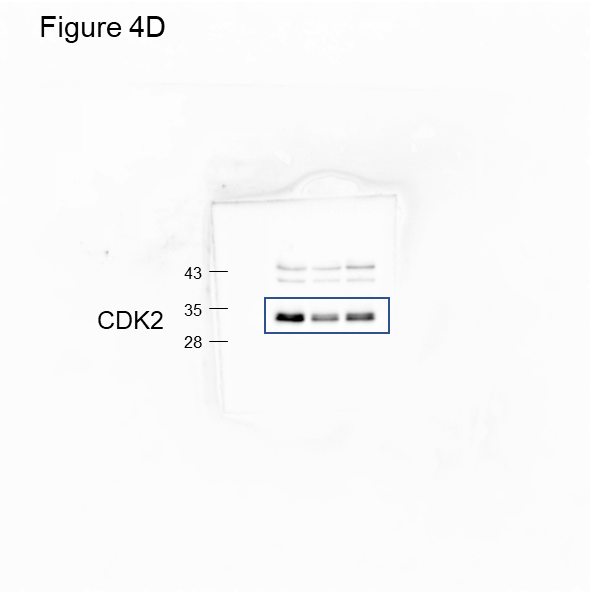


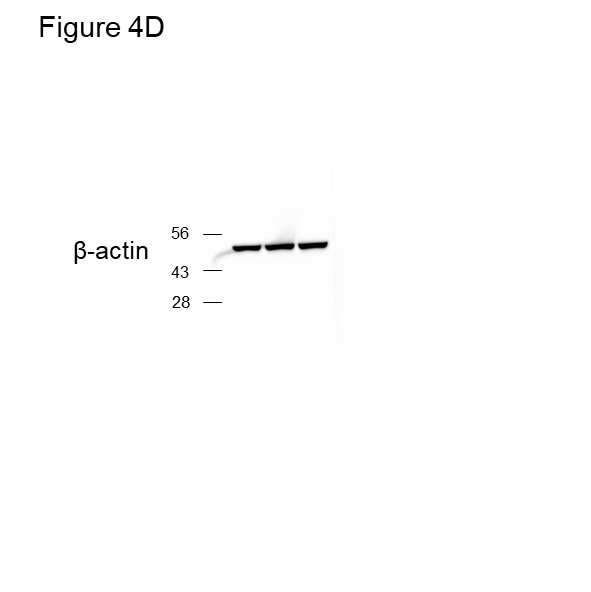


The original Full-length blots of the Fig. 4N


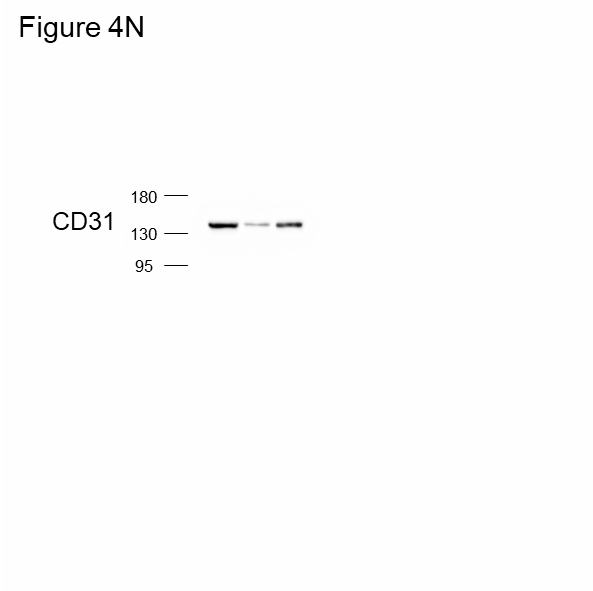

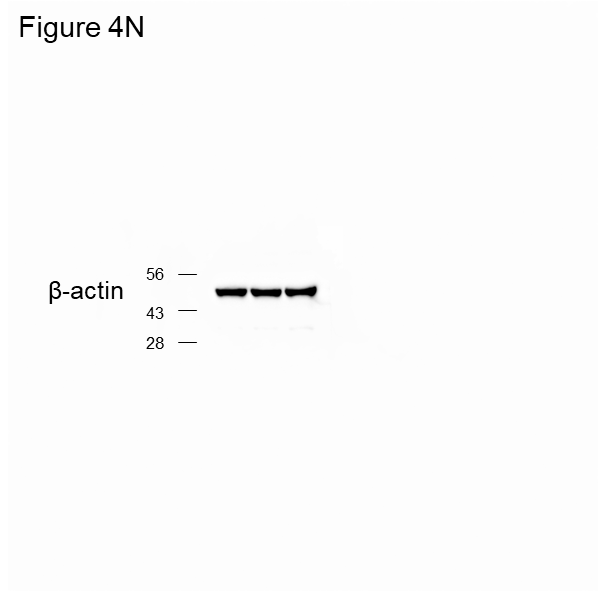

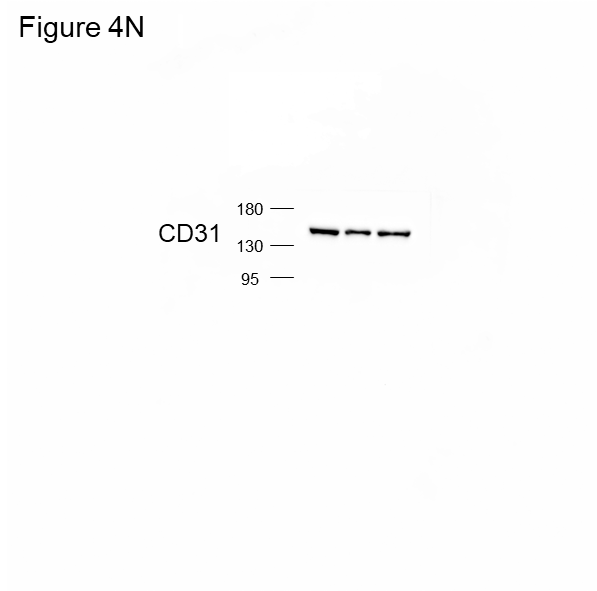

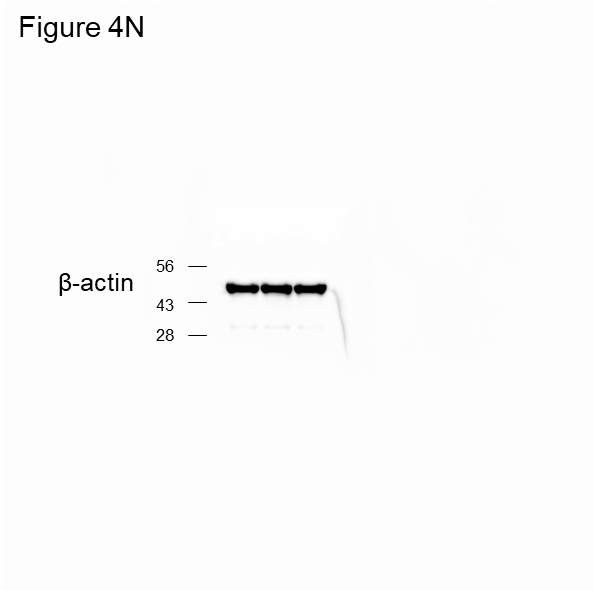


The original Full-length blots of the Fig. 5K


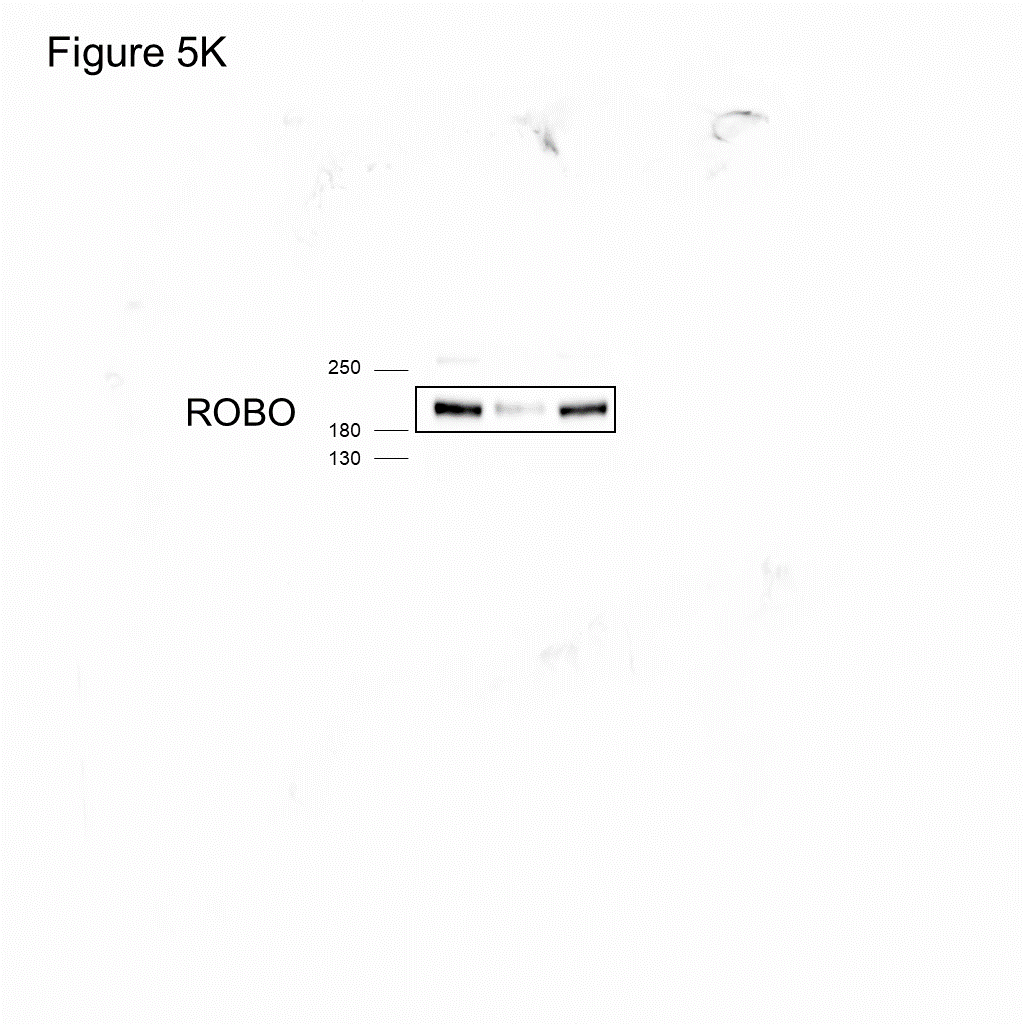

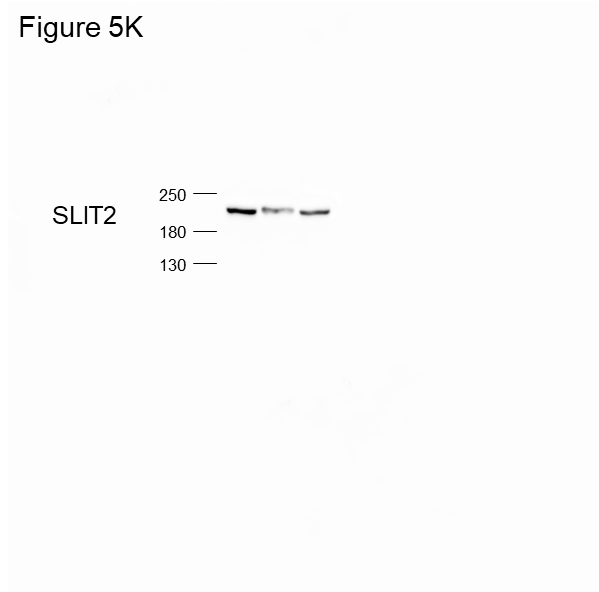

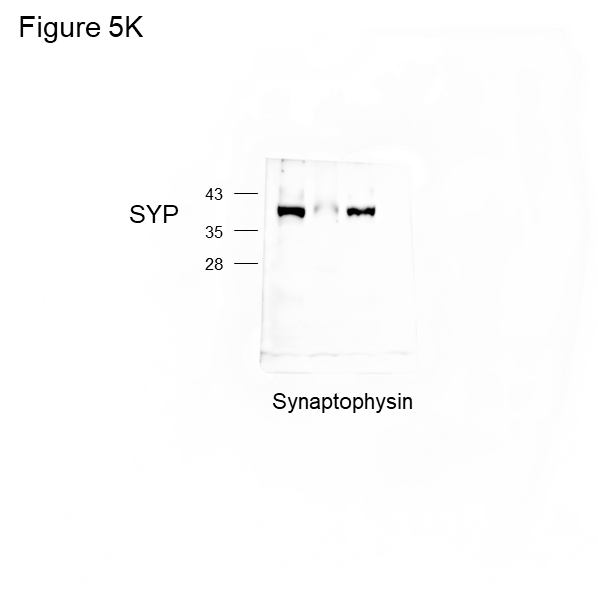

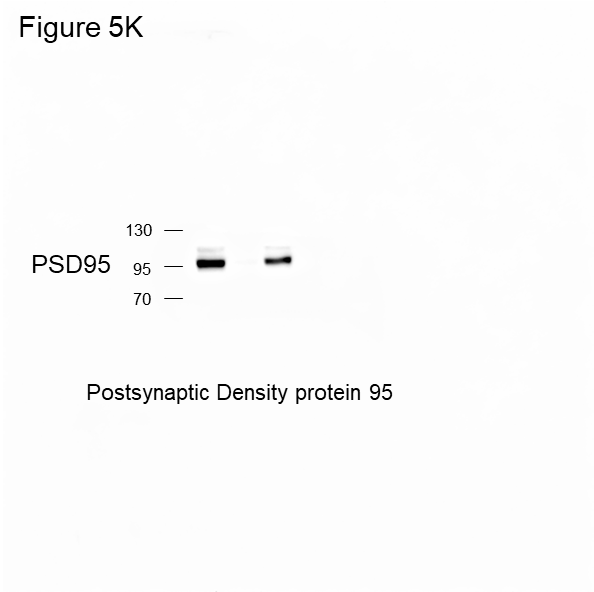

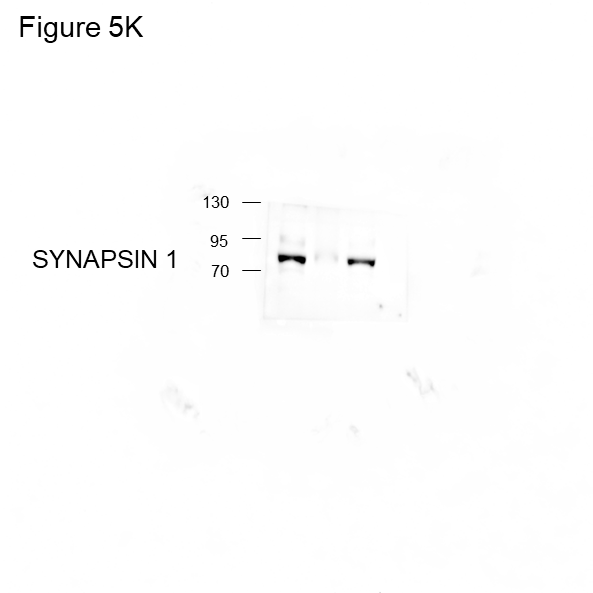

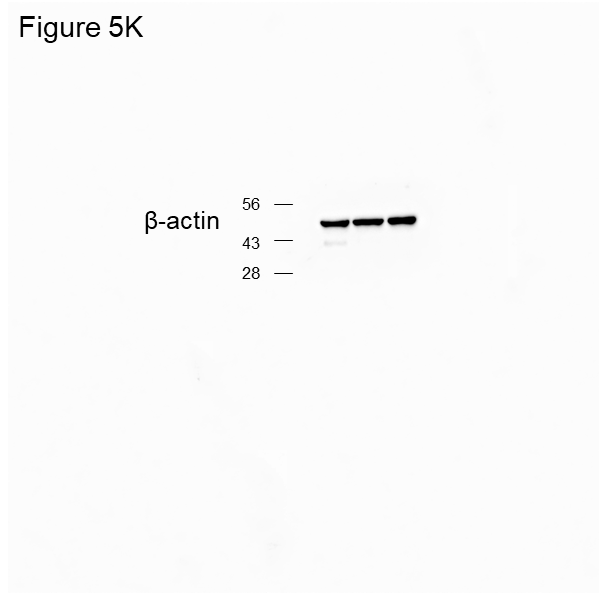


The original Full-length blots of the Fig. 5Q


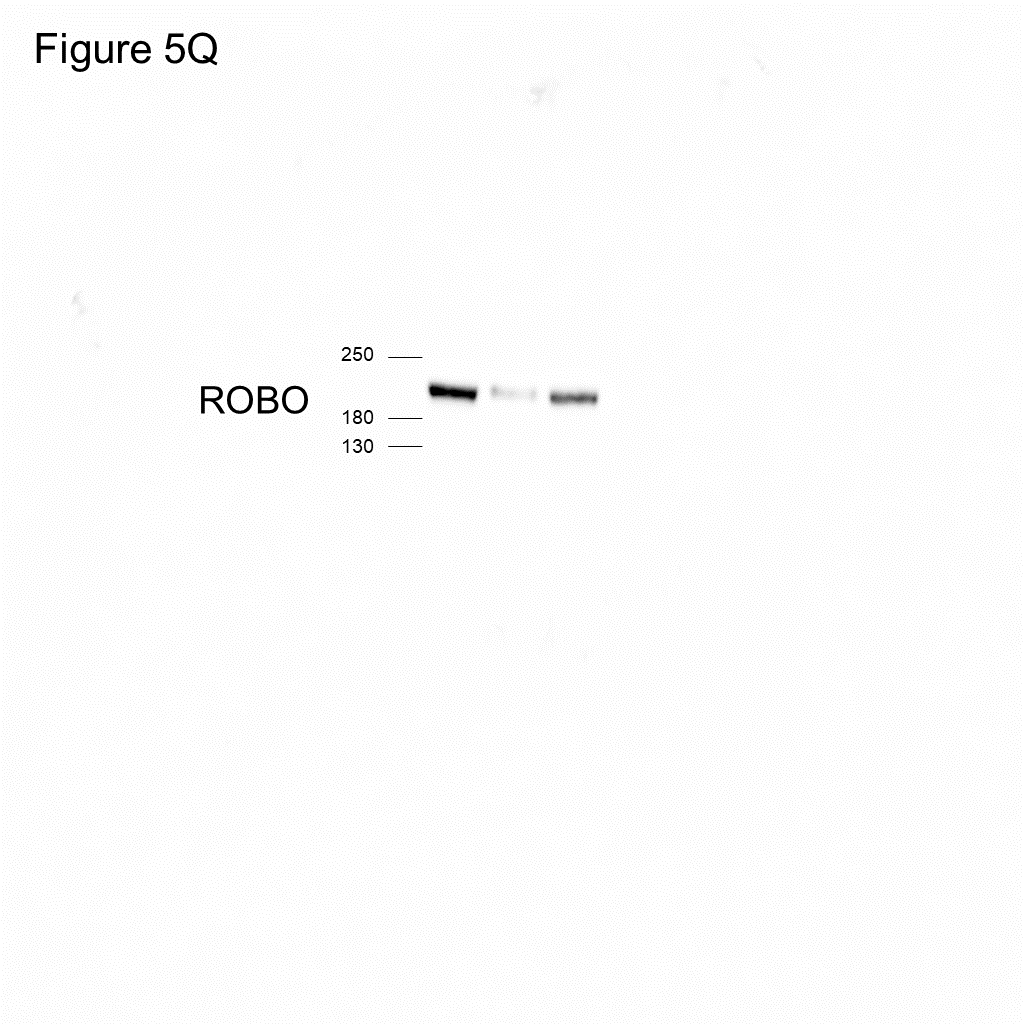

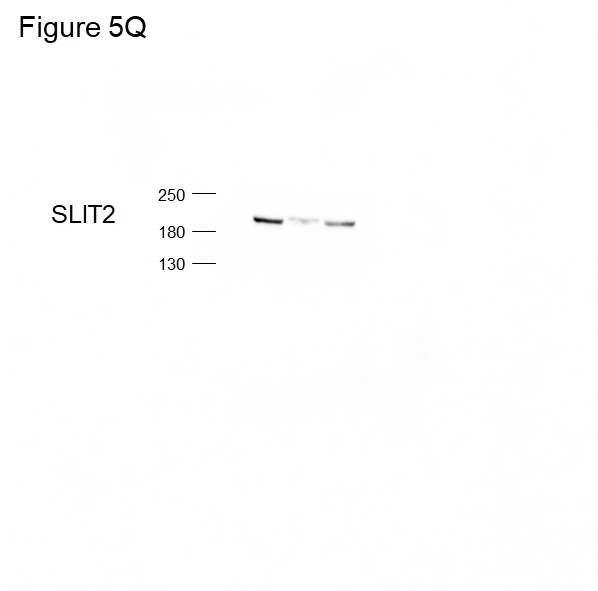

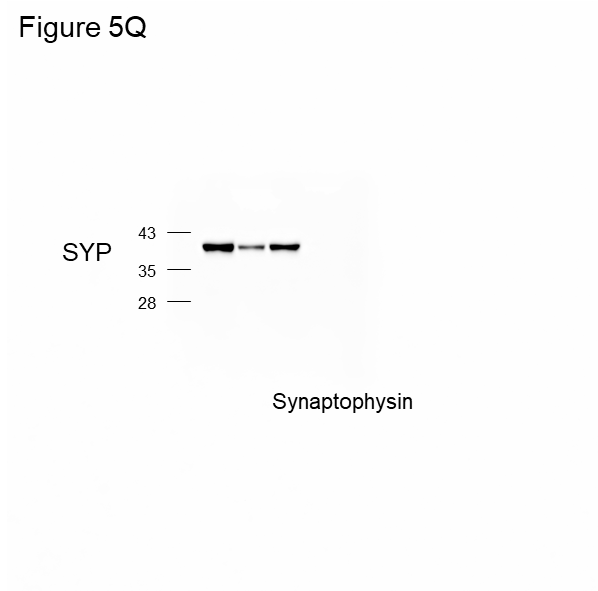

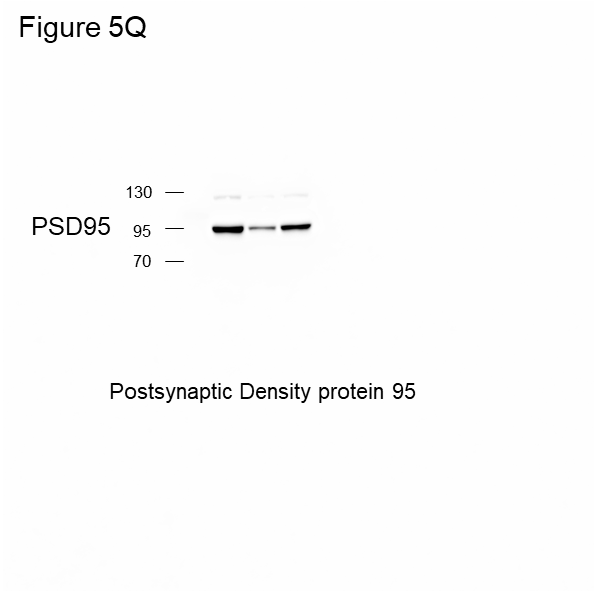

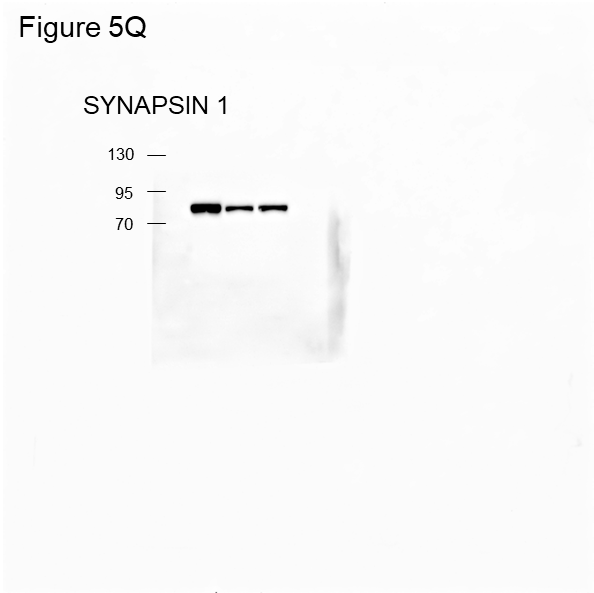

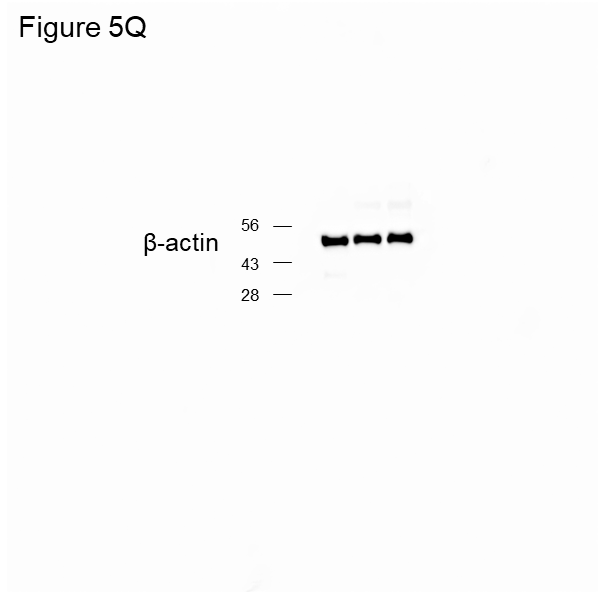


The original Full-length blots of the Supple Fig. 1B


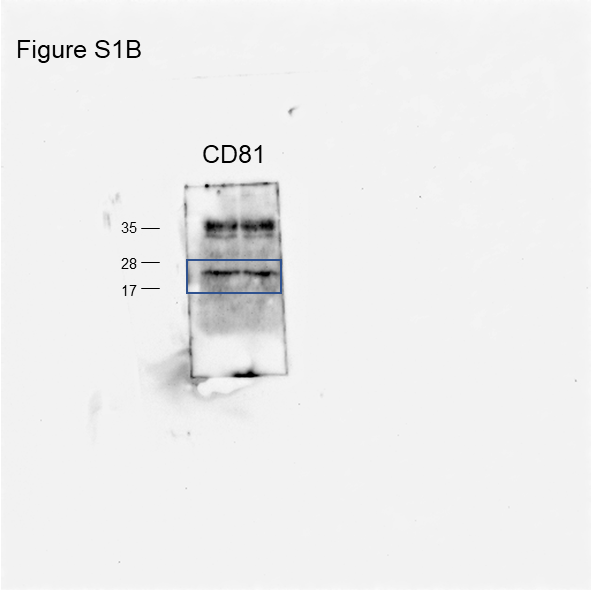

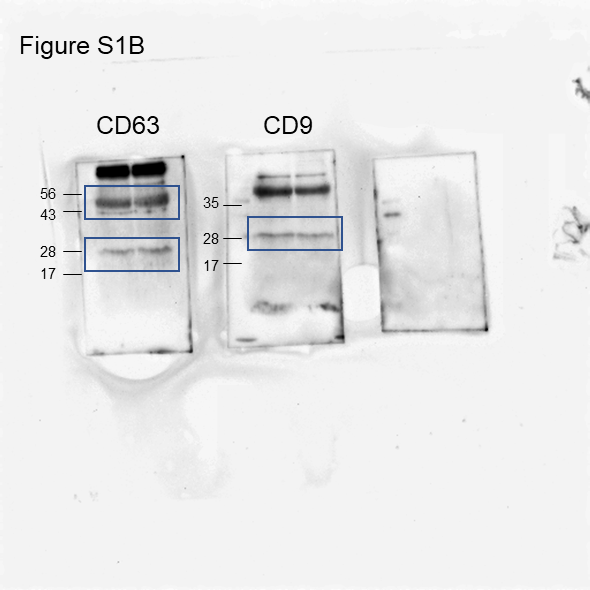

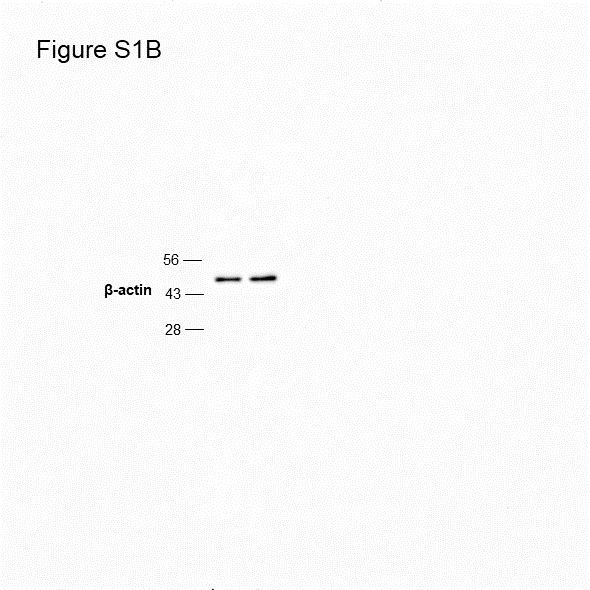


The original Full-length blots of the Supple Fig. 4C


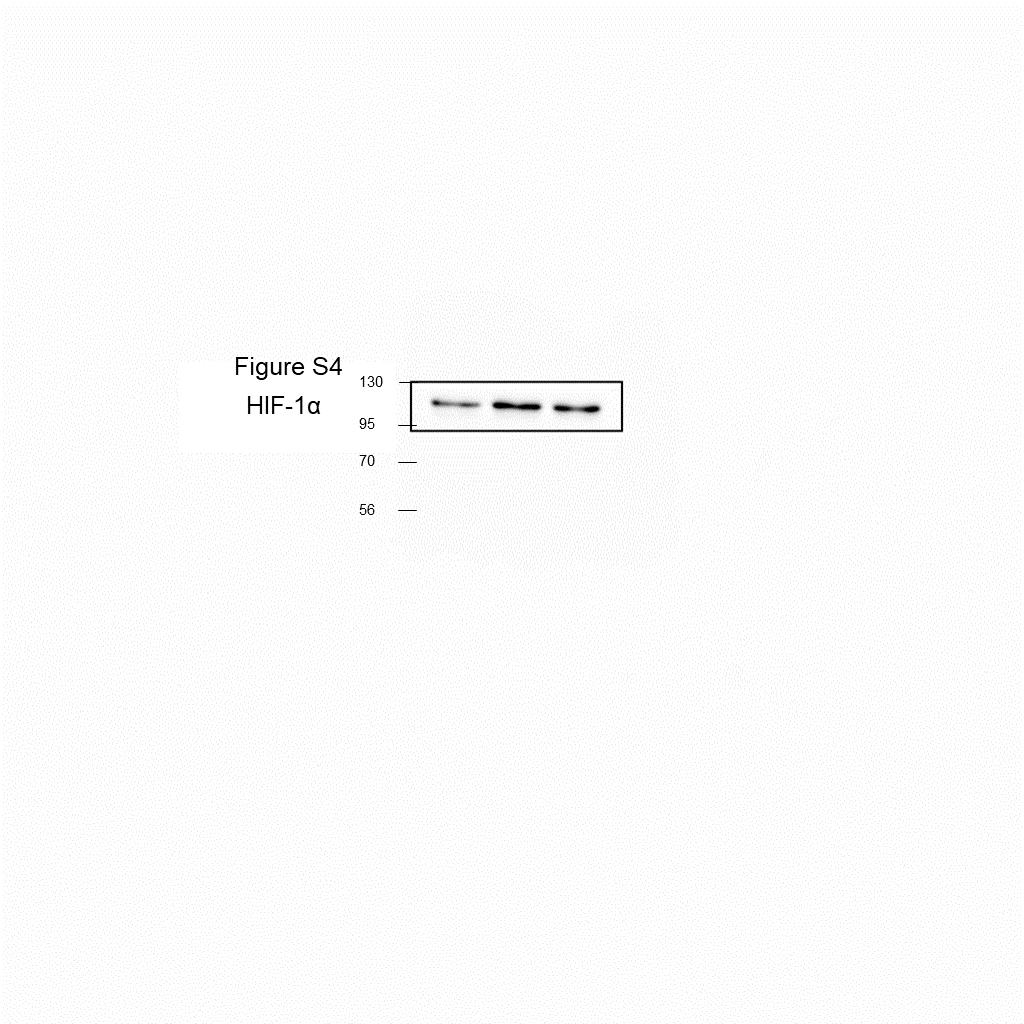

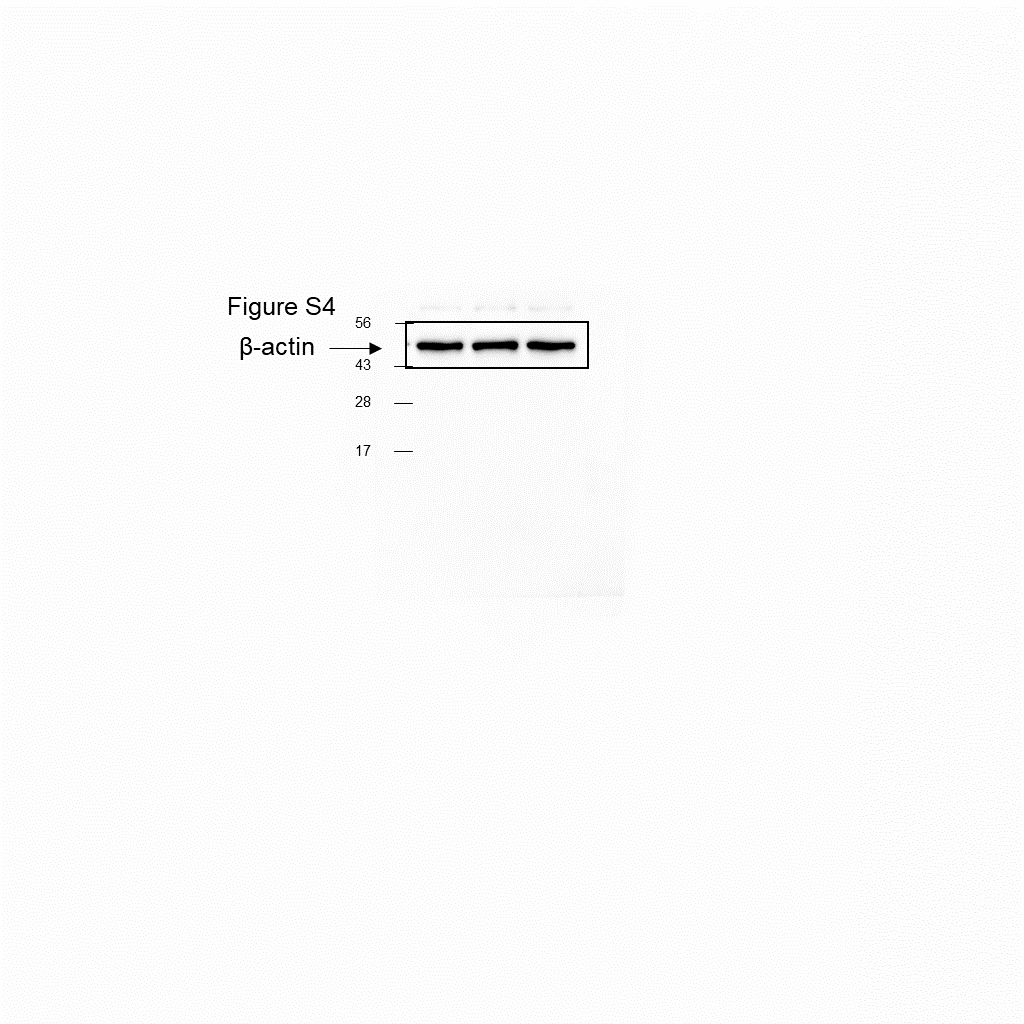


The original Full-length blots of the Supple Fig.10B


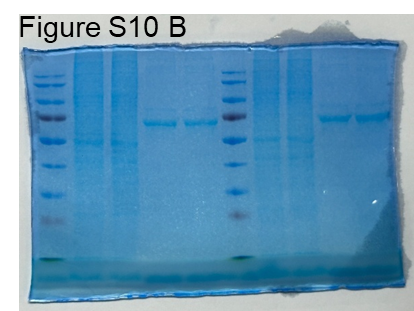

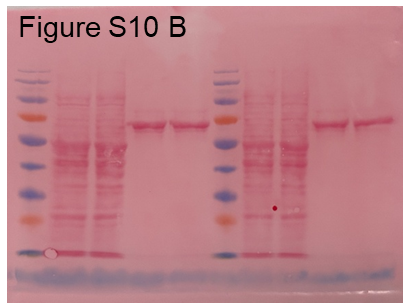

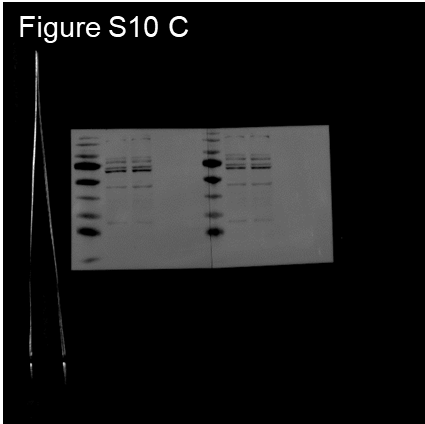

Supplement: Supplementary file 1 — Supporting File 1: advs76717‐sup‐0001‐SuppMat.docx. [file ADVS-9999-e76717-s001.docx]
