# Supplementary material for: Human Dental Pulp Stem Cell Secretome Restores Ischemic Stroke–Impaired Motor and Cognitive Functions by Reprogramming Redox and Inflammatory Signaling
Source: Adv Sci (Weinh). 2026 Jul 23:e76717. Online ahead of print. doi: 10.1002/advs.76717 (PMC13393272; doi:10.1002/advs.76717)
Supplement: Supplementary file 2 — Supporting File 2: advs76717‐sup‐0002‐SuppTable.docx. [file ADVS-9999-e76717-s002.docx]

**Table S1.** List of proteins assigned to the GO biological process terms enriched in the hDPSC secretome

| **Term** | **Genes** |
| --- | --- |
| Regulation of cell migration | ITGB1, APP, NRP1, CLIC4, GRN, SEMA7A, LRP1, SERPINE2, CSF1, LAMA4, RTN4, CORO1C, LGALS3, PERP, C1QBP,  LMNA, DAG1, TMSB10, JAM3, CCBE1, IGFBP5, FN1, LAMB1,  MIF, SOD2, SLURP1, DCN, AGT, GREM1, MMP14, ADAM9,  CDH13, CALR, GAS6, NBL1 |
| Wound healing | ITGB1, PDIA3, FGA, SERPINE2, MYOF, ANXA5, FN1, PPL,  MYL12B, COL5A1, AXL, PTK7, DAG1, SERPING1, CD59,  TLN1, GAS6, MYL9 |
| Cellular oxidant detoxification | GSTP1, GSR, APOA4, SOD2, PRDX6, CP, TXNDC17 |
| Regulation of immune system process | APP, GRN, SEMA7A, DDX3X, CSF1, TNFRSF11B, RTN4,  LGALS3, PSMB4, RPS19, PERP, C1QBP, LAMP2, RPS3, CD59, BANF1, LDLR, YWHAG, JAM3, HSPA9, PRNP, APOA2, FN1,  HLA-A, MIF, GREM1, MMP14, NAGK, RAET1G, PITHD1,  AXL, CDC37, SERPING1, DNASE2, CALR, EZR, GAS6, NBL1 |
| Regulation of apoptotic process | ITGB1, APP, NRP1, GRN, DDX3X, GSTP1, RTN4, MYDGF,  LGALS3, CTSL, PERP, C1QBP, ARHGDIA, CTSK, LMNA,  RPS3, TXNDC5, PDIA3, HSPA9, FGA, PRNP, SERPINB2,  ANXA5, AKR1A1, MIF, SOD2, EIF2S1, AGT, GREM1, DDB1,  HNRNPK, AXL, APEX1, CALR, GAS6 |
| Positive regulation of neurogenesis | ITGB1, NRP1, SEMA7A, VCAN, SERPINE2, MAP1B,  CAPRIN1, DAG1, FN1, HAPLN1 |
| Positive regulation of angiogenesis | ITGB1, GREM1, MYDGF, NRP1, CCBE1, GRN, DDAH1, RTN4 |
| Regulation of cytokine production | CCBE1, PRNP, APP, SEMA7A, DDX3X, GSTP1, APOA2, FN1,  HLA-A, IQGAP1, MIF, RTN4, AGT, RAET1G, AXL, C1QBP,  RPS3, S100A13, BANF1, EZR, GAS6 |
| Positive regulation of cell growth | MTPN, NRP1, SEMA7A, MMP14, DDX3X, MAP1B, FN1 |
| Regulation of synapse organization | ITGB1, PRNP, APP, SEMA7A, MAP1B, CAPRIN1, DAG1,  THBS2, AGRN, RTN4 |

The table lists the individual proteins corresponding to the major GO biological process terms presented in Figure 1C. These proteins were identified from the high-confidence hDPSC secretome proteins list after exclusion of serum-free DMEM-derived proteins and potential contaminants, and after filtering for proteins detected in at least two of three independent hDPSC secretome biological replicates.

**Table S2.** List of candidate functional proteins identified in hDPSC secretome and their sequence homology to mouse proteins

| **Effect** | **Symbol** | **Query Cover** | **Per. Ident** |
| --- | --- | --- | --- |
| Antioxidant activity | APOA4 | 95% | 61.07% |
|  | GSR | 100% | 82.95% |
|  | GSTP1 | 100% | 85.24% |
|  | PRDX6 | 100% | 89.73% |
|  | SOD2 | 100% | 90.09% |
|  | TXNDC17 | 100% | 80.49% |
| M2 inducer | CSF1 | 100% | 69.91% |
|  | GRN | 100% | 75.21% |
|  | LRP1 | 100% | 97.98% |
| Positive regulation of angiogenesis and neurogenesis | CCBE1 | 100% | 89.71% |
|  | DAG1 | 100% | 93.39% |
|  | DDAH1 | 100% | 93.68% |
|  | FN1 | 100% | 91.97% |
|  | GREM1 | 100% | 97.83% |
|  | GRN | 100% | 75.21% |
|  | ITGB1 | 100% | 92.48% |
|  | MYDGF | 100% | 83.82% |
|  | NRP1 | 100% | 93.17% |
|  | RTN4 | 100% | 74.50% |
|  | SEMA7A | 100% | 89.06% |
|  | SERPINE2 | 100% | 84.92% |
| ROBO | DAG1 | 100% | 93.39% |
|  | GPC1 | 100% | 88.71% |
|  | NRP1 | 100% | 93.17% |
|  | VASP | 100% | 87.63% |

List of candidate functional proteins identified in hDPSC secretome and their sequence homology to mouse proteins. Query coverage indicates the percentage of the human protein sequence that aligns with the corresponding mouse protein, while percent identity represents the proportion of identical amino acids within the aligned region.
